# The Hippo pathway effector TAZ induces intrahepatic cholangiocarcinoma in mice and is ubiquitously activated in the human disease

**DOI:** 10.1186/s13046-022-02394-2

**Published:** 2022-06-03

**Authors:** Antonio Cigliano, Shanshan Zhang, Silvia Ribback, Sara Steinmann, Marcella Sini, Cindy E. Ament, Kirsten Utpatel, Xinhua Song, Jingxiao Wang, Maria G. Pilo, Fabian Berger, Haichuan Wang, Junyan Tao, Xiaolei Li, Giovanni M. Pes, Serena Mancarella, Gianluigi Giannelli, Frank Dombrowski, Matthias Evert, Diego F. Calvisi, Xin Chen, Katja Evert

**Affiliations:** 1 Institute of Pathology, University of Regensburg, Franz-Josef-Strauß-Allee 11, Regensburg, Germany; 2 Department of Medical, Surgical and Experimental Sciences, University of Sassari, Sassari, Italy; 3 Department of Bioengineering and Therapeutic Sciences and Liver Center, University of California, 513 Parnassus Avenue, San Francisco, CA USA; 4 Department of Pathology, Eastern Hepatobiliary Surgery Hospital, Second Military Medical University, Shanghai, China; 5 Institute of Pathology, University of Greifswald, Greifswald, Germany; 6 Experimental Pathology Unit, Department of Biomedical Sciences, University of Cagliari, Cagliari, Italy; 7 School of Traditional Chinese Medicine, Capital Medical University, Beijing, China; 8 School of Life Sciences, Beijing University of Chinese Medicine, Beijing, China; 9 Liver Transplantation Division, Department of Liver Surgery, West China Hospital, Sichuan University, Chengdu, China; 10 Department of Thyroid and Breast Surgery, The 960th Hospital of the PLA, Jinan, 250031 China; 11 National Institute of Gastroenterology “S. de Bellis”, Research Hospital, Castellana Grotte, Italy; 12 University of Hawaii Cancer Center, Honolulu, Hawaii USA

**Keywords:** Intrahepatic cholangiocarcinoma, Hippo pathway, TAZ, AKT, Notch, TEAD transcription factors

## Abstract

**Background:**

Intrahepatic cholangiocarcinoma (iCCA) is a highly aggressive primary liver tumor with increasing incidence worldwide, dismal prognosis, and few therapeutic options. Mounting evidence underlines the role of the Hippo pathway in this disease; however, the molecular mechanisms whereby the Hippo cascade contributes to cholangiocarcinogenesis remain poorly defined.

**Methods:**

We established novel iCCA mouse models via hydrodynamic transfection of an activated form of transcriptional coactivator with PDZ-binding motif (TAZ), a Hippo pathway downstream effector, either alone or combined with the myristoylated AKT (myr-AKT) protooncogene, in the mouse liver. Hematoxylin and eosin staining, immunohistochemistry, electron microscopy, and quantitative real-time RT-PCR were applied to characterize the models. In addition, *in vitro* cell line studies were conducted to address the growth-promoting roles of TAZ and its paralog YAP.

**Results:**

Overexpression of TAZ in the mouse liver triggered iCCA development with very low incidence and long latency. In contrast, co-expression of TAZ and myr-AKT dramatically increased tumor frequency and accelerated cancer formation in mice, with 100% iCCA incidence and high tumor burden by 10 weeks post hydrodynamic injection. AKT/TAZ tumors faithfully recapitulated many of the histomolecular features of human iCCA. At the molecular level, the development of the cholangiocellular lesions depended on the binding of TAZ to TEAD transcription factors. In addition, inhibition of the Notch pathway did not hamper carcinogenesis but suppressed the cholangiocellular phenotype of AKT/TAZ tumors. Also, knockdown of YAP, the TAZ paralog, delayed cholangiocarcinogenesis in AKT/TAZ mice without affecting the tumor phenotype. Furthermore, human preinvasive and invasive iCCAs and mixed hepatocellular carcinoma/iCCA displayed widespread TAZ activation and downregulation of the mechanisms protecting TAZ from proteolysis.

**Conclusions:**

Overall, the present data underscore the crucial role of TAZ in cholangiocarcinogenesis

**Supplementary Information:**

The online version contains supplementary material available at 10.1186/s13046-022-02394-2.

## Background

Intrahepatic cholangiocarcinoma (iCCA) is a rare form of liver cancer, accounting for ~10-15% of all primary hepatic tumors. Nonetheless, the incidence of iCCA has been rapidly increasing in the United States and Europe in the past decades, mainly for unknown reasons [1, 2]. Importantly, data from the Surveillance, Epidemiology, and End Results (SEER) program and the United States Vital Statistics databases indicate that the rise in iCCA incidence is a true phenomenon rather than a consequence of improved diagnosis or increased detection rate [3].

iCCA is a deadly disease with few treatment options [1–5]. Therefore, to significantly improve the prognosis of iCCA patients, a better understanding of the molecular pathogenesis of this tumor type is highly required.

The Hippo signaling is an evolutionally conserved cascade controlling organ size, tissue regeneration, stem cell self-renewal, and cancer development [6–8]. It is a core kinase cascade responding to various stimuli, including cell contact, mechano-transduction signals, transmembrane receptor stimuli, and unknown factors. The central kinases of the Hippo cascade are MST1 and MST2, which directly phosphorylate the second set of kinases, LATS1 and LATS2. An adaptor protein, SAV1, favors the functional interaction between MST and LATS proteins [6–8]. The principal effectors of the Hippo pathway are Yes-associated protein (YAP) and transcriptional coactivator with PDZ-binding motif (TAZ). YAP and TAZ function as transcriptional coactivators and interact with TEA domain (TEAD) DNA binding proteins in the nucleus to initiate the expression of target genes involved in cell proliferation and survival, such as *CTGF*, *Survivin*, and *Cyr61* [9–12]*.* When active, the Hippo kinase cascade promotes LATS1/2-mediated phosphorylation of YAP and TAZ at S127 and S89 residues, respectively. Once phosphorylated, YAP and TAZ cannot translocate to the nucleus to exert their transcriptional, growth-promoting activity and are sequestered in the cytoplasm, where they bind to the 14-3-3 adaptor proteins and are primed to degradation via the proteasome system [6–12].

Cumulating evidence indicates the crucial role of the Hippo pathway in the physiology and carcinogenesis of the liver [13, 14]. For instance, previous studies showed that hepatic overexpression of YAP in the mouse causes hepatomegaly and, eventually, hepatocellular carcinoma (HCC) [15, 16]. Importantly, restoration of YAP levels after a period of overexpression triggered a rapid reversal of the hepatomegaly in mice, thus indicating that the Hippo signaling is an essential regulator of liver size [15, 16]. A similar phenotype characterizes mouse liver knockouts of various Hippo pathway members, including *MST1/2*, *Sav1/WW45*, and neurofibromatosis type 2 (*NF2*) genes [17–21]. Indeed, these models universally exhibited increased nuclear accumulation of YAP and developed liver overgrowth due to unconstrained cellular proliferation, ultimately leading to liver cancer development [17–21]. In addition, recent evidence demonstrated that Hippo pathway activity is essential for maintaining a differentiated hepatocyte state in liver cell fate [22]. Specifically, suppressing the Hippo cascade *in vivo* was sufficient to dedifferentiate adult hepatocytes into cells with progenitor features that possess self-renewal and engraftment capacity [22]. Of note, the canonical Notch signaling pathway is a crucial downstream effector of YAP in this process [22]. In liver cancer, YAP overexpression occurs in HCC, iCCA, and hepatoblastoma (HB) [23–25]. In addition, increased YAP activity is an early oncogenic event in rat and human liver carcinogenesis [26]. Also, unconstrained activation of YAP is associated with a genetic predisposition to HCC development in rats and an adverse outcome in human liver cancer [27].

Different from YAP, the available information on the role of TAZ in liver cancer is very limited. TAZ expression significantly correlates with aggressive HCC features, including tumor size, TNM stage, lymph node or distant metastasis, histological differentiation, recurrence, and poor prognosis [28]. Furthermore, forced overexpression of TAZ promotes cell proliferation, migration, and invasion of HCC cell lines *in vitro*, whereas opposite effects accompany TAZ knockdown [28, 29]. In iCCA, recent studies indicate that TAZ expression is more pronounced in tumor tissues than in the peritumoral counterpart [30, 31]. Also, high levels of TAZ are associated with a lower overall survival rate of iCCA patients after partial liver resection [30]. In addition, the simultaneous expression of TAZ and YAP correlates with chromosomal instability in this tumor type [32].

In the present study, we demonstrate that TAZ, in combination with AKT, induces the development of aggressive tumors with cholangiocellular features in the mouse liver. In addition, activation of TAZ is ubiquitous in human iCCA specimens. Altogether, the present data underline the crucial role of TAZ in cholangiocarcinogenesis and suggest that targeting TAZ might be an effective therapeutic strategy for the treatment of human iCCA.

## Materials and Methods

### Constructs and reagents

The plasmids used in the study, including pT3-EF1α-TAZS89A, pT3-EF1α-TAZS89AS51A, pT3-EF1α-HA-myr–AKT, pT3-EF1α-V5-dnRBP-J, pCMV empty vector, and pCMV/sleeping beauty transposase, have been previously described in detail [25, 33–35]. For YAP knockdown studies *in vivo*, the pT3-EF1a-AKT-shLuc (control) and pT3-EF1a-AKT-shYap plasmids were generated. The shLuc and shYAP sequences are AGGAATTATAATGCTTATCTA, and AAGCGCTGAGTTCCGAAATCT, respectively. All plasmids were extracted using the Endotoxin Free Prep Kit (Sigma-Aldrich, St. Louis, MO, USA).

### Mouse experiments

Wild-type female *FVB/N* mice were from the Jackson Laboratory (Sacramento, CA). At six to eight weeks of age, mice were subjected to hydrodynamic tail vein injection, as described previously [35], to induce iCCA formation. To determine the oncogenic potential of TAZS89A, 20μg of pT3-EF1α-TAZS89A, either alone or combined with 20μg pT3-EF1α-HA-myr-AKT, were mixed with pCMV/sleeping beauty transposase at a ratio of 25:1 and injected into 6- to 8-week–old FVB/N mice via the lateral tail vein. To investigate if TAZ oncogenic properties reside in its transcriptional function, 20μg of pT3-EF1α-TAZS89AS51A were injected into *FVB/N* mice along with 20μg pT3-EF1α-HA-myr-AKT and 1.6μg pCMV/sleeping beauty transposase. To suppress the canonical Notch pathway, we delivered hydrodynamically high doses of dnRBP-J (60 μg), pT3-EF1α-TAZS89A (20 μg), and pT3-EF1α-HA-myr-AKT (20 μg) plasmids. Furthermore, to determine if YAP is indispensable for TAZ-driven cholangiocarcinogenesis, we injected 20μg of pT3-EF1α-TAZS89A and 20μg pT3-EF1α-HA-myr-AKT, with 20 μg of either pT3-EF1a-AKT-shLuc (control) or pT3-EF1a-AKT-shYap construct into *FVB/N* mice. All animals used in the experiments were monitored continually and euthanized at specific time points, as indicated in the main text or when they became moribund. Mice were maintained and monitored following protocols approved by the Committee for Animal Research at the University of California, San Francisco (San Francisco, CA).

### Histology and immunohistochemistry

Liver specimens were harvested and fixed in 10% formalin overnight at 4°C and embedded in paraffin. Hematoxylin and eosin (ThermoFisher Scientific, Waltham, MA) staining was conducted using a standard protocol on human and mouse liver sections. Subsequently, the slides were analyzed by three expert liver pathologists (SR, ME, and KE) in a blinded fashion and according to the criteria established by Frith and Ward [36]. Immunohistochemistry (IHC) was performed as described [25]. The primary antibodies used in the study are reported in Table 1.

**Table 1:** Primary antibodies used for immunohistochemistry (IHC) and Western blot analysis (WB)

Antibody name	Use	Concentration	Company	Catalogue No.
CK19	IHC	1:400	Abcam	Ab133496
CK7	IHC	1:400	Abcam	Ab181598
HNF4α	IHC	1:100	Abcam	Ab181604
HNF1B	IHC	1:50	Proteintech	12533-1-AP
Periostin	IHC	1:100	Abcam	ab215199
Osteopontin	IHC	1;100	Abcam	ab283656
Hydroxyproline	IHC	1:100	Cell Signaling Technology	73812
SOX9	IHC	1:500	Cell Signaling Technology	82630
Vimentin	IHC	1:1000	Abcam	ab92547
Phospho-TAZ^Ser89^	IHC	1:100	Cell Signaling Technology	59971
V5-tag	IHC	1:100	Abcam	ab95038
Yap	IHC; WB	1:100; 1:1000	Cell Signaling Technology	14074
α-SMA	IHC	1:400	Abcam	ab5994
TAZ/WWTR1	IHC	1:100	Sigma-Aldrich	AMab90730
TAZ	IHC	1:200	Cell Signaling Technology	72804
Yap/Taz	WB	1:1000	Cell Signaling Technology	8418
Notch1	IHC	1:100	Cell Signaling Technology	3608
Notch2	IHC	1:50	Cell Signaling Technology	5732
JAGGED1	IHC	1:100	Cell Signaling Technology	70109
Phospho-AKT^Ser473^	IHC	1:250	Cell Signaling Technology	4060
Phospho-GSK-3β^Ser9^	IHC	1:200	Cell Signaling Technology	9323
Phospho-RPS6^Ser235/236^	IHC	1:400	Cell Signaling Technology	4856
FOXA1	IHC	1:400	Abcam	ab170933
FOXA2	IHC	1:200	Abcam	ab108422
CEBPA	IHC	1:100	Cell Signaling Technology	8178
CPS1	IHC	1:200	Abcam	ab129076
CYP2E1	IHC	1:200	Proteintech	19937-I-AP
CYP3A4	IHC	1:400	Proteintech	18227-1-AP
ARG1	IHC	1:500	Abcam	ab233548
GLUL	IHC	1:500	BD Biosciences	610517
CD44v6	IHC	1:100	ThermoFischer Scientific	MA1-81995
CD133	IHC	1:100	Abcam	ab271092
EPCAM	IHC	1:100	Abcam	ab221552
CD34	IHC	1:150	Abcam	Ab8158
Podoplanin	IHC	1:200	Abcam	ab256559
NCAM1/CD56	IHC	1:400	Cell Signaling Technology	99746
Ki67	IHC	1:500	Bethyl Laboratories	IHC-00375
S100A4	IHC	1:100	Cell Signaling Technology	13018
PDGFRβ	IHC	1:100	Cell Signaling Technology	4564
Phospho-LATS1/2^T1041/1079^	IHC	1:100	Abcam	11344
Cleaved Caspase 3	IHC	1:50	Cell Signaling Technology	9661
Cleaved PARP	IHC	1:100	Cell Signaling Technology	94885
CD4	IHC	1:100	Cell Signaling Technology	25229
CD45	IHC	1:100	Cell Signaling Technology	70257
F4/80	IHC	1:200	Cell Signaling Technology	70076
β-Actin	WB	1:5000	Santa Cruz Biotechnology	sc-10731

### Cell lines, cell culture, and *in vitro* studies

The RBE, KKUM-213, and HuccT1 human iCCA cell lines were used in this study. Cells were cultured either in Dulbecco's modified Eagle medium (DMEM; Sigma-Aldrich, St. Louis, MO; KKUM-213) or RPMI (Sigma-Aldrich; RBE and HuccT1) with 10% fetal bovine serum (FBS) (Sigma-Aldrich) and 1% penicillin-streptomycin (PS) (Sigma-Aldrich), and incubated at 37°C, 5% CO2. For gene silencing experiments, RBE and KKUM-213 cell lines were transfected with siRNA against human TAZ (# s24789) and/or YAP (# s20366), or scrambled siRNA (# s4390846, negative control) (Thermo Fisher Scientific, Waltham, MA) using the Lipofectamine RNAiMAX Transfection Reagent (Thermo Fisher Scientific) according to the manufacturer's protocol. Transient transfection experiments using pT3-EF1α-TAZS89A and pT3-EF1α-TAZS89AS51A plasmids were conducted in the HuccT1 cell line using the Lipofectamine 2000 Reagent (Thermo Fisher Scientific) following the manufacturer's protocol. Cell proliferation was assessed in the three iCCA cell lines at the 48-hour time point using the BrdU Cell Proliferation Assay Kit (Cell Signaling Technology, Danvers, MA), following the manufacturer's instructions. All experiments were repeated three times in triplicate.

### Western Blot Analysis

We homogenized cell pellets in Mammalian Protein Extraction Reagent (ThermoFisher Scientific) containing the phosphatase inhibitors (Phosphatase Inhibitor Cocktail, Sigma-Aldrich). Protein concentrations were determined with the Bio-Rad Protein Assay kit (Bio-Rad, Hercules, CA), using bovine serum albumin as a standard. Subsequently, proteins were denatured by boiling in Tris-Glycine SDS Sample Buffer (Life Technologies, Carlsbad, CA) for Western blot analysis. Proteins were separated by SDS PAGE and transferred onto nitrocellulose membranes (Life Technologies) by electroblotting. Equal protein loading was assessed by subjecting the membranes to Ponceau S Red (Sigma-Aldrich) reversible staining. Next, membranes were blocked in 5% non-fat dry milk for 1 h and then incubated with specific primary antibodies (Table 1). Finally, membranes were incubated with horseradish peroxidase-secondary antibodies (Jackson Immunoresearch Laboratories Inc., West Grove, PA, USA) diluted 1:5000 for 30 min. The Super Signal West Femto (Pierce Chemical Co., New York, NY) was used to reveal protein bands.

### RNA extraction and quantitative real-time reverse transcriptase-polymerase chain reaction (qRT-PCR)

Total mRNA from liver tissues and cells was extracted using the Quick RNA Miniprep kit (Zymo Research, Irvine, CA, USA). Thereafter, mRNA expression of the genes of interest was assessed by quantitative real-time polymerase chain reaction (qRT-PCR) using validated Gene Expression Assays for human and mouse genes (ThermoFisher Scientific; Table 2). PCR reactions were performed with 100 ng of cDNA of the collected samples or cell lines, using an ABI Prism 7000 Sequence Detection System with TaqMan Universal PCR Master Mix (Applied Biosystems). Cycling conditions were as follows: denaturation at 95°C for 10 min, 40 cycles at 95°C for 15 s, and then extension at 60°C for 1 min. Quantitative values were calculated using the PE Biosystems Analysis software and expressed as N target (NT). NT = 2^-ΔCt^, where each sample's ΔCt value was calculated by subtracting the average Ct value of the target gene from the average Ct value of the GAPDH gene.

**Table 2:** Validated Gene Expression Assays used for real-time qRT-PCR experiments

Name	Catalog number
Mouse Ccn1	Mm01192933_g1
Mouse Ccn2	Mm00487498_m1
Mouse Notch2	Mm00803077_m1
Mouse Wwtr1	Mm01289583_m1
Mouse β-Actin	Mm00607939_s1
Human WWTR1	Hs00210007_m1
Human YAP1	Hs00902712_g1
Human CNN2	Hs00170014_m1
Human CNN1	Hs00155479_m1
Human NOTCH2	Hs01050702_m1
Human β-Actin	Hs01060665_g1

### Lineage tracing and electron microscopy experiments

R26R-EYFP mice were purchased from Jackson Laboratory (Sacramento, CA). Adeno-associated virus encoding Cre-recombinase under the control of hepatocyte-specific thyroxine-binding globulin (Tbg) promoter (AAV8-Tbg-Cre) was provided by the University of Pennsylvania Vector Core (Philadelphia, PA). For electron microscopy studies, 1-2 mm^3^ AKT/TAZ liver specimens were cut with a razor blade, fixed in 2.5% glutaraldehyde (Sigma-Aldrich), postfixed with osmium tetroxide (Carl Roth, Karlsruhe, Germany), embedded in glycid ether 100 (Carl Roth), and cut with the diamond knife of a Leica Ultracut UCT ultramicrotome (Leica Biosystems, Wetzlar, Germany). Ultrathin sections of 70-90 nm were stained with uranyl acetate and lead citrate (both Sigma-Aldrich) and examined with a Zeiss Libra 120 electron microscope (Carl Zeiss Microscopy, Berlin, Germany). Periodic acid-Schiff (PAS) staining was used to identify hepatocytes with low glycogen content.

### Human Tissue Samples

Human iCCA and corresponding surrounding non-tumorous liver tissues (n=38 for each group) were collected at the Universities of Greifswald (Greifswald, Germany) and Regensburg (Regensburg, Germany). The clinicopathological features of iCCA patients are summarized in Table 3. The local Ethical Committee of the Medical Universities of Greifswald (approval code: BB 67/10) and Regensburg (approval code: 17-1015-101) provided the Institutional Review Board approval, in compliance with the Helsinki Declaration. Written informed consent was obtained from all individuals.

**Table 3:** Clinicopathological features of iCCA patients

Variables	
No. of patients	50
Male	20
Female	30
Age (years)	
<60	15
≥60	35
Etiology	
HBV	13
HCV	9
Hepatolithiasis	8
PSC	1
NA	19
Liver cirrhosis	
Yes	20
No	30
Tumor differentiation	
Well	20
Moderately	19
Poorly	11
Tumor size (cm)	
<5	40
>5	10
Tumor number	
Single	37
Multiple	13
Prognosis	
Better	
(≥ 3 years)	14
Poorer	
(< 3 years)	36
Lymph node metastasis	
Yes	21
No	29
Lung metastasis	
Yes	6
No	44

*Abbreviations*: *NA* not available, *PSC* primary sclerosing cholangitis

### Statistical Analysis

Data were analyzed using the Prism 9.0 software (GraphPad, San Diego, CA). Comparisons between the two groups were performed using the Mann-Whitney U test for non-parametric data or the Unpaired t-test for parametric data. Survival curves were analyzed using the Log-rank (Mantel-Cox) test. Data are presented as mean ±SD. P < 0.05 was considered statistically significant.

### Graphical work

Schemes and graphical representations were created using the BioRender.com software.

## Results

### Overexpression of TAZ alone rarely induces the development of intrahepatic cholangiocarcinomas in mice

**Fig. 1:**
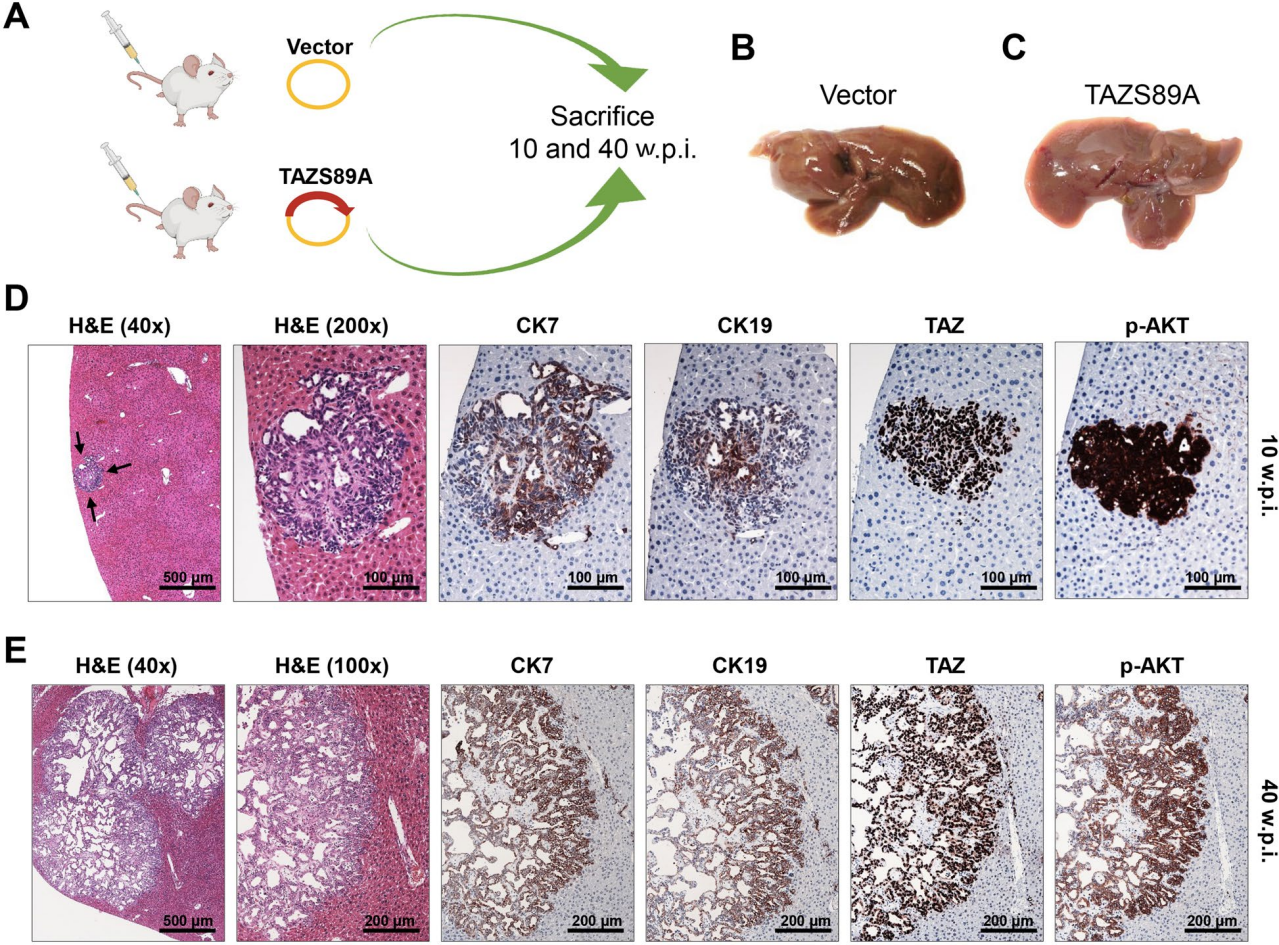
Overexpression of constitutively activated TAZ (TAZS89A) rarely induces intrahepatic cholangiocarcinoma development in mice. **(A)** Scheme of the experiments conducted. Specifically, FVB/N mice were hydrodynamically injected with the empty vector (Vector; n=10) or an activated form of TAZ (TAZS89A; n=40). Five empty vector-injected mice, and 15 TAZS89A-injected mice were sacrificed 10 weeks post-injection, whereas 5 empty vector-injected and 25 TAZS89A-injected mice were killed 40 weeks post-injection. **(B,C)** Macroscopic images of empty vector- **(B)** and TAZS89A-injected **(C)** mouse livers at 10 weeks post-injection showing a normal appearance. **(D)** Development of a single tumor in a TAZS89A-injected mouse sacrificed 10 weeks post-injection. **(E)** Large tumor developed in a TAZS89A-injected mouse sacrificed 40 weeks post-injection. Tumors depicted in **(D)** and **(E)** exhibited histological features of intrahepatic cholangiocarcinomas, with cancer cells organized in tubular and pseudoglandular structures surrounded by desmoplastic stroma. In addition, the lesions displayed robust immunoreactivity for cytokeratin (CK) 7 and 19, two biliary markers, further corroborating their cholangiocellular differentiation. In addition, the same lesions were characterized by pronounced nuclear immunoreactivity for TAZ protein. Furthermore, the lesions exhibited remarkable positiveness for activated/phosphorylated (p-)Akt: Original magnification: 40x, 100x, and 200x, as indicated in the pictures. Scale bars are indicated in the pictures. Abbreviations: H&E, hematoxylin and eosin staining; w.p.i, weeks post-injection

To determine whether TAZ deregulation is oncogenic in the liver, we overexpressed an activated form of human TAZ (TAZ S89A, which avoids the phosphorylation by LATS proteins inducing the proteasomal degradation of TAZ) in the mouse liver via hydrodynamic gene delivery (Fig. 1A). Mice injected with TAZ S89A (which will be referred to as TAZ mice) were harvested 10 and 40 weeks post-injection. Macroscopically and histologically, livers harvested 10 weeks post-injection (*n*= 15) were completely normal (Fig. 1C), indistinguishable from livers injected with the empty vector (Fig. 1B). Similarly, most TAZ mice sacrificed 40 weeks post-injection exhibited a normal, unaltered liver at the macroscopical and microscopical levels. However, detailed microscopical analysis of these mice revealed the presence of a small hepatic lesion in 1 of 15 (6.7%) and large, multiple lesions occupying most of the liver surface in 2 of 25 (8%) TAZ-injected mice harvested 10 and 40 weeks post-injection, respectively (Fig. 1 D, E). These lesions exhibited the histological features of intrahepatic cholangiocarcinoma (iCCA), consisting of glandular and tubular structures. The cholangiocellular origin of the lesions was further confirmed by the positive immunoreactivity for the biliary markers cytokeratin (CK) 7 and 19. The lesions expressed the TAZ protein homogeneously in the nuclei of iCCA cells. Surprisingly, we did not find any immunoreactivity for TAZ in the surrounding livers of those mice other than in biliary structures (not shown), thus indicating the disappearance of the transfected cells in these mice. Similarly, no TAZ-positive cells were detected in the remaining mice. Therefore, it is likely that TAZ-injected cells are eliminated early after hydrodynamic injection, resulting in a very low rate of iCCA development in TAZ-injected mice. In light of these findings, we hypothesized that other molecular alterations occurring in the three TAZ mice showing iCCA lesions might be responsible for the survival of TAZ-injected cells. We focused specifically on AKT, a central player in cell survival and a protooncogene often activated in mouse and human iCCA [37]. The lesions displayed strong immunoreactivity for activated/phosphorylated AKT (p-AKT), whereas the surrounding parenchyma exhibited faint or absent immunolabeling for the same protein (Fig. 1 D, E). Based on these results, we hypothesized that activation of survival pathways such as AKT might be necessary for TAZ-transfected cells to develop iCCA.

### Cooperation of TAZ with AKT induces rapid iCCA development in mice

**Fig. 2:**
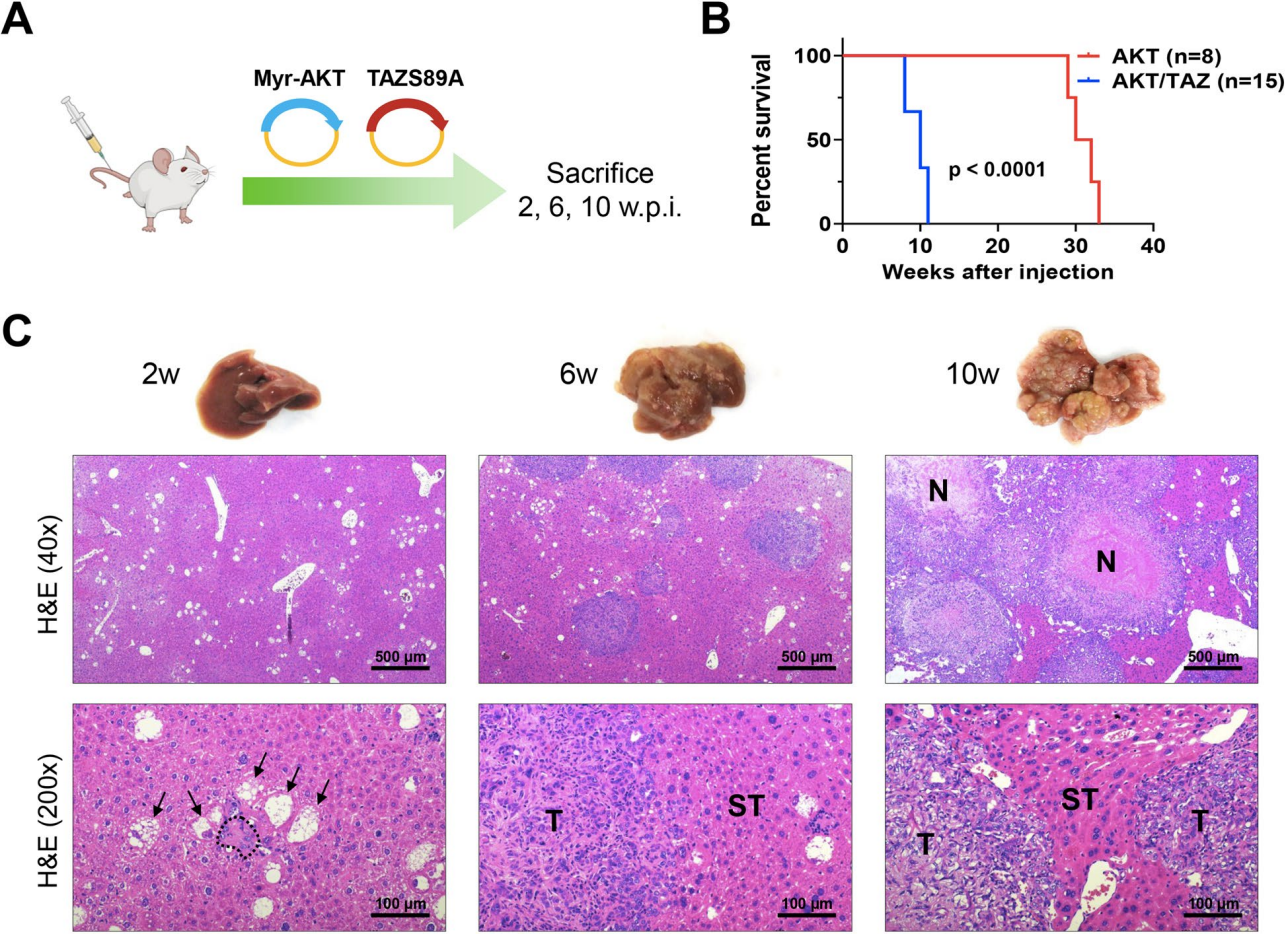
Overexpression of constitutively activated TAZ (TAZS89A) synergizes with AKT to induce intrahepatic cholangiocarcinoma development in mice. **(A)** Scheme of the experiments conducted. FVB/N mice were hydrodynamically injected with a myristoylated/activated form of AKT (Myr-AKT) and an activated form of TAZ (TAZS89A; n=25; these mice are referred to as AKT/TAZ mice). Eight mice were injected only with Myr-AKT and are referred to as AKT mice empty vector-injected mice. Five mice were sacrificed at 2 weeks post-injection and additional five mice at 6 weeks post-injection for the assessment of early lesions. Fifteen AKT/TAZ-injected mice were monitored and sacrificed 10 weeks post-injection when a high tumor burden occurred. **(B)** Survival curves of AKT and AKT/TAZ mice. **(C)** Macroscopic and microscopic images of AKT/TAZ mouse livers. Two weeks post-injections, single cells and clusters of a few cells are appreciable in the AKT/TAZ livers. Besides normal hepatocytes, large cells owing to lipid accumulation (indicated by arrows) and small cells with scarce cytoplasm (surrounded by dots) can be easily detected. By six weeks post-injection, most giant cells had disappeared, and tumors of cholangiocellular features developed the liver parenchyma. By 10 weeks post-injection, tumors had progressed, occupying most of the liver surface. Signs of biological aggressiveness, such as large areas of necrosis (N), are visible. Original magnification: 40x and 200x; scale bar: 500 μm in 40x, 100 μm in 200x. Abbreviations: H&E, hematoxylin and eosin staining; w.p.i, weeks post-injection

**Fig. 3:**
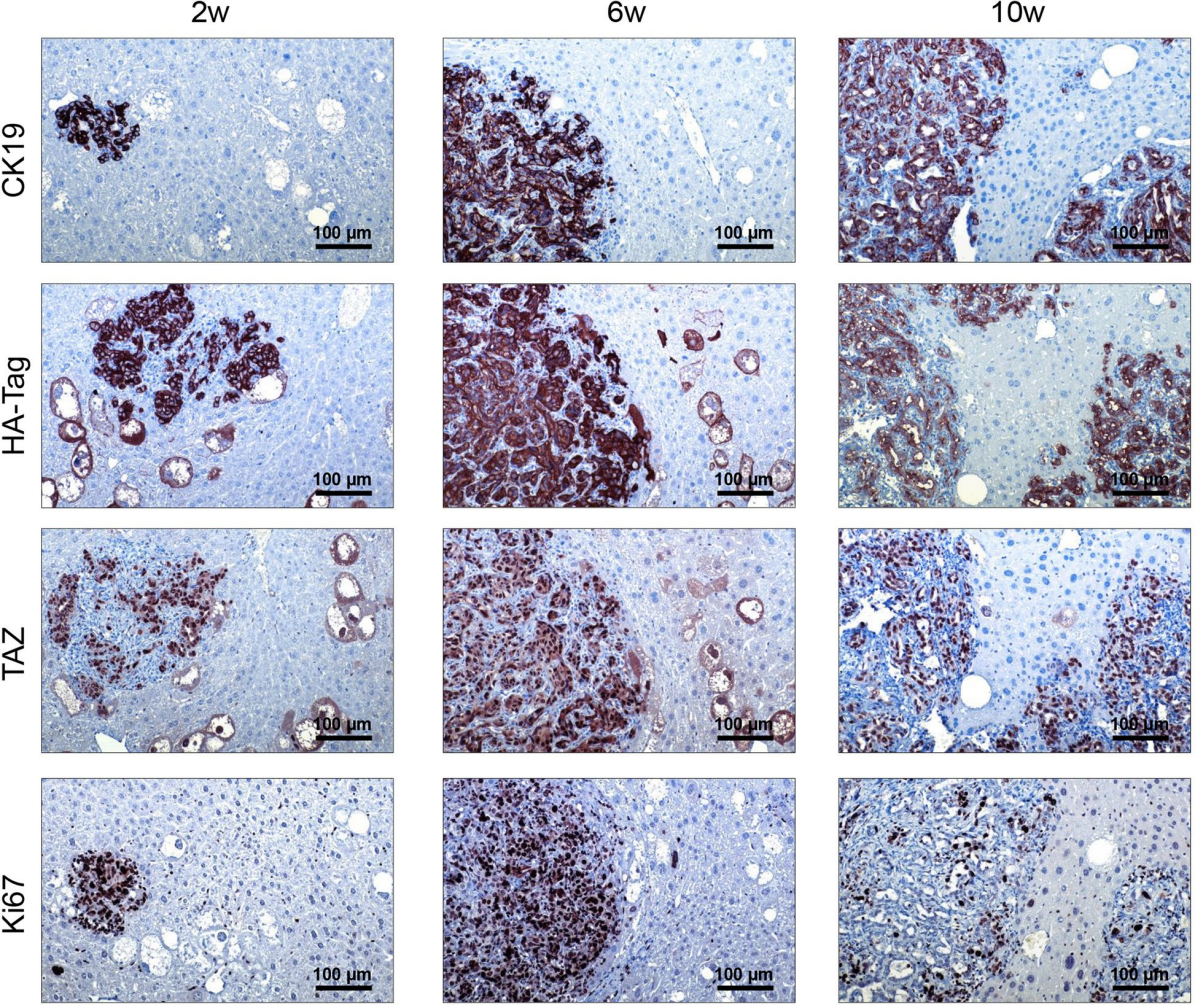
Histopathologic characterization of liver lesions developed in AKT/TAZ mice. Two weeks post-injection, large and small altered cells, either alone or in small clusters, are easily detectable on the liver. Both cell types express HA-Tag(AKT) and nuclear TAZ, implying their origin from the injected plasmids. As expected, HA-Tag immunoreactivity is limited to the transfected cells, whereas faint cytoplasmic TAZ immunolabeling is appreciable in the unaffected hepatocytes. Of note, immunoreactivity for CK19, a biliary marker, is restricted to the small cell type and normal biliary cells. These small cells are also characterized by elevated proliferation, as indicated by positivity for Ki67 staining. Presumably, due to the different proliferation properties of the two altered cell types, the small cells rapidly replace the giant cells, forming CK19-positive tumors that occupy almost the whole liver by 10 weeks post-injection. Original magnification: 200x; scale bar: 100 μm

**Fig. 4:**
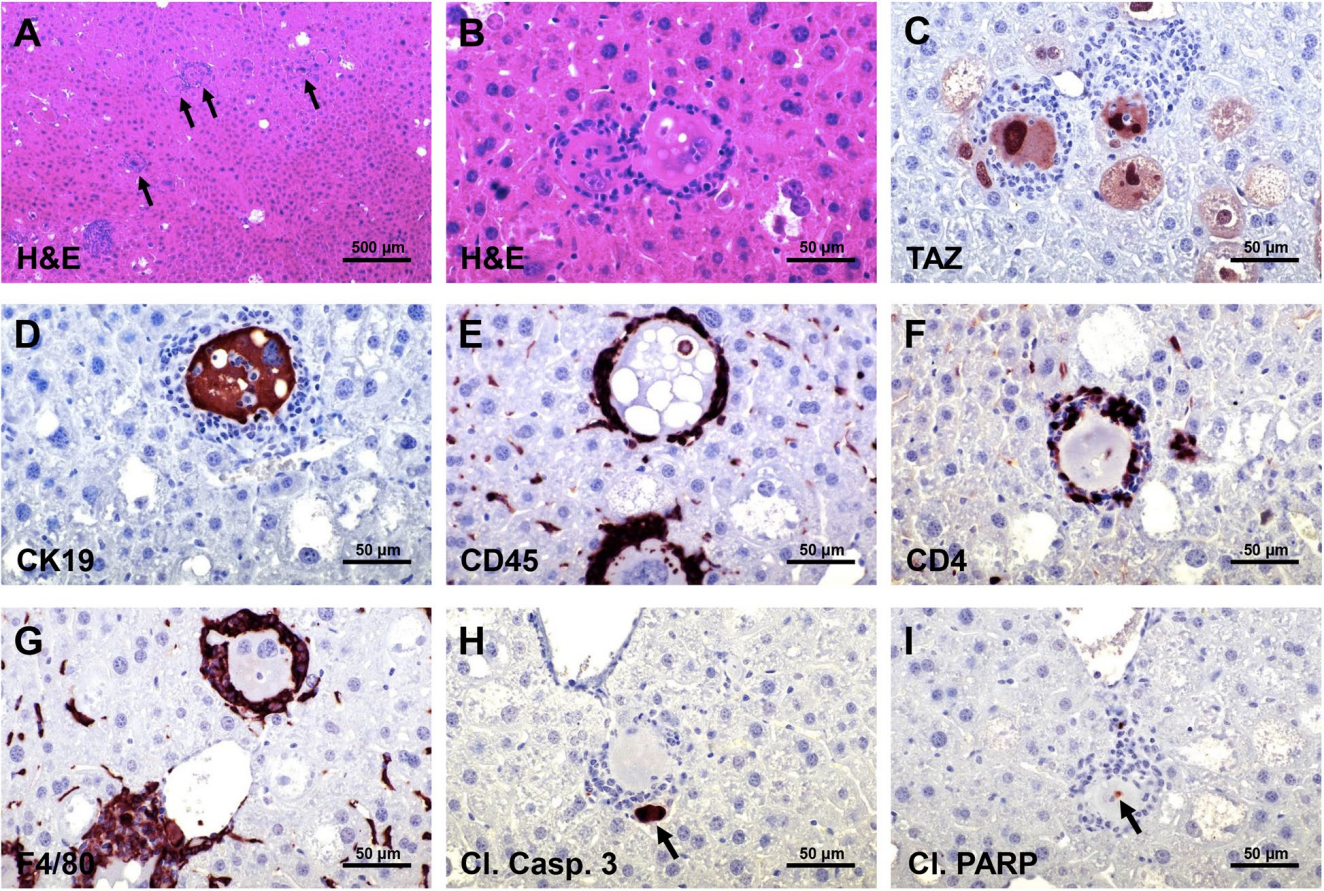
Early lesions developed in AKT/TAZ mice display an inflammatory infiltrate. (**A**) Two weeks after hydrodynamic injection of AKT and TAZ, numerous transfected cells (indicated by black arrows) were encircled by an inflammatory infiltrate. The transfected cells and the inflammatory cells could be better appreciated at higher magnification (**B**), with the inflammatory cells characterized by small spherical nuclei and forming a fence around the transfected cells. The transfected cells displayed elevated TAZ (**C**) and CK19 (**D**) immunoreactivity. The inflammatory cells consisted of lymphocytes, characterized by immunoreactivity for CD4 and CD45 markers (**E**,** F**), and macrophages (immunoreactivity for the F4/80 marker) (**G**). Some small cells encircled by the inflammatory reaction showed signs of apoptosis, as revealed by positive immunoreactivity of apoptotic bodies (indicated by black arrows) for cleaved Caspase 3 (**H**) and cleaved PARP (**I**), implying their destruction by the inflammatory response. Original magnification: 100x in (**A**), 400x in (**B**-**I**); scale bar: 200 μm in (**A**), 50 μm in (**B**-**I**).

Based on the data obtained in TAZ mice, we determined whether the co-expression of TAZ with the AKT protooncogene accelerates tumorigenesis and/or increases the incidence of iCCA in mice. Previous data from our laboratory indicate that YAP, the TAZ paralog, cannot induce cholangiocarcinogenesis alone but cooperates with AKT to drive iCCA development in mice [37]. Thus, we injected the TAZ S89A plasmid together with a plasmid containing an activated/myristoylated form of AKT (HA-tagged; the combination will be referred to as TAZ/AKT) into mice (Fig. 2A). Mice were harvested at 2 (n=5), 6 (n=5), and 10-11 (n=15) weeks post-injection. At the latest time point, mice displayed significant abdomen enlargement and required euthanasia. Mice injected only with AKT (*n*=8) were instead sacrificed between 29- and 33-weeks post-injection (Fig. 2B). Macroscopically, the livers of these mice appeared normal at 2 weeks after injection (Fig. 2C). However, small, white, cyst-like lesions were present on the liver surface after 6 weeks. These lesions rapidly expanded and, by 10-11 weeks, had replaced most of the normal liver tissue, and mice rapidly deteriorated and either succumbed or needed to be euthanized. No extrahepatic metastases developed in these mice. At the microscopical level, transfected cells were already visible and formed clusters 2 weeks after hydrodynamic injection (Fig. 2C). Of note, two distinct cell types resulted from the simultaneous transfection of AKT and TAZS89 constructs. Specifically, one cluster consisted of enlarged, clear cells due to the intracellular accumulation of lipids and glycogen, often displacing the nucleus to the cell's periphery. These cells were indistinguishable from those generated by overexpressing AKT alone in the mouse liver [38]. The second cluster was characterized by small cells with scarce basophilic cytoplasm and a high nucleus/cytoplasm ratio. As expected, both cell types displayed robust immunoreactivity for HA-tagged AKT and TAZ antibodies, implying their origin from the injected constructs (Fig. 3). Importantly, when diluting the anti-TAZ primary antibody, it was evident that the levels of TAZ immunolabeling were higher in the cholangiocyte-like small cells than in the enlarged, clear-cell hepatocytes (Supplementary Figure 1). Small cells also displayed the highest levels of SOX9, a Notch effector and a critical player in hepatocyte reprogramming and biliary commitment [39] (Supplementary Figure 1). Consequently, only the small cell type exhibited strong positivity for the biliary markers CK19 (Fig. 3) and CK7 (not shown). In addition, the proliferation marker Ki67 was significantly higher in the small cells (Fig. 3), suggesting their higher growth capacity than the enlarged, lipid-rich cells. Significantly, several small cells displaying elevated TAZ and CK19 levels were surrounded by an inflammatory infiltrate in AKT/TAZ livers two weeks after hydrodynamic injection (Fig. 4). These inflammatory cells consisted of lymphocytes (characterized by immunoreactivity for CD4 and CD45 markers) and macrophages (immunoreactivity for the F4/80 marker). Some small cells encircled by the inflammatory reaction showed signs of apoptosis (as assessed morphologically and by positive immunoreactivity for cleaved Caspase 3 and cleaved PARP), implying their destruction by the inflammatory response. Importantly, this inflammatory event was not detectable at later time points (not shown), indicating exhaustion/loss of the inflammatory response during AKT/TAZ-driven cholangiocarcinogenesis. Because such an inflammatory response does not occur in livers from mice injected only with AKT (unpublished observation), transfection of the TAZ protooncogene likely is responsible for it, for unknown reasons, leading to the elimination of many transduced cells in AKT/TAZ mice. However, despite the described inflammatory reaction, the fast-proliferating small cells rapidly replaced the lipid-rich large cells in the hepatic parenchyma, and tumors already at 6 weeks post-injection were composed only of CK19/CK7-positive cells. However, tiny clusters of large cells could still be appreciated in the mouse liver. The same pattern was observed at 10-11 weeks post-injection, with pure iCCA CK19-positive occupying most of the liver surface (Fig. 3).

### AKT/TAZ lesions resemble the histomolecular features of human intrahepatic cholangiocarcinoma

**Fig. 5:**
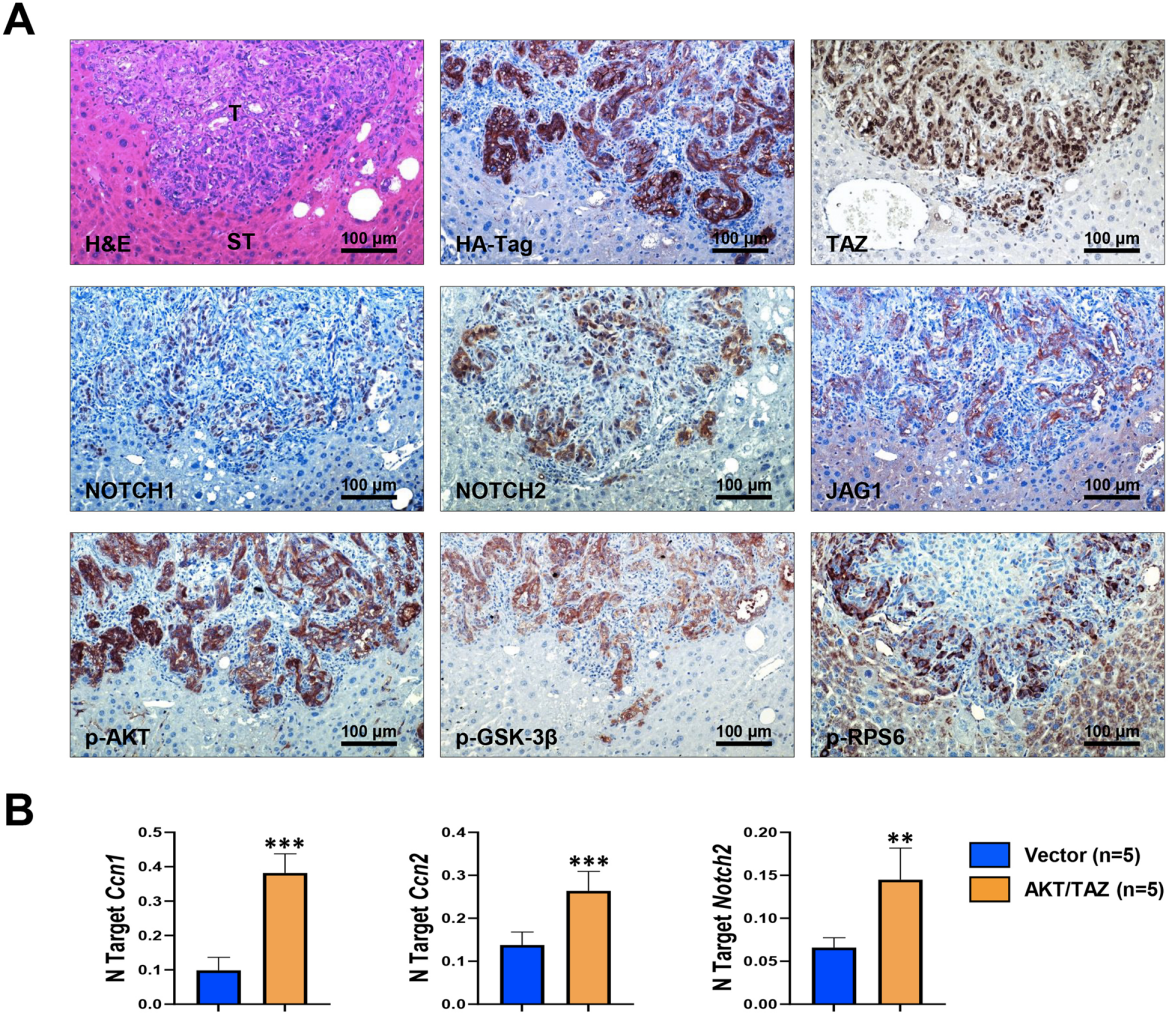
Liver lesions developed in AKT/TAZ mice display activation of Hippo and AKT/mTOR pathways. (**A**) Representative immunohistochemical patterns of a cholangiocellular tumor exhibiting immunoreactivity for HA-Tag(AKT) and nuclear TAZ, as well as for downstream effectors of the Hippo (NOTCH1, NOTCH2, and JAG1) and AKT/mTOR (phosphorylated/activated AKT or p-AKT; phosphorylated/inactivated GSK-3β or p-GSK-3β; and phosphorylated/activated RPS6 or p-RPS6) pathways. Original magnification: 200x; scale bar: 100 μm. (**B**) Upregulation of the Hippo pathway targets *CCN1*, *CCN2*, and *NOTCH*2 in AKT/TAZ livers (10 weeks post-injection) compared with livers injected with the empty vector (Vector), as assessed by quantitative real-time RT-PCR. Abbreviations: H&E, hematoxylin and eosin staining; ST, non-tumorous surrounding tissue; T, tumor. Data are expressed as means ± SD. ***p < 0.0001 and **p < 0.01 vs. empty vector-injected mice

**Fig. 6:**
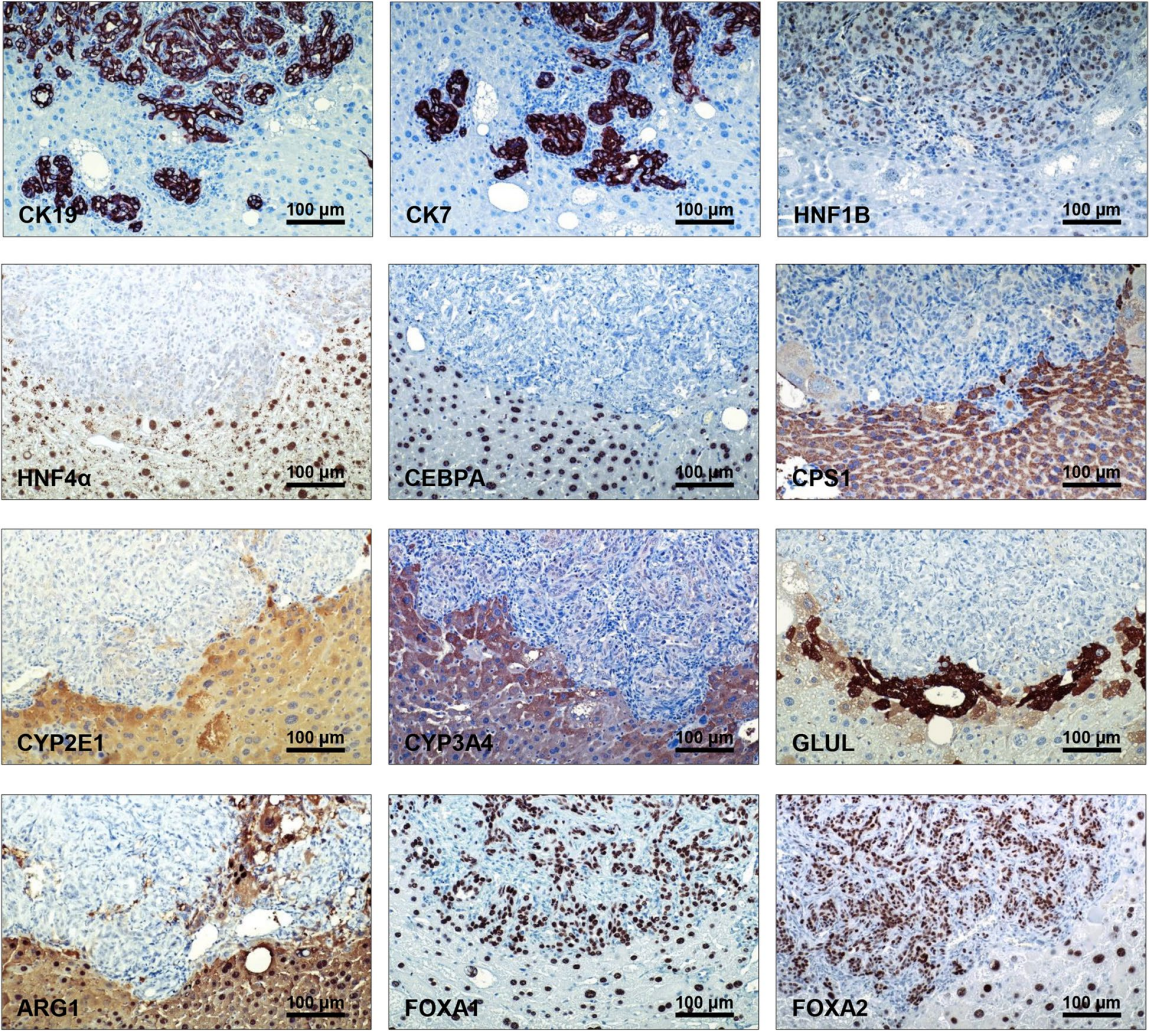
AKT/TAZ liver lesions exhibit molecular features of cholangiocarcinoma. Representative immunohistochemical patterns of an AKT/TAZ tumor showing positive immunoreactivity for cholangiocellular markers such as cytokeratin (CK) 7, CK19, and HNF1B, and weak/absent immunolabeling for hepatocellular markers, including hepatocyte nuclear factor (HNF)-4α, CCAAT/enhancer-binding protein (CEBP)-A, Carbamoylphosphat-Synthetase I (CPS1), Cytochrome P450 Family 2 Subfamily E Member 1 (CYP2E1), Cytochrome P450 3A4 (CYP3A4), glutamine synthetase (GLUL), and liver arginase (ARG1). The staining pictures are sections from the same tumor depicted in Fig. 4. Original magnification: 200x; scale bar: 100 μm

Next, we determined the molecular features of AKT/TAZ tumors by immunohistochemistry (Fig. 5A). As expected, AKT/TAZ lesions but not the surrounding non-tumorous livers displayed activation of the Notch pathway (as evaluated by NOTCH1, NOTCH2, and JAGGED1 immunoreactivity), a canonical downstream effector of the Hippo cascade responsible for iCCA development, and the AKT/mTOR signaling (phosphorylated/activated AKT or p-AKT, phosphorylated/activated ribosomal protein S6 or p-RPS6, and phosphorylated/inactivated glycogen synthase kinase 3 beta or p-GSK-3β). Increased mRNA levels of TAZ targets, including *Ccn1*, *Ccn2*, and *Notch2*, were also detected in AKT/TAZ tumors compared with livers injected with empty vector by quantitative real-time RT-PCR (Fig. 5B). Consistent with their cholangiocellular features, all tumor cells were positive for the bile duct markers CK19 and CK7 and the cholangiocyte-specific transcription factor HNF1B (Fig. 6). When examining hepatocellular characteristics, immunoreactivity for hepatocyte nuclear factor 4 alpha (HNF4α), CCAAT enhancer-binding protein alpha (CEBPA), carbamoyl-phosphate synthase 1 (CPS1 or Hep Par-19), cytochrome P450 2E1 (CYP2E1), cytochrome P450 3A4 (CYP3A4), glutamine synthetase (GLUL), and liver arginase (ARG1) were uniformly downregulated in the lesions when compared with non-neoplastic liver counterparts. In contrast, levels of forkhead box A1 and A2 (FOXA1 and FOXA2) transcription factors were equivalent in tumorous and non-tumorous livers (Fig. 6). Furthermore, AKT/TAZ tumors displayed positive staining for markers of tissue desmoplasia and collagen deposition/synthesis, prominent features of human iCCA, such as S100 calcium-binding protein A4 (S100A4), vimentin, platelet-derived growth factor receptor beta (PDGFRβ), periostin, osteopontin, alpha-smooth muscle actin (α-SMA), and hydroxyproline, in the stromal cells (Supplementary Figure 2). Also, the progenitor/stemness markers SOX9, CD44v6, CD133, and EPCAM were highest in the tumor lesions. In addition, activation of the TAZ paralog YAP (as assessed by its nuclear accumulation) was observed in AKT/TAZ tumors (Supplementary Figure 2). Furthermore, pronounced immunoreactivity for the angiogenic marker CD34 and the lymphatic vessel marker podoplanin and low levels of apoptosis (as determined by cleaved caspase 3 immunolabeling) was readily detected in AKT/TAZ cholangiocellular lesions (Supplementary Figure 3).

Taken together, the present findings indicate that activated TAZ and AKT cooperation induces cholangiocellular tumors recapitulating various histopathologic and molecular features of human iCCA.

### The interaction of TAZ with TEAD transcription factors is necessary for AKT/TAZS89A-driven cholangiocarcinogenesis

**Fig. 7:**
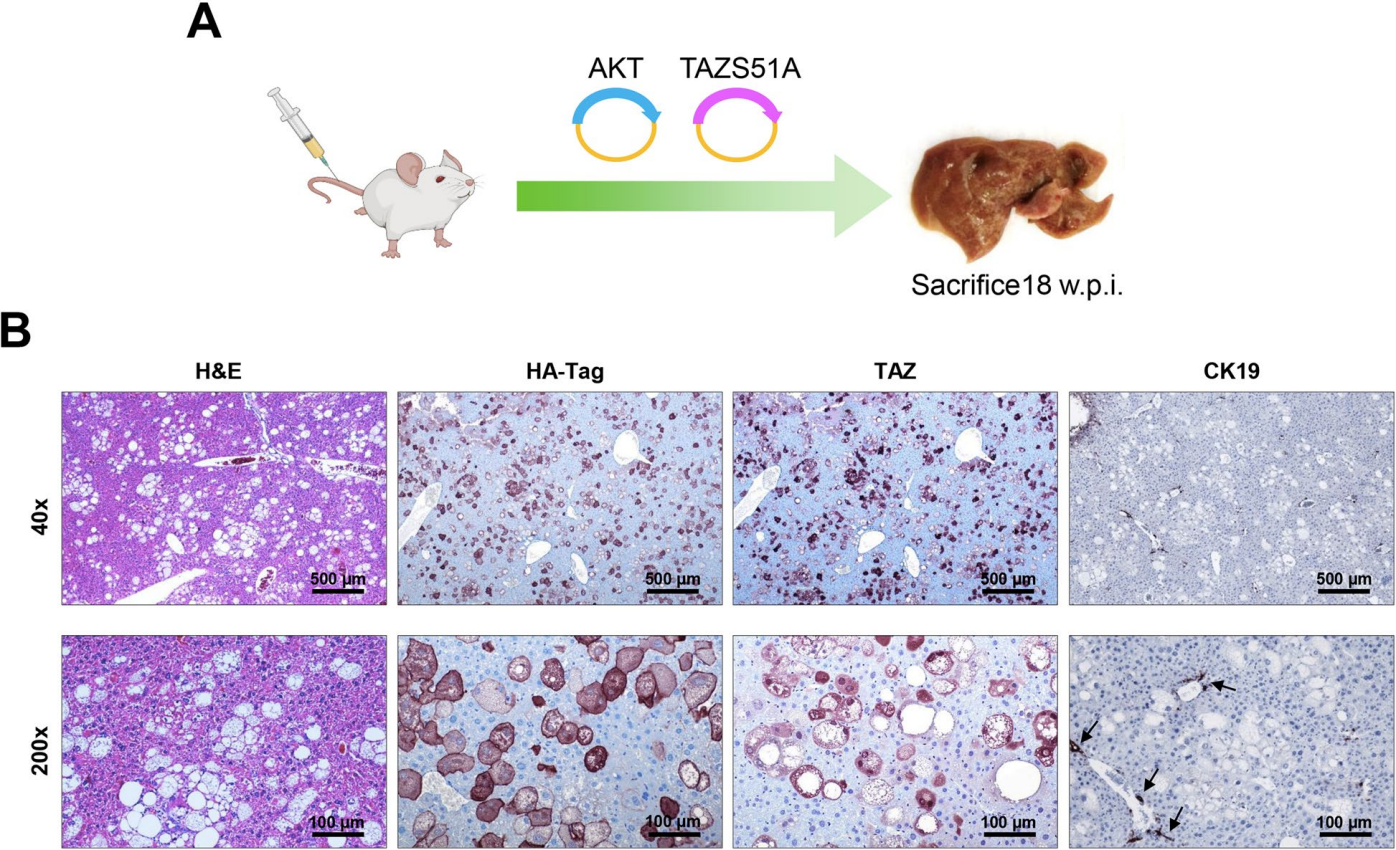
Taz oncogenic activity depends on its functional interaction with TEAD factors in AKT/TAZ mice. (**A**) Scheme of the experiments conducted. (**B**) Blockade of TAZ binding to TEAD transcription factors by hydrodynamic transfection of the TAZS89AS51A plasmid (TAZS51A) inhibits AKT/TAZ-dependent cholangiocarcinogenesis in the mouse liver. At the histological levels, the livers of AKT/TAZ mice are indistinguishable from those of mice injected only with Myr-AKT, consisting of clusters of lipid-rich giant cells. As expected, the transfected cells show positive immunoreactivity for HA-Tag(AKT) and nuclear TAZ. Due to the disappearance of cholangiocellular lesions, the staining for the biliary marker CK19 is restricted to normal biliary cells. Original magnification: 40x and 200x; scale bar: 500 μm in 40x, 100 μm in 200x. Abbreviations: H&E, hematoxylin and eosin staining.

TAZ is a transcriptional coactivator that interacts with TEAD DNA-binding proteins to drive downstream gene expression. Nevertheless, TAZ also possesses functions that do not depend on its interaction with TEAD factors [10–12]. To determine whether TAZ can trigger cholangiocarcinogenesis independent of TEADs, we co-expressed TAZS89AS51A and activated/myristoylated AKT plasmids into the mouse liver by hydrodynamic injection (n=6) (Fig. 7A). The S51A mutation prevents the binding of TAZ with TEAD proteins [33]. All AKT/TAZS89AS51A mice appeared healthy and were harvested 18 weeks after injection. Grossly, no tumor nodules could be observed in the mouse livers. Microscopically, AKT/TAZS89AS51A livers were full of large clear-cell hepatocytes due to high lipid content, thus recapitulating the phenotype of AKT-overexpressing mice [38] (Figure 7B). We further confirmed the inability of the TAZS89AS51A plasmid to induce TEAD-mediated downstream effectors *in vitro*. Indeed, forced overexpression of either TAZS89A or TAZS89AS51A triggered similar overexpression of TAZ in the human Hucct1 iCCA cell line (Supplementary Figure 4B). This cell line was selected for overexpression experiments because it displays low basal levels of TAZ (Supplementary Figure 4A). However, transient transfection of TAZS89A but not TAZS89AS51A could trigger the mRNA up-regulation in the same cells of *CCN1* and *CCN2* specific targets of TAZ (Supplementary Figure 4 D, E), whose induction is mediated by TEAD factors [10–12]. Furthermore, only forced overexpression of TAZS89A significantly increased the proliferation of HuCCT1 cells in culture (Supplementary Figure 4 F).

Overall, the present data demonstrate that the interaction between TAZ and TEAD transcription factors is required for TAZ-induced iCCA development in mice and TAZ-dependent proliferation *in vitro*.

### The canonical Notch cascade is indispensable for cholangiocellular commitment in AKT/TAZ mice

**Fig. 8:**
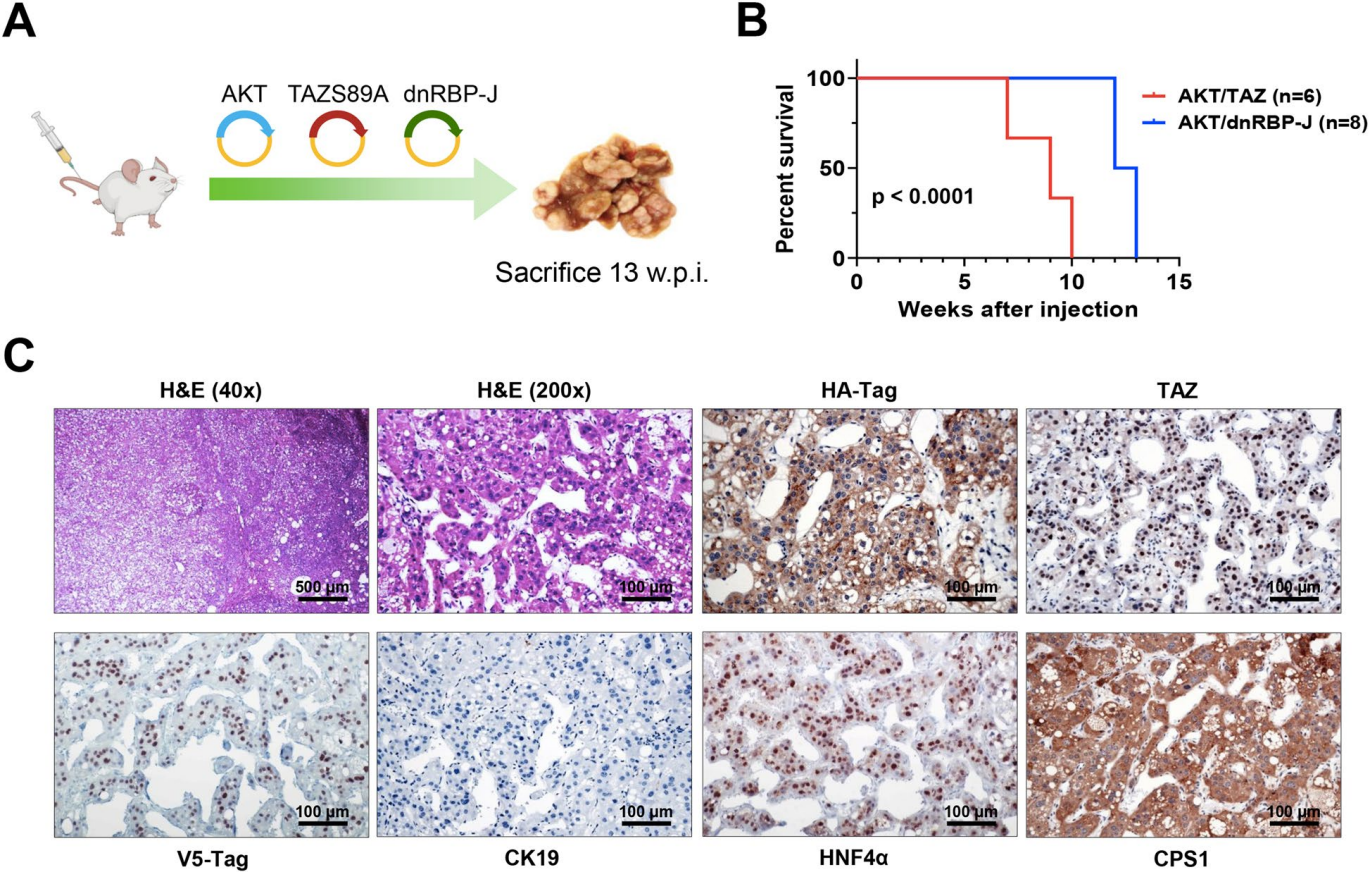
Suppression of the canonical Notch signaling does not hinder liver carcinogenesis but suppresses the cholangiocellular differentiation of AKT/TAZ lesions. **(A)** Study design. Briefly, wild-type mice were subjected to hydrodynamic tail vein injection of either AKT/TAZ/pT3 (control) or AKT/TAZ/dnRBP-J plasmids. In particular, dnRBP-J is the dominant-negative form of the transcriptional coactivator recombination signal binding protein for immunoglobulin kJ region (RBP-J), whose overexpression effectively blunts the canonical Notch cascade. **(B)** Survival curve of AKT/TAZ/pT3 (control) and AKT/TAZ/dnRBP-J mice. Note that the survival of AKT/TAZ/dnRBP-J mice is only slightly longer than that of control mice. **(C)** Liver lesions from AKT/TAZ/dnRBP-J mice consist of pure trabecular and solid hepatocellular carcinomas. Due to their hepatocellular nature, the lesions exhibit positive immunoreactivity for HNF4α and CPS1 hepatocellular markers. They are negative for the CK19 cholangiocellular marker. As expected, AKT/TAZ/dnRBP-J liver lesions express the HA-Tag(AKT), nuclear TAZ, and V5-Tag (dnRBP-J) proteins. Original magnifications: 40x and 200x; scale bar: 500 μm in 40x and 100 μm in 200x. Abbreviations: H&E, hematoxylin and eosin staining

Previous reports indicate that YAP, the TAZ paralog, induces hepatocyte-cholangiocyte transdifferentiation via the Notch pathway [22, 37]. To determine whether the same applies to TAZ, we co-injected *myr-AKT1* and *TAZS89A* plasmids with a dominant-negative form of the Notch transcriptional activator RBP-J (*dnRBP-J*; these mice will be referred to as AKT/TAZ/dnRBP-J) (Fig. 8A). Previously, we have shown that dnRBP-J effectively suppresses the canonical Notch pathway *in vitro* and *in vivo* [33, 34]. Strikingly, the simultaneous injection of *myr-AKT1, TAZS89A,* and dn*RBP-J* did not hamper tumor development in mice, which required to be sacrificed by 13 weeks post-injection due to high tumor burden (Fig. 8B). Macroscopically, numerous nodules could be appreciated on the liver surface of AKT/TAZ/dnRBP-J mice. However, when analyzing the lesions at the histopathological level, AKT/TAZ/dnRBP-J livers were characterized solely by the presence of lipid-rich preneoplastic lesions, identical to those detected in mice injected exclusively with AKT [38], and pure HCC. The hepatocellular nature of the neoplastic lesions was further confirmed by their widespread immunoreactivity for HNF4α and CPS1 hepatocyte markers and negative immunolabeling for CK19 (Fig. 8C).

Therefore, the canonical Notch pathway influences tumor cell differentiation but does not inhibit AKT/TAZ–induced carcinogenesis.

### Knockdown of YAP delays AKT/TAZ-driven cholangiocarcinogenesis

Because we detected pronounced nuclear localization of the TAZ paralog YAP in AKT/TAZ lesions (Supplementary Figure 2), we tested the importance of YAP in AKT/TAZ dependent on iCCA development. For this purpose, TAZ was co-injected into the mouse liver with a plasmid containing myristoylated AKT and the short hairpin against YAP (pT3-EF1a-AKT-shYap-TAZ mice). The AKT-shLuc-TAZ combination was used as a scrambled control (Supplementary Figure 5A). Simultaneous injection of *myr-AKT1, TAZS89A,* and shLuc resulted in a high tumor burden, and AKT-shLuc-TAZ mice required sacrifice between 12 and 14 weeks post-injection (Supplementary Figure 5B). On the other hand, suppression of YAP by shYAP significantly delayed tumorigenesis, and AKT-shYAP-TAZ mice were euthanized significantly later, by 30 weeks post-injection (Supplementary Figure 5B). Both AKT-shLuc-TAZ and AKT-shYAP-TAZ mice developed pure iCCA, histomorphologically indistinguishable (Supplementary Figure 5C). No extrahepatic metastases developed in the two models. As assessed by immunohistochemistry, AKT-shLuc-TAZ livers exhibited nuclear YAP accumulation in the neoplastic lesions, and faint/absent cytoplasmic immunoreactivity for YAP in the non-tumorous counterpart. AKT-shYAP-TAZ tumors were completely YAP negative, and low/absent cytoplasmic YAP staining was detected in AKT-shYAP-TAZ non-tumorous livers (Supplementary Figure 5C). In addition, low levels of YAP1 mRNA characterized AKT-shYAP-TAZ tumors compared to AKT-shLuc-TAZ corresponding lesions (Supplementary Figure 5D). Similar liver weight/body weight ratios between AKT-shLuc-TAZ and AKT-shYAP-TAZ were found at sacrifice (Supplementary Figure 5E).

Overall, the data indicate that YAP contributes to AKT/TAZ tumor progression without affecting the cholangiocellular phenotype of the lesions.

### Mature hepatocytes are the source of cholangiocarcinoma cells in AKT/TAZ mice

**Fig. 9:**
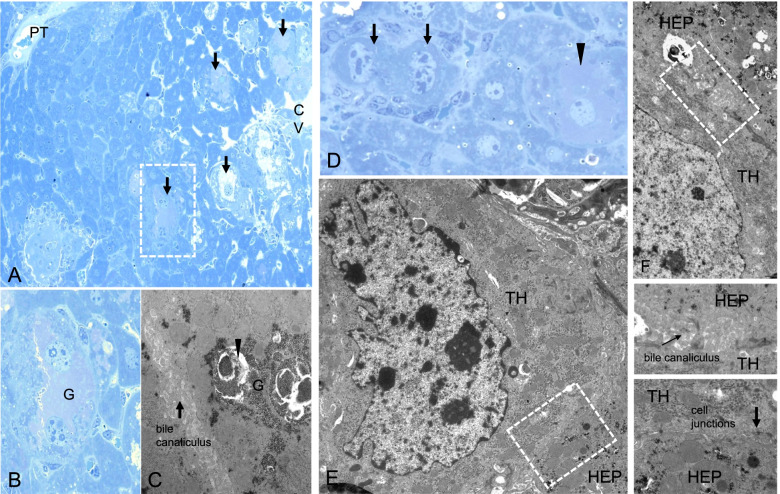
Ultrastructural alterations of transformed hepatocytes in acinar zone 3 of the liver acinus, 1 and 2 weeks after hydrodynamic tail vein injection of Akt-TAZ plasmids. **(A, B)** Liver acinus 1 week post-injection with a portal tract (PT) and altered hepatocytes (arrows) located in acinus zone 3. Hepatocytes near the central venule (CV) are often enlarged and vacuolated. Beneath these hepatocytes, single atypical, sometimes binucleated atypical hepatocytes (marked box and magnification in **B**) reveal irregular cell borders, enlarged nuclei, and several nucleoli. They are surrounded by unaltered hepatocytes with round, oval nuclei, and regular cell shapes. **(C)** Note massive glycogen **(G)** storage with tightly packed α-particles in the cytoplasm and membrane-enclosed autophagic vacuoles (arrowhead). **(D)** Two weeks after injection, some altered hepatocytes reveal noticeable loss of glycogen (arrows), whereas glycogen storing cells are still common (arrowhead). At the ultrastructural level **(E, F)**, transformed hepatocytes (TH) reveal irregularly shaped nuclei and now absent glycogen storage in contrast to normal neighboring hepatocytes (HEP). Nevertheless, cell junctions and bile canaliculi are preserved (boxed magnified areas), implying their hepatocellular nature. A, B, D Stainings according to Richardson, C, E, F staining with uranyl acetate and lead citrate. Length of the lower edge A 500μm, B 80μm, C 10μm, D 111μm, E 18μm, F 10μm.

Mounting evidence indicates that iCCA lesions can originate in mice from mature hepatocytes via a transdifferentiation process [35, 40, 41]. In agreement with these findings, a recent investigation analyzed the gene expression programs in AKT/NICD-induced hepatocyte-derived CCA at the single-cell level using scRNASeq technology [42]. Intriguingly, the authors identified an Epcam+Alb+Krt19- epithelial cluster consisting of cells with both hepatocyte and cholangiocyte features. These cells gradually evolved from normal hepatocytes to iCCA, further confirming the origin of these tumors from mature hepatocytes. In light of these findings, we determined whether the same applies to AKT/TAZ mice. By electron microscopy, we found that enlarged and irregular nuclei and loss of cytoplasmic glycogen characterized the hydrodynamically transfected cells. Nonetheless, the transfected cells were connected to the adjacent hepatocytes through cell junctions, implying their hepatocellular origin (Fig. 9). The origin of iCCA cells from mature hepatocytes in AKT/TAZ mice was further confirmed by lineage tracing experiments. Specifically, AAV8-Tbg-Cre was injected into R26R-EYFP mice, which would initiate hepatocyte-specific expression of EYFP in one week. Subsequently, the HA-tagged AKT and TAZ plasmids were delivered into the liver by hydrodynamic tail vein injection to trigger tumor development (Supplementary Figure 6). Interestingly, as assessed by co-immunofluorescence (IF) staining of the tumor sections, all AKT/TAZ tumor cells expressed CK19, a biliary cell marker, and HA-tag. In addition, the cells were positive for EYFP, indicating the hepatocyte origin of these iCCA cells (Supplementary Figure 7). Moreover, immunohistochemistry conducted on mouse livers two weeks after AKT/TAZ hydrodynamic injection revealed the presence of "hybrid" cells displaying concomitantly hepatocellular and cholangiocellular features, indicating various phases of the transdifferentiation process. For instance, some hydrodynamically transfected mature hepatocytes expressed the cholangiocellular markers CK19 and CD133, while the small cells (malignant cholangiocytes) uniformly expressed the hepatocellular marker HNF4A (Supplementary Figure 8).

Therefore, AKT/Taz-induced cholangiocellular tumors originate from mature hepatocytes.

### Ubiquitous activation of TAZ in human intrahepatic cholangiocarcinoma

**Fig. 10:**
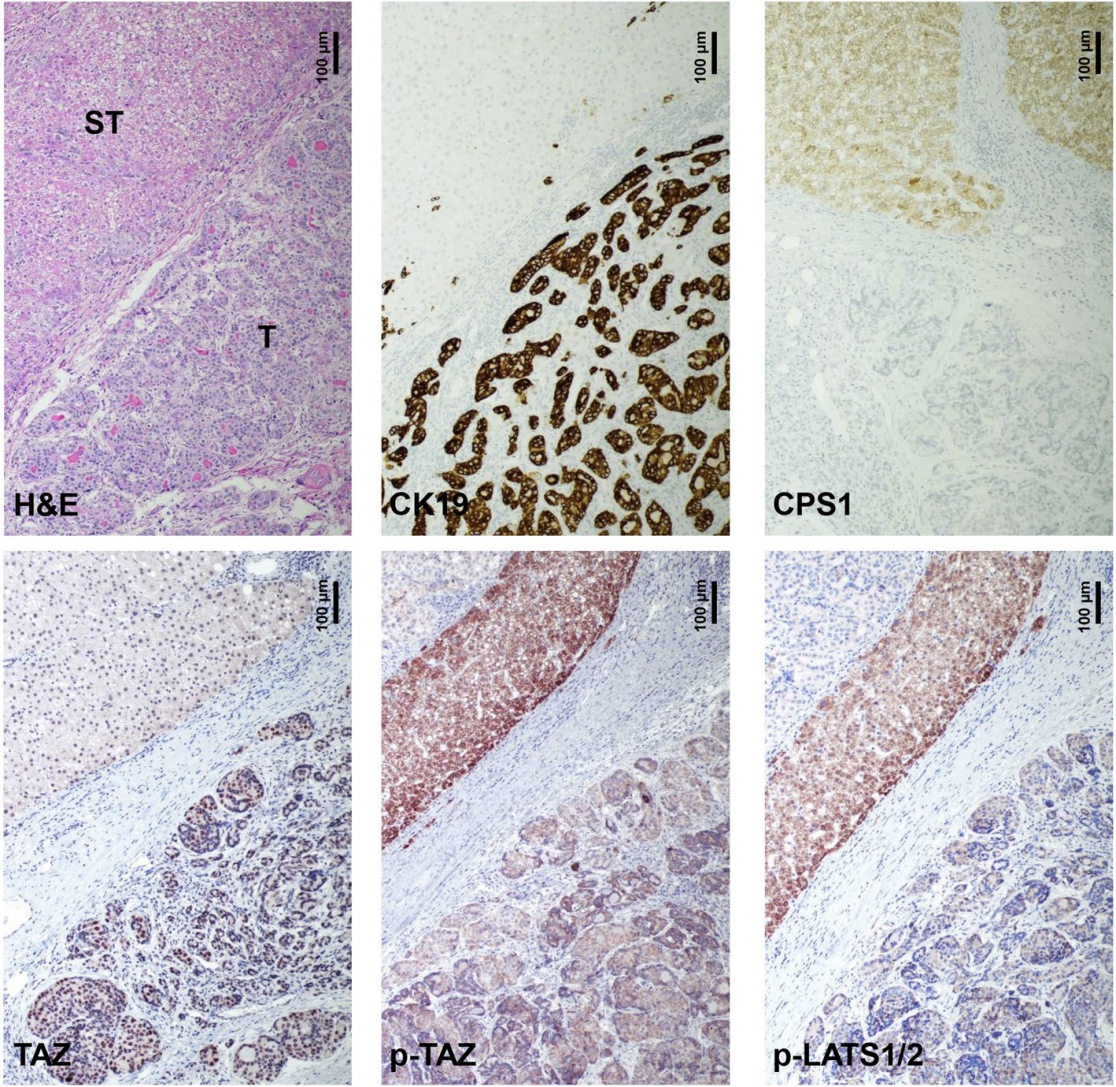
Representative immunohistochemical patterns of TAZ and its interactors in human intrahepatic cholangiocarcinomas. Pronounced nuclear staining for TAZ in a cholangiocellular tumor (T), which is positive to the biliary marker CK19 and negative to the hepatocellular marker CPS1, is paralleled by faint immunoreactivity for activated/phosphorylated (p-)LATS1/2 and inactivated/phosphorylated (p-)TAZ. Note the robust immunoreactivity for p-LATS1/2 and p-TAZ in the neighboring non-tumorous liver tissue (ST). Original magnification: 100x; scale bar: 100 μm. Abbreviations: H&E, hematoxylin and eosin staining.

Next, we assessed TAZ levels in human iCCA. We observed a significant upregulation of the *WWTR1* gene (encoding TAZ) levels in the tumor lesions vs. non-tumorous surrounding livers in an iCCA cohort for which clinicopathological and survival data were available (n=50; Supplementary Figure 9A). In these specimens, *WWTR1* expression did not correlate with the survival length of the patients, although a trend of lower survival in tumors with elevated *WWTR1* could be observed. Noticeably, a significant correlation between TAZ levels and the presence of lymph node metastases (*p* = 0.014) was detected. A similar correlation occurred between TAZ expression and lung metastases; however, it did not reach significance (p = 0.076), presumably due to the low number of cases with lung metastases. No significant correlations between *WWTR1* levels and clinicopathological data such as age, gender, liver cirrhosis, tumor grade, tumor stage, etc., were detected (Supplementary Material). Next, we analyzed the data on human cholangiocarcinoma specimens from The Cancer Genome Atlas (TCGA; http://ualcan.path.uab.edu/cgi-bin/TCGAExResultNew2.pl?genenam=WWTR1&ctype=CHOL). Similar to our collection's findings, the TCGA data indicate the lack of a correlation between *WWTR1* mRNA levels and patients' survival length. Notably, the TCGA data analysis revealed a significant correlation between *WWTR1* gene levels and lymph node infiltration by the tumor, in accordance with our data (Supplementary Figure 10). These concordant findings might suggest a role of WWTR1/TAZ in human iCCA dissemination and metastasis. No other clinicopathological data showed a significant correlation with *WWTR1* expression. Subsequently, we evaluated the TAZ protein levels in a vast collection of human iCCA specimens (n=182) by immunohistochemistry. Consistent with previous data [31, 32], robust nuclear immunoreactivity for TAZ was observed in almost all iCCA lesions (179/182, 98.3%) (Fig. 10). In contrast, TAZ cytoplasmic immunoreactivity characterized hepatocytes from normal livers and surrounding non-neoplastic livers, whereas nuclear TAZ accumulation occurred in cholangiocytes (Supplementary Figure 11). Notably, phosphorylated/inactivated TAZ (p-TAZ) levels were highest in the non-tumorous surrounding livers and normal cholangiocytes, indicating that TAZ is efficiently inactivated in the extra-tumoral areas (Supplementary Figure 11). Consistently, the immunoreactivity for activated/phosphorylated LATS1 and LATS2 proteins, which trigger the inactivation of TAZ, was more pronounced in the non-neoplastic surrounding counterparts, indicating that the control of TAZ degradation by LATS1/2 proteins is hampered along with iCCA development (Fig. 10). In addition, strong TAZ nuclear immunoreactivity was detected in the totality of preinvasive lesions (n=15, consisting of 9 intra-ductal papillary biliary neoplasms or IPBN and 6 biliary epithelial neoplasias or BilIN), implying that TAZ activation occurs before iCCA progression (Supplementary Figure 12). Furthermore, we revealed positive nuclear staining for TAZ in 9 of 9 (100%) mixed HCC/iCCA tumors (Supplementary Figure 13). In these tumors, TAZ nuclear immunoreactivity was present in the cholangiocellular and hepatocellular compartments, but it was always more pronounced in the cholangiocellular component. Finally, we evaluated the status of TAZ and interacting proteins in benign (non-neoplastic) conditions, namely ductular reactions (DR; Supplementary Figure 14). These are reactive processes that arise, in disease and injury, at the interface of the portal (or septal) and parenchymal compartments, in human livers [43, 44]. Significantly, DR showed a robust induction of TAZ, p-LATS1/2, and p-TAZ, implying that TAZ is activated in these lesions and counteracted/balanced by an efficient control system. Instead, the regulatory mechanisms are lost in the neighboring tumor lesions (Supplementary Figure 14).

Overall, the present data indicate that TAZ activation is a predominant event in human iCCA.

### Role of TAZ in iCCA *in vitro*

Next, we further assessed the effects of TAZ on the proliferation and survival of iCCA cell lines. For this purpose, TAZ was silenced either alone or in combination with its paralog YAP using specific small interfering RNAs in KKUM-213 and RBE cell lines (Supplementary Figure 15). In both cell lines, silencing of YAP triggered a compensatory upregulation of TAZ, whereas TAZ knockdown did not affect YAP expression. This finding suggests a compensatory mechanism inducing upregulation of TAZ when YAP is suppressed in iCCA cells. Similar data were previously obtained in hepatoblastoma cell lines [33]. Consistent with the latter hypothesis, simultaneous silencing of TAZ and YAP resulted in a significantly more pronounced reduction of cell proliferation than the silencing of either TAZ or YAP alone (Supplementary Figure 15). Furthermore, the combined knockdown of TAZ and YAP reduced the mRNA levels of Hippo targets (*CCN1* and *CCN2*) more profoundly than the single silencing of either gene (Supplementary Figure 16).

Thus, the current data indicate that TAZ promotes iCCA growth *in vitro* and suggest that TAZ and YAP might have overlapping and distinct functions in this process.

## Discussion

Human iCCA is characterized by insidious onset, clinical aggressiveness, and a lack of effective treatments [1–5]. Therefore, the molecular pathogenesis of iCCA should be deciphered to identify novel targets and establish more effective therapies.

Cumulating evidence supports the crucial role of the Hippo pathway in this disease. Specifically, the Hippo downstream effector YAP is aberrantly activated virtually in all iCCA cases [25, 31]. Furthermore, YAP cooperates with AKT and NOTCH1 to induce iCCA development in the mouse liver [37, 45]. However, the reports on the involvement of the YAP paralog TAZ in this disease are scanty. In the current study, we comprehensively investigated the role of TAZ in cholangiocarcinogenesis using human iCCA specimens, mouse models, and iCCA-derived cell lines. We found that TAZ is strongly and ubiquitously activated in this tumor type, starting from preinvasive lesions. In addition, general activation of TAZ was also detected in mixed HCC/iCCA, although the nuclear immunoreactivity was most pronounced in the cholangiocellular tumor compartment. The more robust immunoreactivity for TAZ in the cholangiocellular component might imply a predominant role in this compartment over the hepatocellular part. Also, it might explain why only pure iCCA were detected in mice injected with TAZ alone. Even in AKT/TAZ livers, the nuclear immunoreactivity for TAZ was more pronounced in the transfected cells with cholangiocellular traits cells over those with hepatocellular features. Importantly, SOX9 staining pattern recapitulates that of TAZ in the same liver lesions. Since SOX9, a Notch target, is a prominent driver in hepatocyte reprogramming into malignant cholangiocytes [39], the present findings suggest that the TAZ/SOX9 axis might drive the biliary differentiation of these tumors.

Of note, we revealed that TAZ activation also occurs in non-neoplastic conditions, such as DR lesions. However, while DR lesions display an intact LATS1/2 regulatory system of TAZ, presumably counterbalancing or fine-tuning its activity, this system is impaired in fully blown iCCA lesions. Thus, although additional studies are necessary to delineate this phenomenon in detail, the current study's findings support the pathogenetic relevance of the LATS1/2 disruption leading to TAZ unrestrained activity in cholangiocarcinogenesis. These conclusions are also supported by preliminary experiments conducted in our lab, showing that overexpression of the *TAZ* wild-type gene by hydrodynamic gene delivery, either alone or in combination with AKT, did not drive iCCA development in the mouse liver, different from the TAZS89A mutant form (Calvisi et al., unpublished observation). Therefore, the "simple" transcriptional upregulation of TAZ might not be sufficient to drive carcinogenesis due to LATS1/2 compensatory activation.

Regarding the TAZ tumorigenic potential *in vivo*, we discovered that overexpression of TAZ alone is weakly oncogenic in the mouse liver. Indeed, only a few tumors with pure cholangiocellular differentiation developed after a long latency in TAZ mice. The low malignant capacity of TAZ *in vivo* roughly mimicked the inability of YAP to trigger tumor development when overexpressed alone in the mouse liver via hydrodynamic injection [37]. In addition, similar to YAP, simultaneous upregulation of AKT in TAZ-overexpressing mouse livers induced rapid cholangiocarcinogenesis, suggesting the requirement of AKT for TAZ and YAP full oncogenic potential. According to this hypothesis, previous evidence from our laboratory indicates that TAZ can induce hepatocellular carcinoma and hepatoblastoma development in the mouse via hydrodynamic transfection only in association with other molecular events, such as overexpression of c-Myc, c-Met, and mutant β-catenin, or loss of APC [33, 46, 47]. The cellular and molecular mechanisms induced by collaborating oncogenes allowing TAZ to express its malignant properties remain unaddressed. Similar to that described for YAP, overexpression of TAZ induced the conversion of AKT-induced lipid-rich hepatocytes into fully malignant cholangiocytes. Significantly, a remarkable inflammatory response directed against malignant cholangiocytes, but not lipid-rich hepatocytes, occurs in the first weeks after hydrodynamic injection in AKT/TAZ livers, leading to the elimination of many of these cells via apoptosis. In this context, AKT might protect TAZ-overexpressing malignant cholangiocytes from cell death due to its potent anti-apoptotic properties. This hypothesis might also explain the extremely low rate of tumors in TAZ-injected mice, accompanied by the disappearance of TAZ-transfected cells from the hepatic parenchyma.

As observed in the case of its paralog [37], TAZ exerts its lineage commitment function via the canonical Notch pathway, as suppression of the latter abolished the tumors' cholangiocellular features. The present results expand the existing evidence, including murine CCA models generated by AKT/NICD [40], AKT/YAP [37], AKT/Jagged1 [34], and AKT/Fbxw7ΔF [48] mice, that the canonical Notch pathway is responsible for biliary commitment in mouse tumors induced by hydrodynamic gene delivery. Furthermore, a recent paper implies the Notch target SOX9 as the critical player downstream of YAP driving the transition from mature hepatocytes to biliary epithelial cells [39]. Similarly, a pronounced upregulation of SOX9 was detected in AKT/TAZ liver lesions. However, whether SOX9 is indispensable for cholangiocellular commitment in AKT/TAZ mice remains to be determined. Notably, inactivation of the Notch signaling suppressed the cholangiocellular phenotype of the lesions but did not significantly delay tumor development in AKT/TAZ mice. These findings confirm that TAZ possesses oncogenic features in the liver beyond the possibility of activating the Notch pathway. Of importance, the anti-growth effects of TAZ knockdown *in vivo* (mouse models) and *in vitro* (iCCA cell lines) were further augmented by YAP silencing. Specifically, the knockout of YAP led to tumor delay without affecting the histopathological features of AKT/TAZ tumors *in vivo*. Thus, the functions of TAZ and YAP might overlap only partly in cholangiocarcinogenesis, and the two oncogenes might exert a collaborative role in iCCA. Moreover, preliminary findings from our group show that simultaneous YAP and TAZ overexpression in the mouse liver does not drive tumor development (Chen X et al., unpublished observation). These data imply that YAP and TAZ are insufficient to trigger liver tumor initiation alone or in combination, at least by hydrodynamic gene delivery. Further studies using Taz and Yap conditional knockout mice will help to address the specific contribution and the distinct targets of the two protooncogenes in cholangiocarcinogenesis.

Although this body of evidence suggests that simultaneous TAZ and YAP inhibition might help restrain the growth of human iCCA, previous findings indicate that concomitant loss of the two genes severely impairs liver regeneration and paradoxically drives the development of hepatocellular adenomas [49, 50]. Thus, treatment approaches targeting TAZ and YAP should be cautiously evaluated in preclinical models before being applied to the clinical setting. Alternatively, therapeutic strategies against YAP and TAZ downstream effectors should be considered. For instance, it has been shown that iCCA displaying peculiar Notch signatures are likely to respond effectively to Notch inhibitors [51, 52]. Nonetheless, the present data indicate that Notch suppression should be coupled with other drugs to exert a robust anti-neoplastic potential. Indeed, Notch inhibition alone might modify the tumor phenotype without significantly affecting carcinogenesis. Furthermore, a better understanding of the functional crosstalk between the TAZ/YAP axis and other oncogenic pathways in iCCA is required to establish successful combinatorial therapies.

Another important observation of this study is that the lesions developed in AKT/TAZ mice closely recapitulate human intrahepatic cholangiocarcinoma's molecular features. Indeed, AKT/TAZ iCCAs are characterized by the acquisition of several markers of cholangiocellular differentiation, loss of hepatocellular markers, and gain of stemness/progenitor markers. Furthermore, a strong desmoplastic reaction surrounding the lesions parallels the carcinogenic process. Therefore, this preclinical model might be advantageous for deciphering the molecular pathogenesis of human iCCA and as a preclinical tool to test experimental therapeutics against this deadly disease.

## Conclusion

**Fig. 11:**
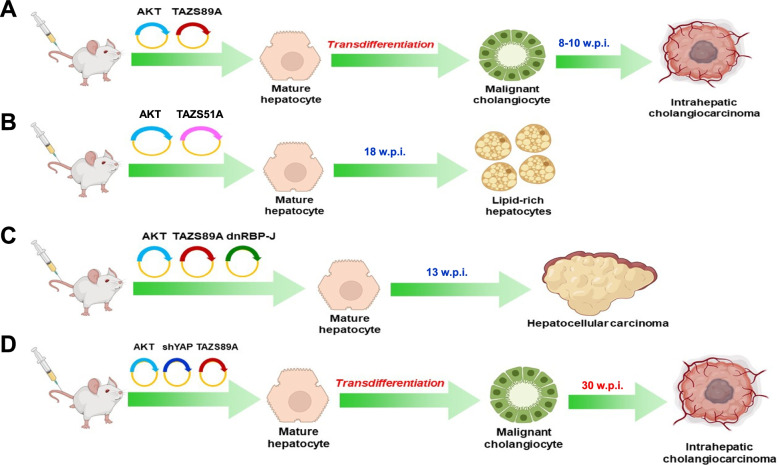
Summary of the oncogenic potential of TAZ in the mouse liver depending on its crosstalk with other genes. **(A)** Combined overexpression of myristoylated/activated AKT (AKT) and TAZS89A (a variant that escapes phosphorylation-mediated degradation by LATS1/2 proteins) drives the transdifferentiation of hepatocytes into malignant cholangiocytes, leading to intrahepatic cholangiocarcinoma development by 8-10 weeks post-injection. **(B)** Simultaneous overexpression of AKT and TAZS89AS51A (TAZS51A; a mutant form of TAZ that cannot bind to TEAD transcription factors) leads to the induction of clusters of lipid-rich and enlarged hepatocytes that are indistinguishable from those generated by transfection of AKT alone. **(C)** Overexpression of AKT and TAZS89A together with a dominant-negative form of the transcriptional regulator RBP-J (dnRBP-J) suppresses the NOTCH pathway, but does not impair AKT/TAZ-dependent liver carcinogenesis. Consequently, pure hepatocellular carcinoma lesions develop in AKT/TAZ/dnRBP-J mice by 13 weeks post-injection. **(D)** Suppression of YAP, the TAZ paralog, via short hairpin RNA delays cholangiocarcinogenesis in AKT/TAZ mice without affecting the tumor phenotype. Pure cholangiocarcinoma lesions form in AKT-shYAP-TAZ mice by 30 weeks post-injection. Abbreviations: w.p.i., weeks post-injection

In summary, we have shown that the TAZ protooncogene is widely activated in human iCCA and drives rapid cholangiocarcinogenesis in the mouse liver in association with AKT. The combination of TAZ with other genes, either activated or inactivated, results in distinct outcomes affecting tumor phenotype or iCCA progression (Fig. 11). Therapeutic strategies aimed at reactivating the mechanisms limiting TAZ hyperactivation and/or inhibiting TAZ/YAP oncogenic effects might be highly beneficial for treating human iCCA.

## Supplementary Information

**Additional file 1.**

**Additional file 2.**

**Additional file 3.**

**Additional file 4.**

**Additional file 5.**

**Additional file 6.**

**Additional file 7.**

**Additional file 8.**

**Additional file 9.**

**Additional file 10.**

**Additional file 11.**

**Additional file 12.**

**Additional file 13.**

**Additional file 14.**

**Additional file 15.**

**Additional file 16.**

**Additional file 17.**

## Data Availability

All data generated or analyzed during this study are included in this published article [and its supplementary information files].

## Abbreviations

α-SMA: alpha-smooth muscle actin
ARG1: liver arginase
BilIN: biliary epithelial neoplasias
CEBPA: CCAAT enhancer-binding protein alpha
CK: cytokeratin
CPS1 or Hep Par-19: carbamoyl-phosphate synthase 1
CYP2E1: cytochrome P450 2E1
CYP3A4: cytochrome P450 3A4
FOXA1: forkhead box A1
FOXA2: forkhead box A2
S100A4: S100 calcium-binding protein A4
GLUL: glutamine synthetase
HB: Hepatoblastoma
HCC: Hepatocellular carcinoma
HNF4α: hepatocyte nuclear factor 4 alpha
iCCA: Intrahepatic Cholangiocarcinoma
IPBN: intra-ductal papillary biliary neoplasms
NF2: neurofibromatosis type 2
PDGFRβ: platelet-derived growth factor receptor beta
p-GSK-3β: phosphorylated glycogen synthase kinase 3 beta
p-RPS6: phosphorylated ribosomal protein S6
qRT-PCR: quantitative real-time polymerase chain reaction
SEER: Surveillance, Epidemiology, and End Results
siRNA: small interfering RNA
TAZ: transcriptional coactivator with PDZ-binding motif
Tbg: thyroxine-binding globulin
YAP: Yes-associated protein
