# Correction: The Hippo pathway efector TAZ induces intrahepatic cholangiocarcinoma in mice and is ubiquitously activated in the human disease

**DOI:** 10.1186/s13046-023-02709-x

**Published:** 2023-05-17

**Authors:** Antonio Cigliano, Shanshan Zhang, Silvia Ribback, Sara Steinmann, Marcella Sini, Cindy E. Ament, Kirsten Utpatel, Xinhua Song, Jingxiao Wang, Maria G. Pilo, Fabian Berger, Haichuan Wang, Junyan Tao, Xiaolei Li, Giovanni M. Pes, Serena Mancarella, Gianluigi Giannelli, Frank Dombrowski, Matthias Evert, Diego F. Calvisi, Xin Chen, Katja Evert

**Affiliations:** 1 Institute of Pathology, University of Regensburg, Franz-Josef-Strauß-Allee 11, Regensburg, Germany; 2 Department of Medical, Surgical and Experimental Sciences, University of Sassari, Sassari, Italy; 3 Department of Bioengineering and Therapeutic Sciences and Liver Center, University of California, 513 Parnassus Avenue, San Francisco, CA USA; 4 Department of Pathology, Eastern Hepatobiliary Surgery Hospital, Second Military Medical University, Shanghai, China; 5 Institute of Pathology, University of Greifswald, Greifswald, Germany; 6 Experimental Pathology Unit, Department of Biomedical Sciences, University of Cagliari, Cagliari, Italy; 7 School of Traditional Chinese Medicine, Capital Medical University, Beijing, China; 8 School of Life Sciences, Beijing University of Chinese Medicine, Beijing, China; 9 Liver Transplantation Division, Department of Liver Surgery, West China Hospital, Sichuan University, Chengdu, China; 10 Department of Thyroid and Breast Surgery, The 960Th Hospital of the PLA, Jinan, 250031 China; 11 National Institute of Gastroenterology, IRCCS- Saverio de Bellis Research Hospital, Castellana Grotte, Italy; 12 University of Hawaii Cancer Center, Honolulu, HI USA

**Correction:** ***J Exp Clin Cancer Res*** **41, 92 (2022)**

**https://doi.org/10.1186/s13046-022-02394-2**

Following publication of the original article [1], an error was identified in the corresponding author’s affiliation.

The updated affiliation is given below and the changes have been highlighted in **bold typeface**.

^11^ National Institute of Gastroenterology, **IRCCS- Saverio de Bellis** Research Hospital, Castellana Grotte, Italy.

The correction do not affect the overall Conclusion of the article.
