# Supplementary material for: The Hippo pathway effector TAZ induces intrahepatic cholangiocarcinoma in mice and is ubiquitously activated in the human disease
Source: J Exp Clin Cancer Res. 2022 Jun 3;41:192. doi: 10.1186/s13046-022-02394-2 (PMC9164528; doi:10.1186/s13046-022-02394-2)
Supplement: Supplementary file 1 — Additional file 1. [file 13046_2022_2394_MOESM1_ESM.pptx]

## Slide 1
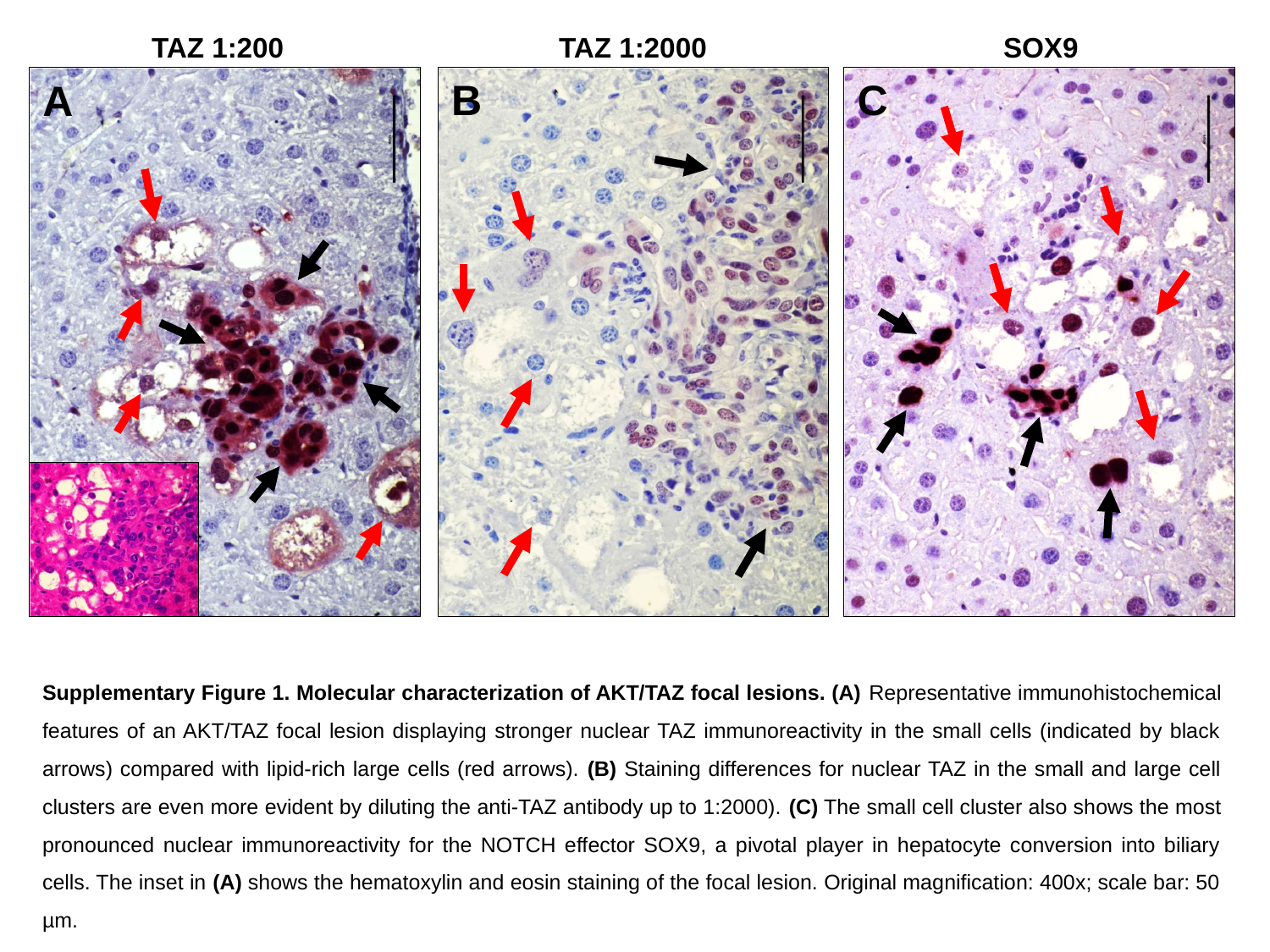

TAZ 1:200
TAZ 1:2000
SOX9
C
B
A
Supplementary Figure 1. Molecular characterization of AKT/TAZ focal lesions. (A) Representative immunohistochemical features of an AKT/TAZ focal lesion displaying stronger nuclear TAZ immunoreactivity in the small cells (indicated by black arrows) compared with lipid-rich large cells (red arrows). (B) Staining differences for nuclear TAZ in the small and large cell clusters are even more evident by diluting the anti-TAZ antibody up to 1:2000). (C) The small cell cluster also shows the most pronounced nuclear immunoreactivity for the NOTCH effector SOX9, a pivotal player in hepatocyte conversion into biliary cells. The inset in (A) shows the hematoxylin and eosin staining of the focal lesion. Original magnification: 400x; scale bar: 50 µm.
