# Supplementary material for: The Hippo pathway effector TAZ induces intrahepatic cholangiocarcinoma in mice and is ubiquitously activated in the human disease
Source: J Exp Clin Cancer Res. 2022 Jun 3;41:192. doi: 10.1186/s13046-022-02394-2 (PMC9164528; doi:10.1186/s13046-022-02394-2)
Supplement: Supplementary file 2 — Additional file 2. [file 13046_2022_2394_MOESM2_ESM.pptx]

## Slide 1
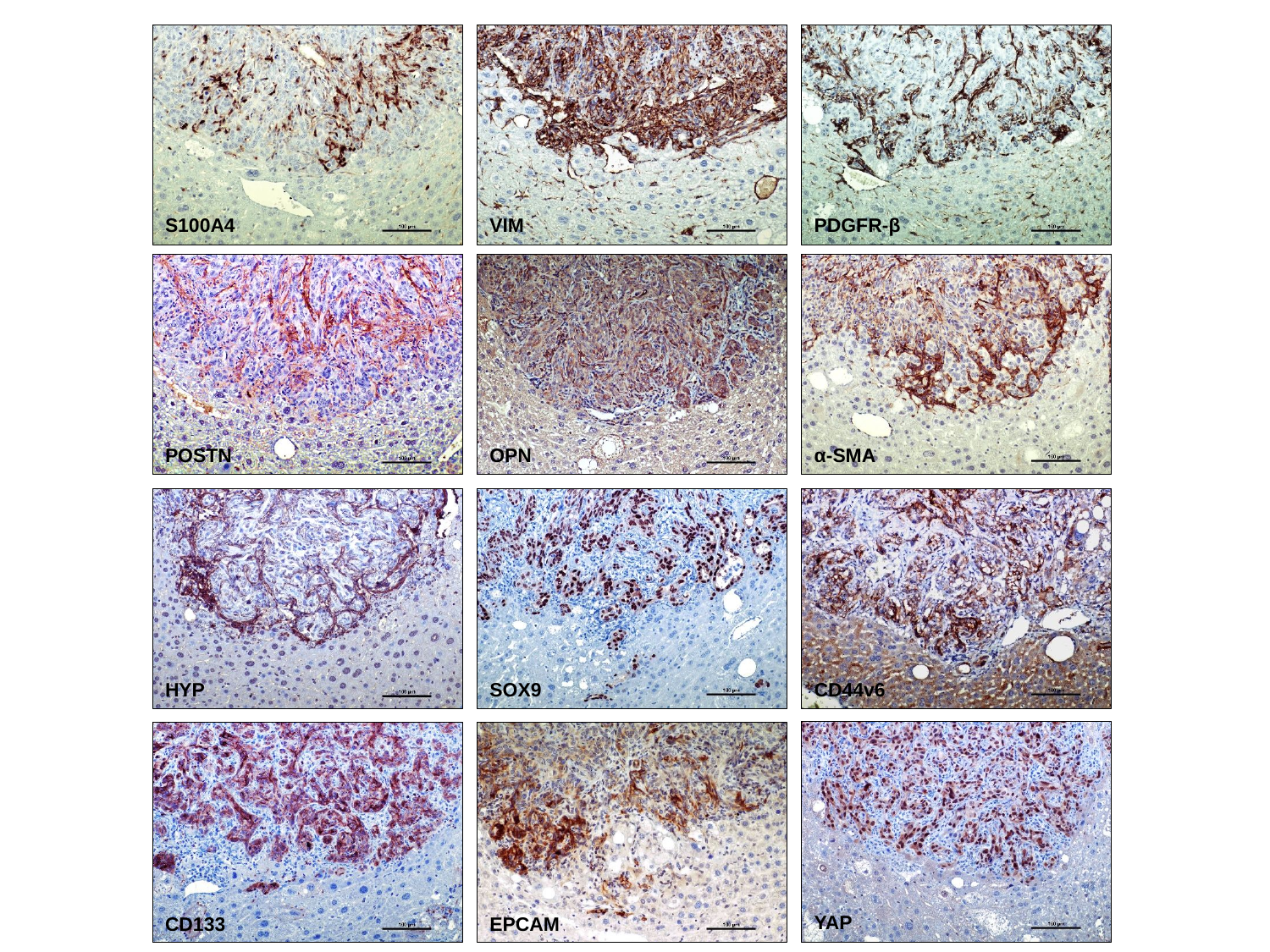

S100A4
VIM
PDGFR-β
POSTN
OPN
α-SMA
HYP
SOX9
CD44v6
YAP
CD133
EPCAM

## Slide 2
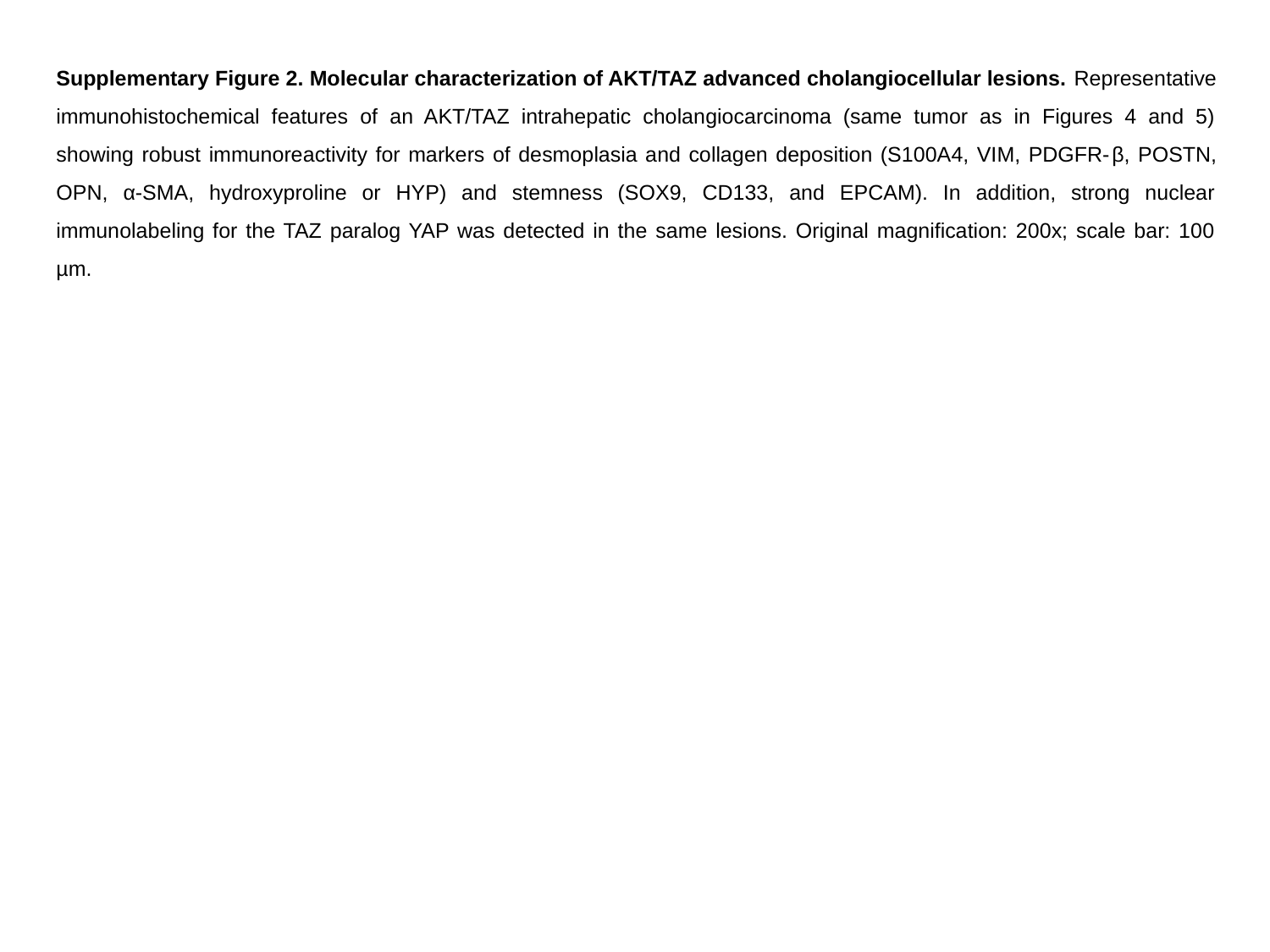

Supplementary Figure 2. Molecular characterization of AKT/TAZ advanced cholangiocellular lesions. Representative immunohistochemical features of an AKT/TAZ intrahepatic cholangiocarcinoma (same tumor as in Figures 4 and 5) showing robust immunoreactivity for markers of desmoplasia and collagen deposition (S100A4, VIM, PDGFR-β, POSTN, OPN, α-SMA, hydroxyproline or HYP) and stemness (SOX9, CD133, and EPCAM). In addition, strong nuclear immunolabeling for the TAZ paralog YAP was detected in the same lesions. Original magnification: 200x; scale bar: 100 µm.
