# Supplementary material for: The Hippo pathway effector TAZ induces intrahepatic cholangiocarcinoma in mice and is ubiquitously activated in the human disease
Source: J Exp Clin Cancer Res. 2022 Jun 3;41:192. doi: 10.1186/s13046-022-02394-2 (PMC9164528; doi:10.1186/s13046-022-02394-2)
Supplement: Supplementary file 3 — Additional file 3. [file 13046_2022_2394_MOESM3_ESM.pptx]

## Slide 1
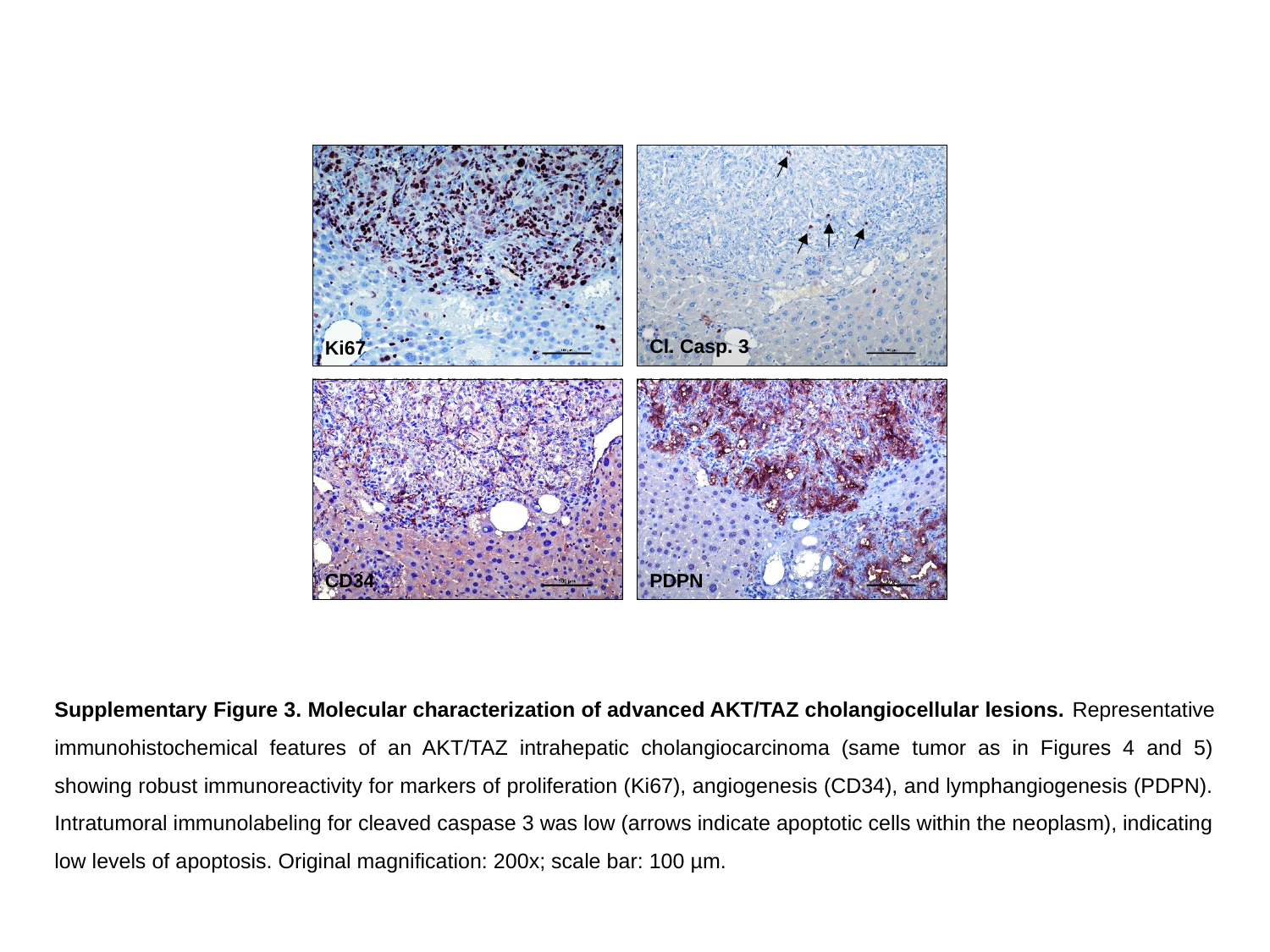

Cl. Casp. 3
Ki67
CD34
PDPN
Supplementary Figure 3. Molecular characterization of advanced AKT/TAZ cholangiocellular lesions. Representative immunohistochemical features of an AKT/TAZ intrahepatic cholangiocarcinoma (same tumor as in Figures 4 and 5) showing robust immunoreactivity for markers of proliferation (Ki67), angiogenesis (CD34), and lymphangiogenesis (PDPN). Intratumoral immunolabeling for cleaved caspase 3 was low (arrows indicate apoptotic cells within the neoplasm), indicating low levels of apoptosis. Original magnification: 200x; scale bar: 100 µm.
