# Supplementary material for: The Hippo pathway effector TAZ induces intrahepatic cholangiocarcinoma in mice and is ubiquitously activated in the human disease
Source: J Exp Clin Cancer Res. 2022 Jun 3;41:192. doi: 10.1186/s13046-022-02394-2 (PMC9164528; doi:10.1186/s13046-022-02394-2)
Supplement: Supplementary file 4 — Additional file 4. [file 13046_2022_2394_MOESM4_ESM.pptx]

## Slide 1
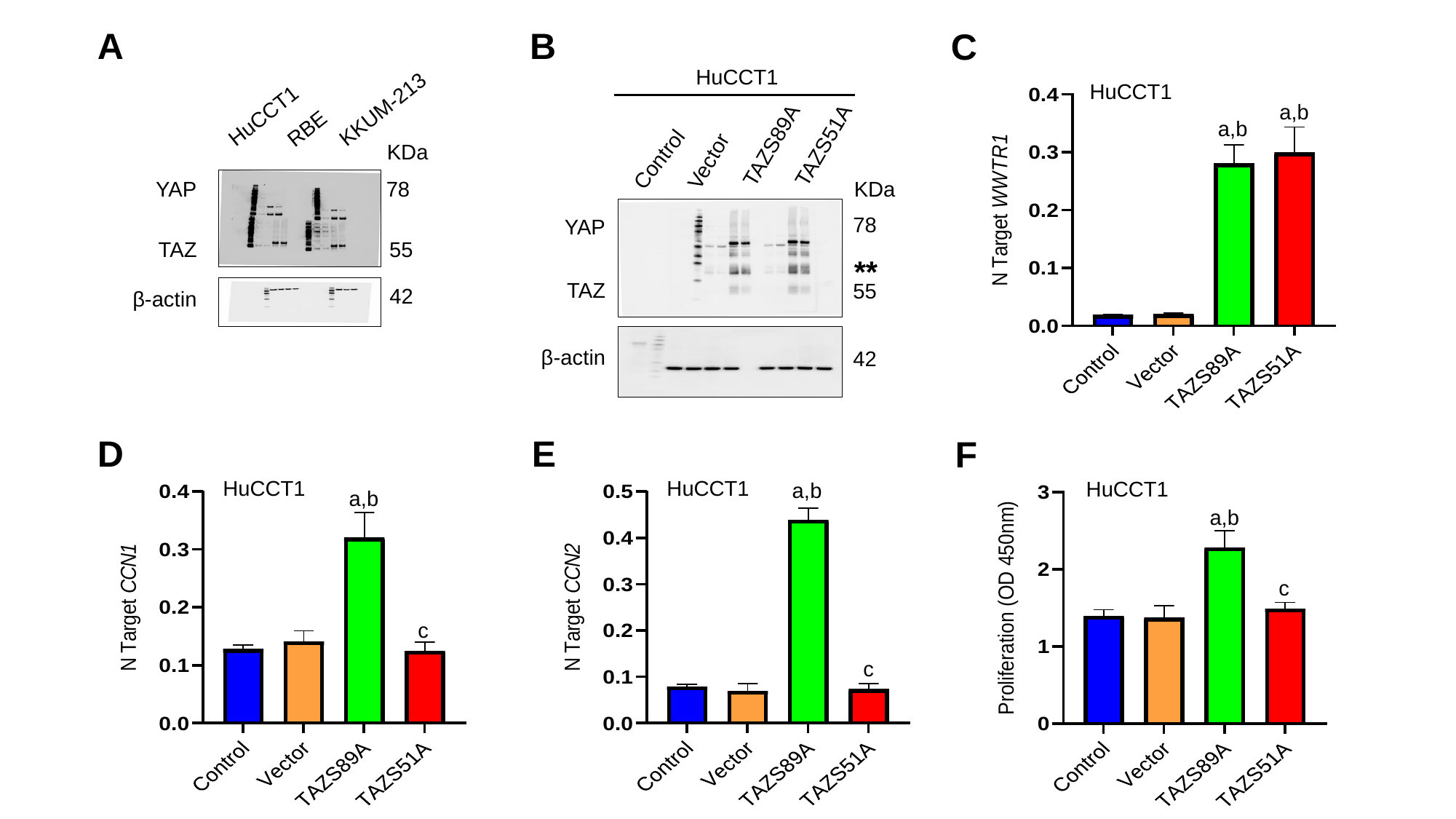

B
A
C
HuCCT1
HuCCT1
KKUM-213
a,b
HuCCT1
RBE
a,b
TAZS51A
TAZS89A
KDa
Control
Vector
KDa
YAP
78
78
YAP
TAZ
55
**
TAZ
55
42
β-actin
β-actin
42
D
E
F
HuCCT1
HuCCT1
HuCCT1
a,b
a,b
a,b
c
c
c

## Slide 2
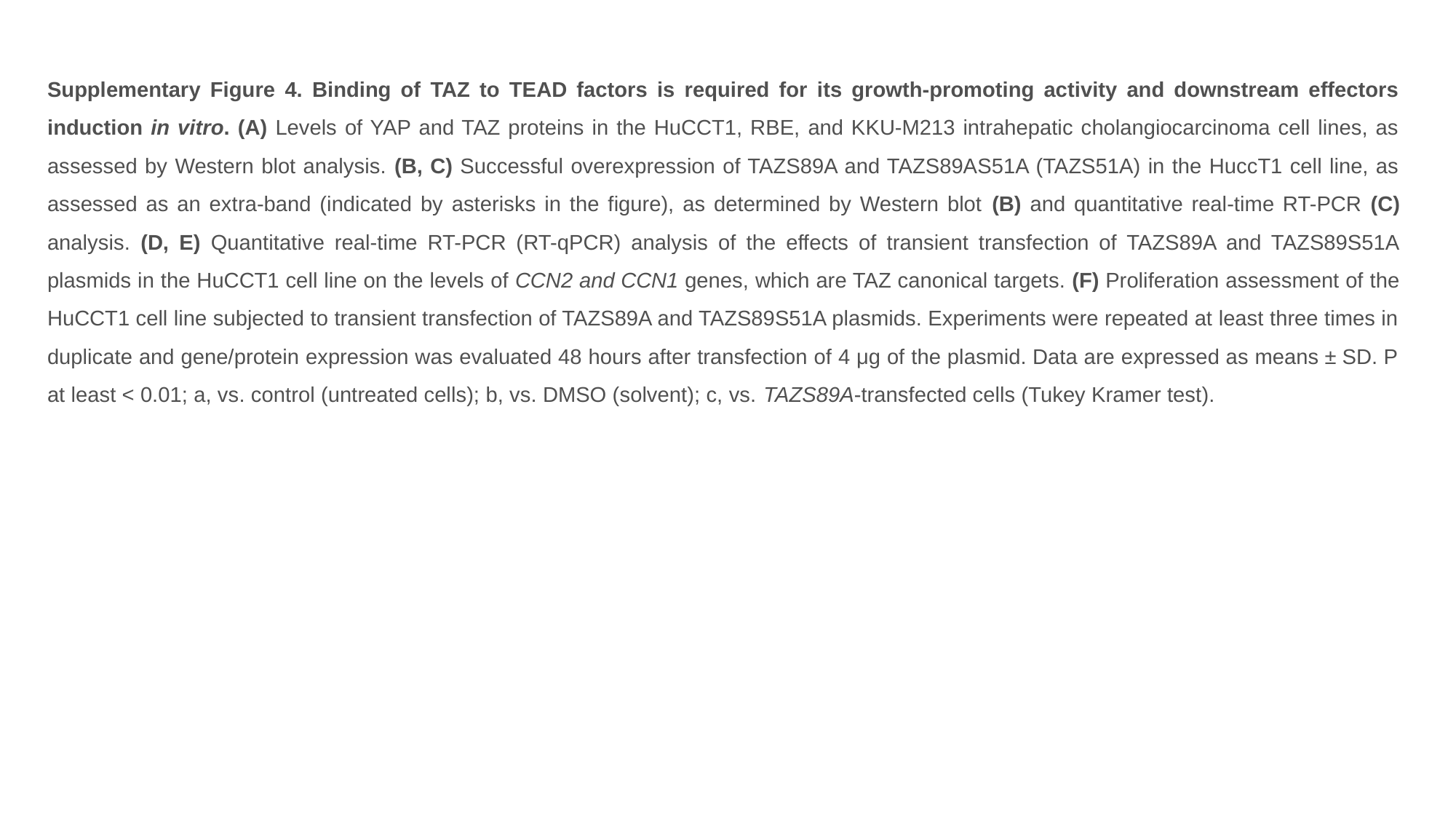

Supplementary Figure 4. Binding of TAZ to TEAD factors is required for its growth-promoting activity and downstream effectors induction in vitro. (A) Levels of YAP and TAZ proteins in the HuCCT1, RBE, and KKU-M213 intrahepatic cholangiocarcinoma cell lines, as assessed by Western blot analysis. (B, C) Successful overexpression of TAZS89A and TAZS89AS51A (TAZS51A) in the HuccT1 cell line, as assessed as an extra-band (indicated by asterisks in the figure), as determined by Western blot (B) and quantitative real-time RT-PCR (C) analysis. (D, E) Quantitative real-time RT-PCR (RT-qPCR) analysis of the effects of transient transfection of TAZS89A and TAZS89S51A plasmids in the HuCCT1 cell line on the levels of CCN2 and CCN1 genes, which are TAZ canonical targets. (F) Proliferation assessment of the HuCCT1 cell line subjected to transient transfection of TAZS89A and TAZS89S51A plasmids. Experiments were repeated at least three times in duplicate and gene/protein expression was evaluated 48 hours after transfection of 4 μg of the plasmid. Data are expressed as means ± SD. P at least < 0.01; a, vs. control (untreated cells); b, vs. DMSO (solvent); c, vs. TAZS89A-transfected cells (Tukey Kramer test).
