# Supplementary material for: The Hippo pathway effector TAZ induces intrahepatic cholangiocarcinoma in mice and is ubiquitously activated in the human disease
Source: J Exp Clin Cancer Res. 2022 Jun 3;41:192. doi: 10.1186/s13046-022-02394-2 (PMC9164528; doi:10.1186/s13046-022-02394-2)
Supplement: Supplementary file 5 — Additional file 5. [file 13046_2022_2394_MOESM5_ESM.pptx]

## Slide 1
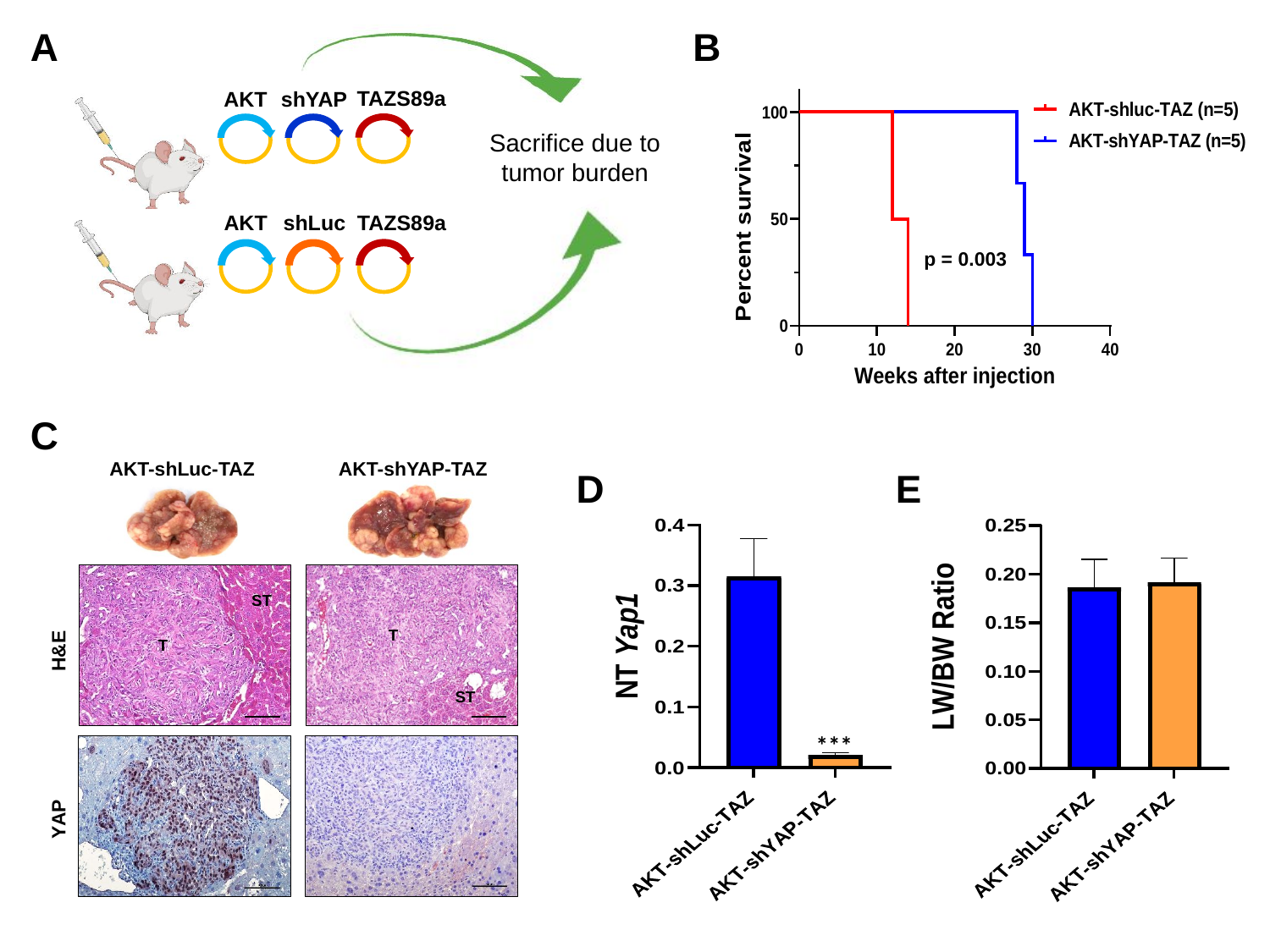

A
B
TAZS89a
AKT
shYAP
Sacrifice due to
tumor burden
TAZS89a
AKT
shLuc
p = 0.003
C
AKT-shLuc-TAZ
AKT-shYAP-TAZ
D
E
ST
T
T
H&E
ST
***
YAP

## Slide 2
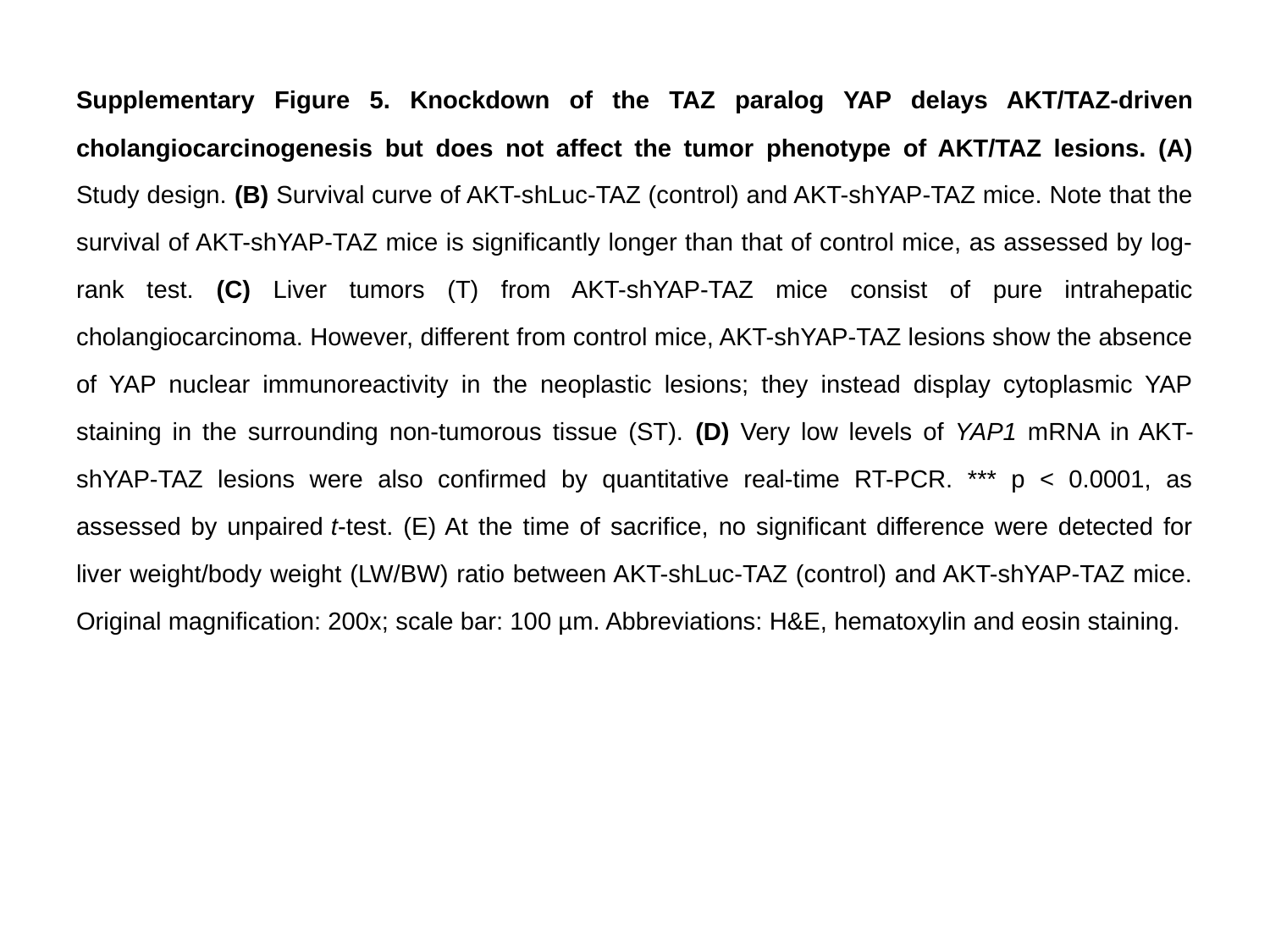

Supplementary Figure 5. Knockdown of the TAZ paralog YAP delays AKT/TAZ-driven cholangiocarcinogenesis but does not affect the tumor phenotype of AKT/TAZ lesions. (A) Study design. (B) Survival curve of AKT-shLuc-TAZ (control) and AKT-shYAP-TAZ mice. Note that the survival of AKT-shYAP-TAZ mice is significantly longer than that of control mice, as assessed by log-rank test. (C) Liver tumors (T) from AKT-shYAP-TAZ mice consist of pure intrahepatic cholangiocarcinoma. However, different from control mice, AKT-shYAP-TAZ lesions show the absence of YAP nuclear immunoreactivity in the neoplastic lesions; they instead display cytoplasmic YAP staining in the surrounding non-tumorous tissue (ST). (D) Very low levels of YAP1 mRNA in AKT-shYAP-TAZ lesions were also confirmed by quantitative real-time RT-PCR. *** p < 0.0001, as assessed by unpaired t-test. (E) At the time of sacrifice, no significant difference were detected for liver weight/body weight (LW/BW) ratio between AKT-shLuc-TAZ (control) and AKT-shYAP-TAZ mice. Original magnification: 200x; scale bar: 100 µm. Abbreviations: H&E, hematoxylin and eosin staining.
