# Supplementary material for: The Hippo pathway effector TAZ induces intrahepatic cholangiocarcinoma in mice and is ubiquitously activated in the human disease
Source: J Exp Clin Cancer Res. 2022 Jun 3;41:192. doi: 10.1186/s13046-022-02394-2 (PMC9164528; doi:10.1186/s13046-022-02394-2)
Supplement: Supplementary file 6 — Additional file 6. [file 13046_2022_2394_MOESM6_ESM.pptx]

## Slide 1
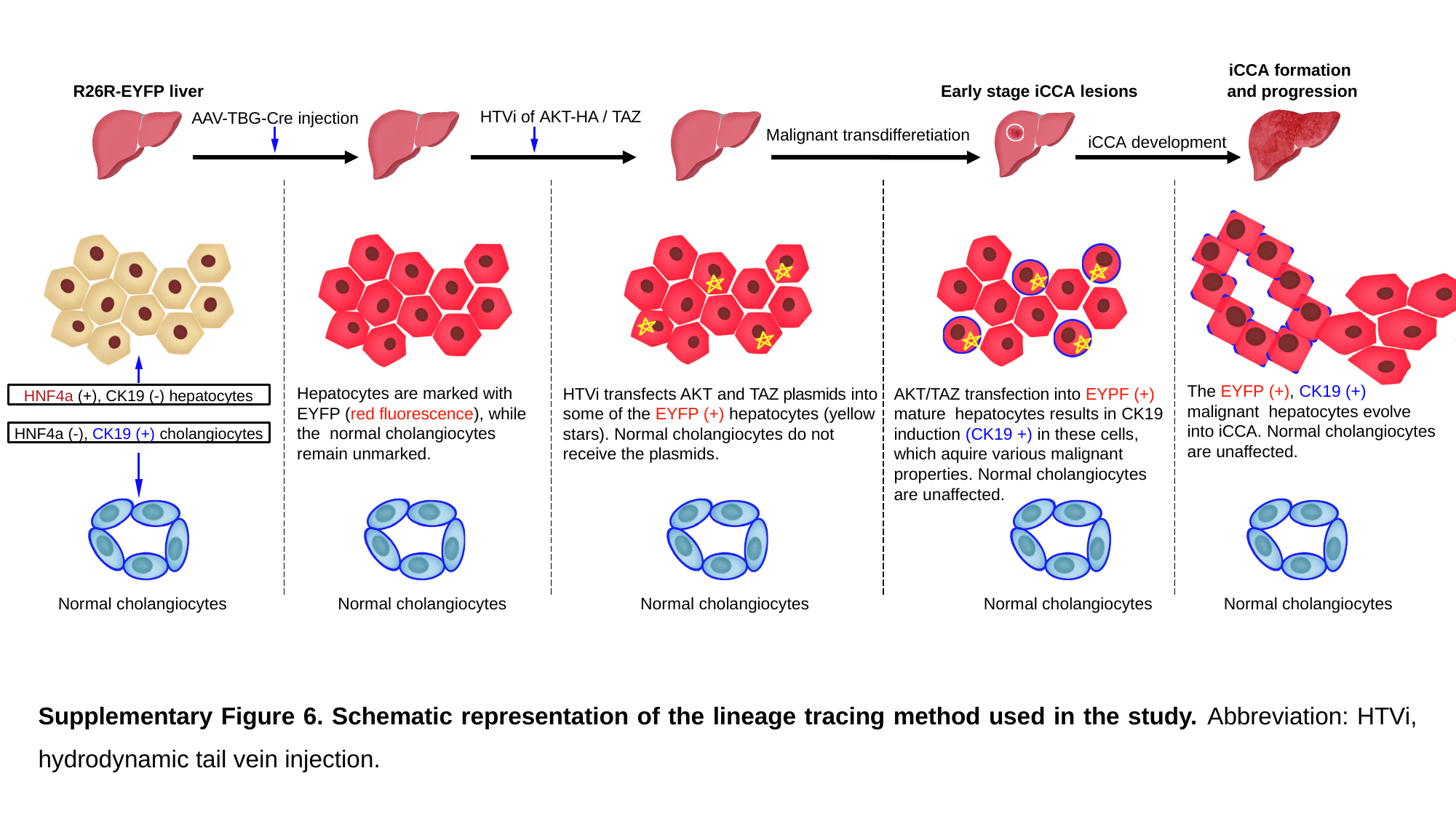

iCCA formation
and progression
R26R-EYFP liver
AAV-TBG-Cre injection
Early stage iCCA lesions
HTVi of AKT-HA / TAZ
Malignant transdifferetiation
iCCA development
The EYFP (+), CK19 (+) malignant hepatocytes evolve into iCCA. Normal cholangiocytes are unaffected.
Hepatocytes are marked with EYFP (red fluorescence), while the normal cholangiocytes remain unmarked.
HTVi transfects AKT and TAZ plasmids into some of the EYFP (+) hepatocytes (yellow stars). Normal cholangiocytes do not receive the plasmids.
AKT/TAZ transfection into EYPF (+) mature hepatocytes results in CK19 induction (CK19 +) in these cells, which aquire various malignant properties. Normal cholangiocytes
are unaffected.
HNF4a (+), CK19 (-) hepatocytes
HNF4a (-), CK19 (+) cholangiocytes
Normal cholangiocytes
Normal cholangiocytes
Normal cholangiocytes
Normal cholangiocytes
Normal cholangiocytes
Supplementary Figure 6. Schematic representation of the lineage tracing method used in the study. Abbreviation: HTVi, hydrodynamic tail vein injection.
