# Supplementary material for: The Hippo pathway effector TAZ induces intrahepatic cholangiocarcinoma in mice and is ubiquitously activated in the human disease
Source: J Exp Clin Cancer Res. 2022 Jun 3;41:192. doi: 10.1186/s13046-022-02394-2 (PMC9164528; doi:10.1186/s13046-022-02394-2)
Supplement: Supplementary file 7 — Additional file 7. [file 13046_2022_2394_MOESM7_ESM.pptx]

## Slide 1
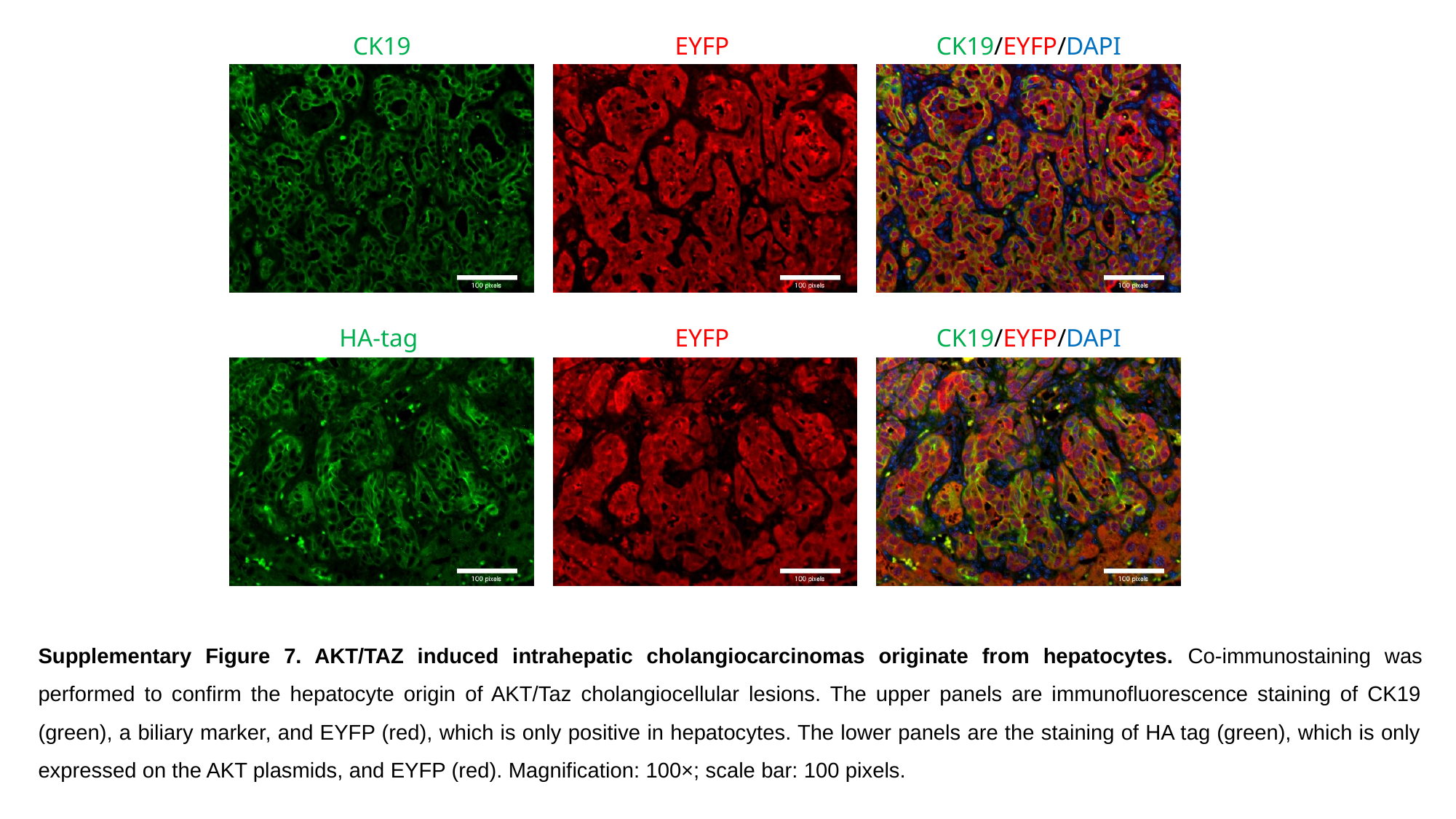

CK19
EYFP
CK19/EYFP/DAPI
HA-tag
EYFP
CK19/EYFP/DAPI
Supplementary Figure 7. AKT/TAZ induced intrahepatic cholangiocarcinomas originate from hepatocytes. Co-immunostaining was performed to confirm the hepatocyte origin of AKT/Taz cholangiocellular lesions. The upper panels are immunofluorescence staining of CK19 (green), a biliary marker, and EYFP (red), which is only positive in hepatocytes. The lower panels are the staining of HA tag (green), which is only expressed on the AKT plasmids, and EYFP (red). Magnification: 100×; scale bar: 100 pixels.
