# Supplementary material for: The Hippo pathway effector TAZ induces intrahepatic cholangiocarcinoma in mice and is ubiquitously activated in the human disease
Source: J Exp Clin Cancer Res. 2022 Jun 3;41:192. doi: 10.1186/s13046-022-02394-2 (PMC9164528; doi:10.1186/s13046-022-02394-2)
Supplement: Supplementary file 8 — Additional file 8. [file 13046_2022_2394_MOESM8_ESM.pptx]

## Slide 1
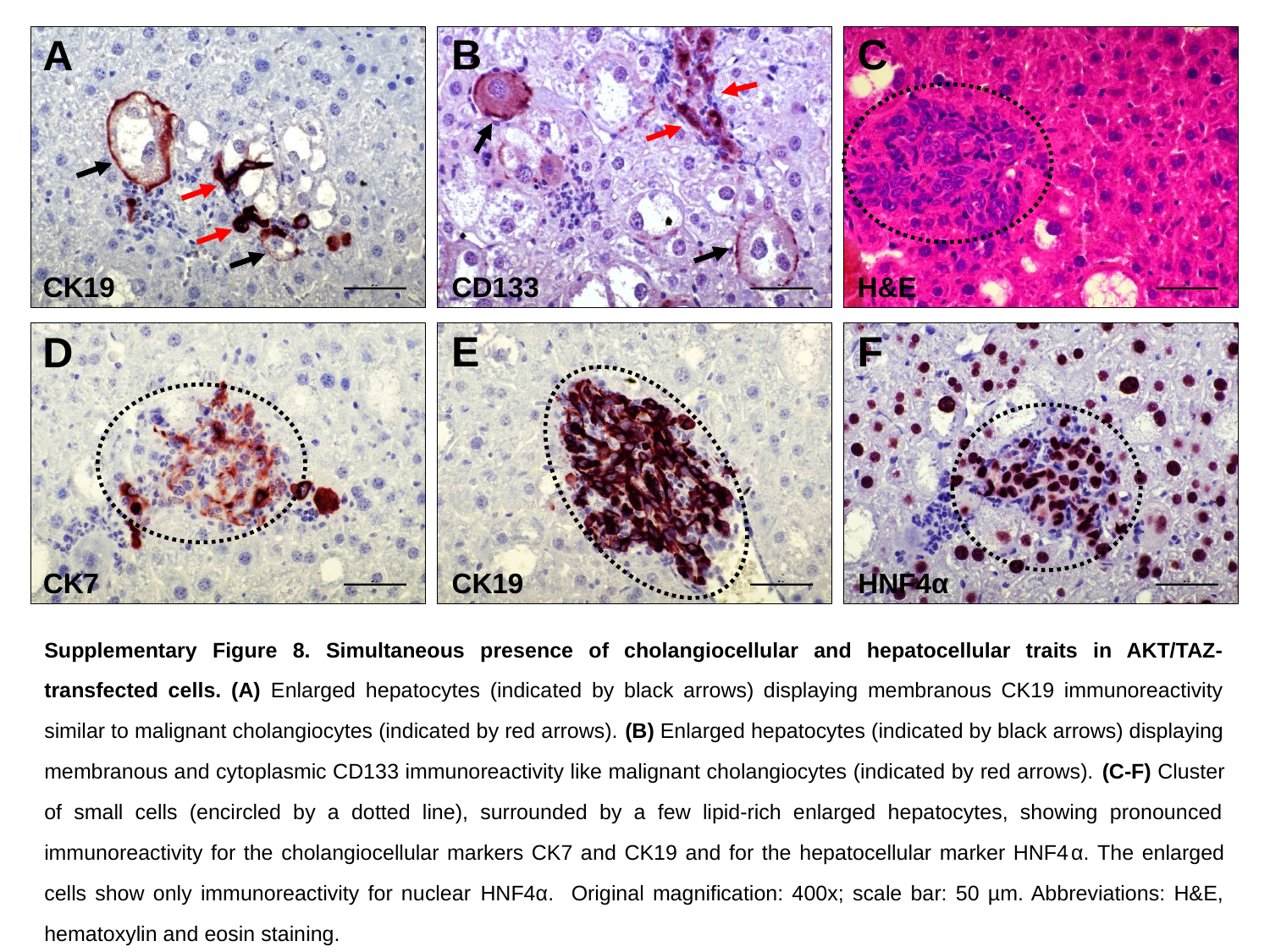

C
B
A
CK19
CD133
H&E
F
E
D
CK7
CK19
HNF4α
Supplementary Figure 8. Simultaneous presence of cholangiocellular and hepatocellular traits in AKT/TAZ-transfected cells. (A) Enlarged hepatocytes (indicated by black arrows) displaying membranous CK19 immunoreactivity similar to malignant cholangiocytes (indicated by red arrows). (B) Enlarged hepatocytes (indicated by black arrows) displaying membranous and cytoplasmic CD133 immunoreactivity like malignant cholangiocytes (indicated by red arrows). (C-F) Cluster of small cells (encircled by a dotted line), surrounded by a few lipid-rich enlarged hepatocytes, showing pronounced immunoreactivity for the cholangiocellular markers CK7 and CK19 and for the hepatocellular marker HNF4α. The enlarged cells show only immunoreactivity for nuclear HNF4α. Original magnification: 400x; scale bar: 50 µm. Abbreviations: H&E, hematoxylin and eosin staining.
