# Supplementary material for: The Hippo pathway effector TAZ induces intrahepatic cholangiocarcinoma in mice and is ubiquitously activated in the human disease
Source: J Exp Clin Cancer Res. 2022 Jun 3;41:192. doi: 10.1186/s13046-022-02394-2 (PMC9164528; doi:10.1186/s13046-022-02394-2)
Supplement: Supplementary file 9 — Additional file 9. [file 13046_2022_2394_MOESM9_ESM.pptx]

## Slide 1
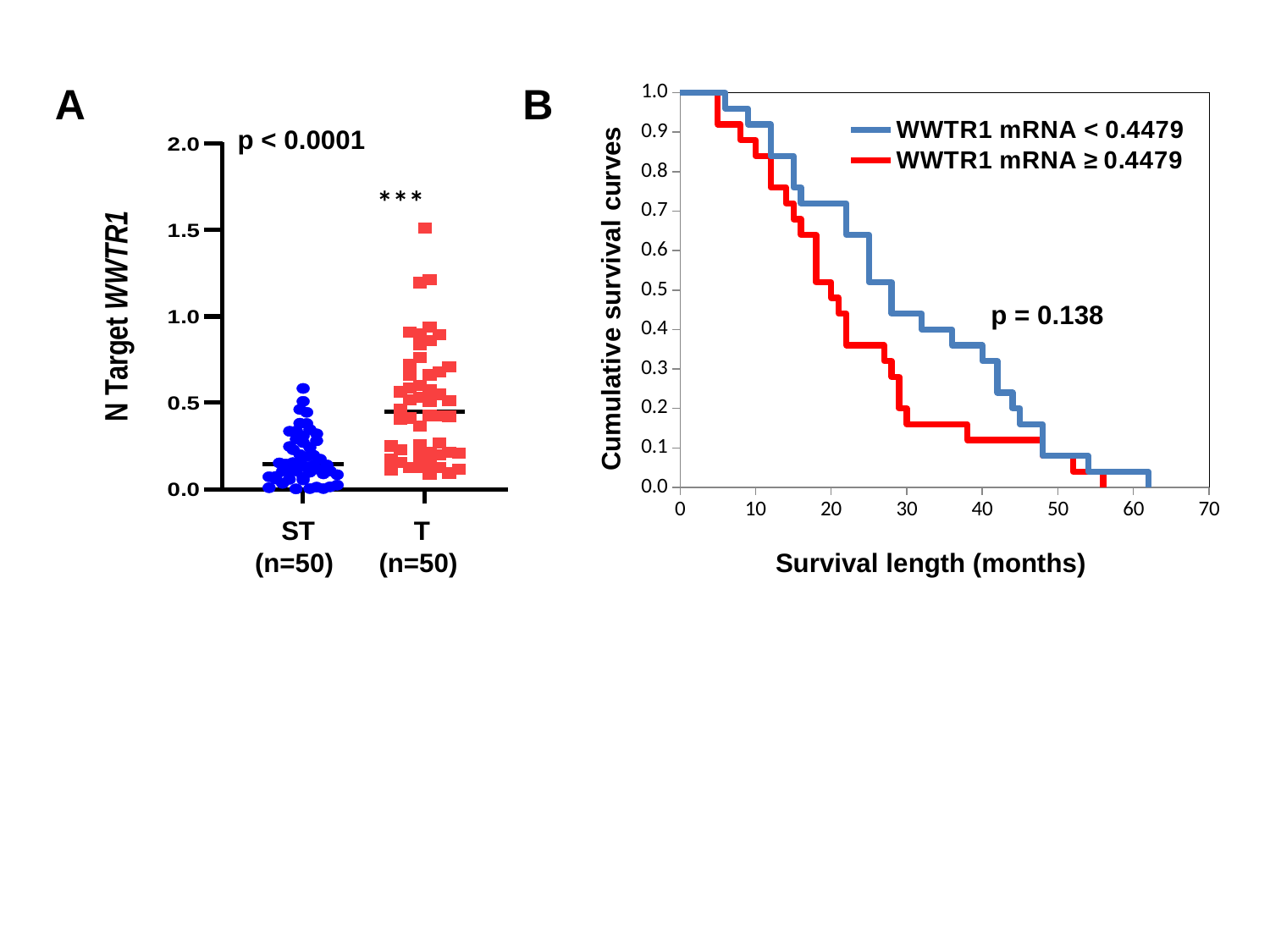

### Chart
| Category | | |
|---|---|---|A
B
p < 0.0001
***
p = 0.138
ST
(n=50)
T
(n=50)
Survival length (months)

## Slide 2
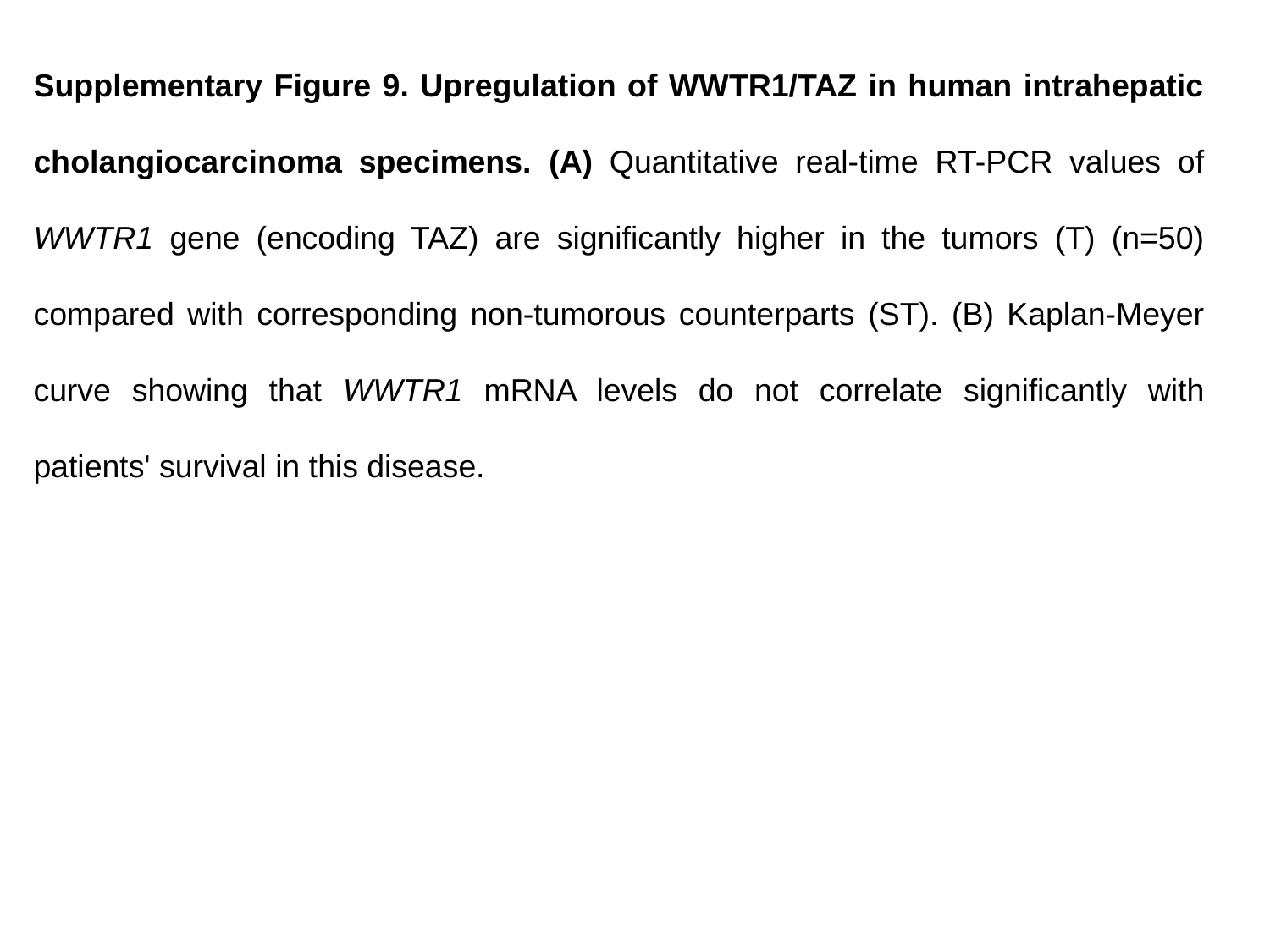

Supplementary Figure 9. Upregulation of WWTR1/TAZ in human intrahepatic cholangiocarcinoma specimens. (A) Quantitative real-time RT-PCR values of WWTR1 gene (encoding TAZ) are significantly higher in the tumors (T) (n=50) compared with corresponding non-tumorous counterparts (ST). (B) Kaplan-Meyer curve showing that WWTR1 mRNA levels do not correlate significantly with patients' survival in this disease.
