# Supplementary material for: The Hippo pathway effector TAZ induces intrahepatic cholangiocarcinoma in mice and is ubiquitously activated in the human disease
Source: J Exp Clin Cancer Res. 2022 Jun 3;41:192. doi: 10.1186/s13046-022-02394-2 (PMC9164528; doi:10.1186/s13046-022-02394-2)
Supplement: Supplementary file 10 — Additional file 10. [file 13046_2022_2394_MOESM10_ESM.pptx]

## Slide 1
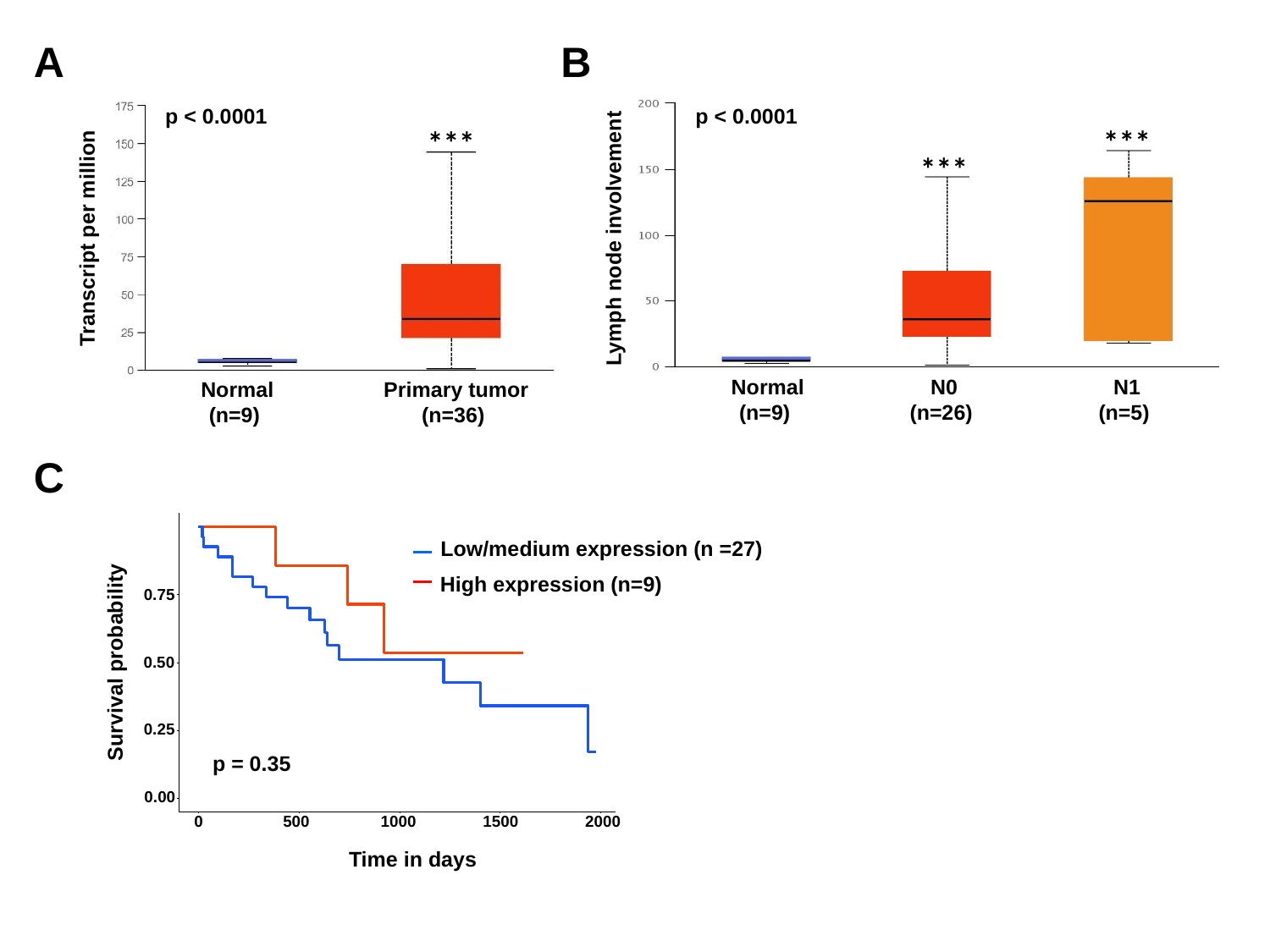

A
B
p < 0.0001
p < 0.0001
***
***
***
Transcript per million
Lymph node involvement
Normal
(n=9)
N0
(n=26)
N1
(n=5)
Normal
(n=9)
Primary tumor
(n=36)
C
Low/medium expression (n =27)
High expression (n=9)
0.75
Survival probability
0.50
0.25
p = 0.35
0.00
0 500 1000 1500 2000
Time in days

## Slide 2
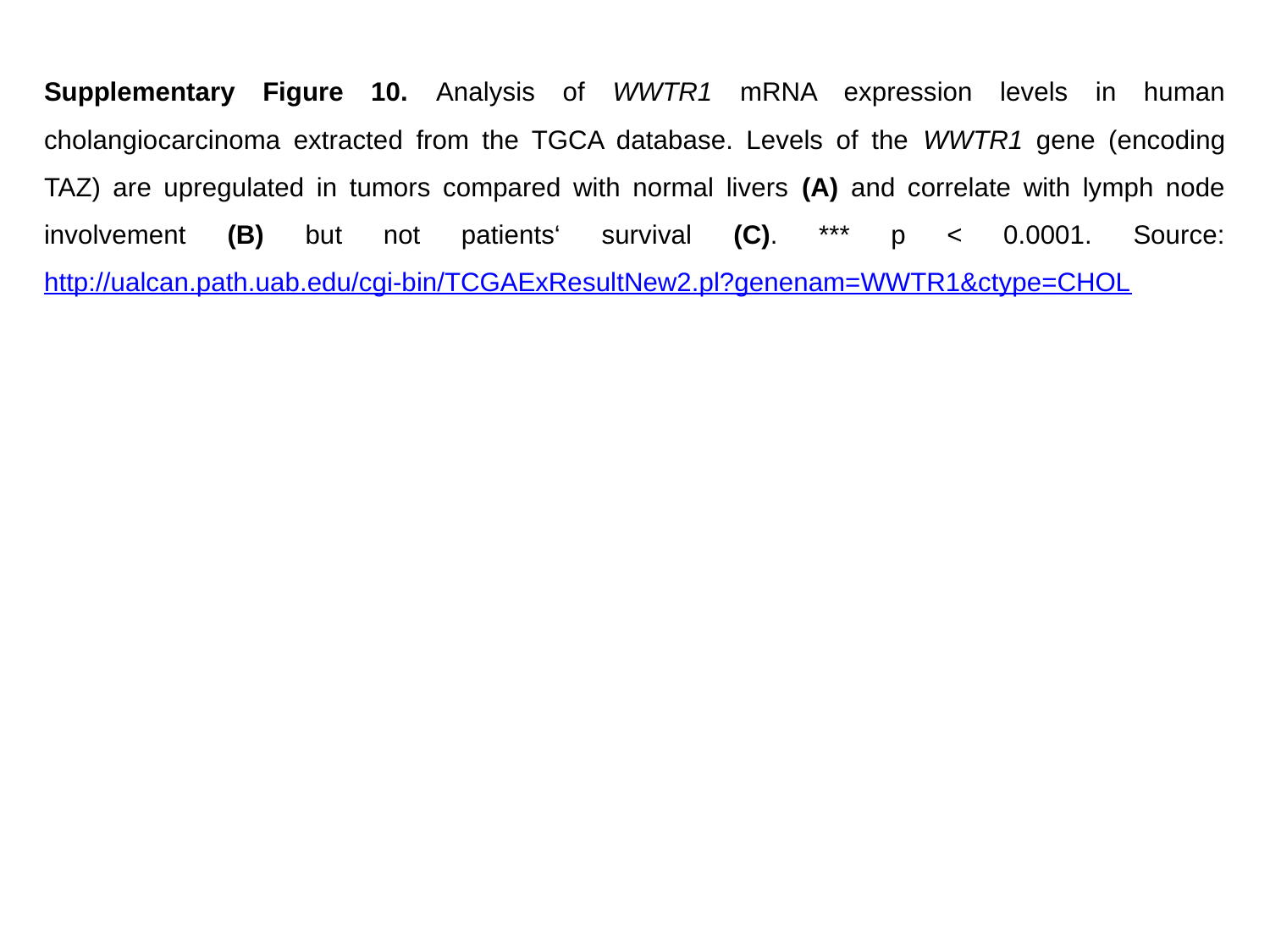

Supplementary Figure 10. Analysis of WWTR1 mRNA expression levels in human cholangiocarcinoma extracted from the TGCA database. Levels of the WWTR1 gene (encoding TAZ) are upregulated in tumors compared with normal livers (A) and correlate with lymph node involvement (B) but not patients‘ survival (C). *** p < 0.0001. Source: http://ualcan.path.uab.edu/cgi-bin/TCGAExResultNew2.pl?genenam=WWTR1&ctype=CHOL
