# Supplementary material for: The Hippo pathway effector TAZ induces intrahepatic cholangiocarcinoma in mice and is ubiquitously activated in the human disease
Source: J Exp Clin Cancer Res. 2022 Jun 3;41:192. doi: 10.1186/s13046-022-02394-2 (PMC9164528; doi:10.1186/s13046-022-02394-2)
Supplement: Supplementary file 11 — Additional file 11. [file 13046_2022_2394_MOESM11_ESM.pptx]

## Slide 1
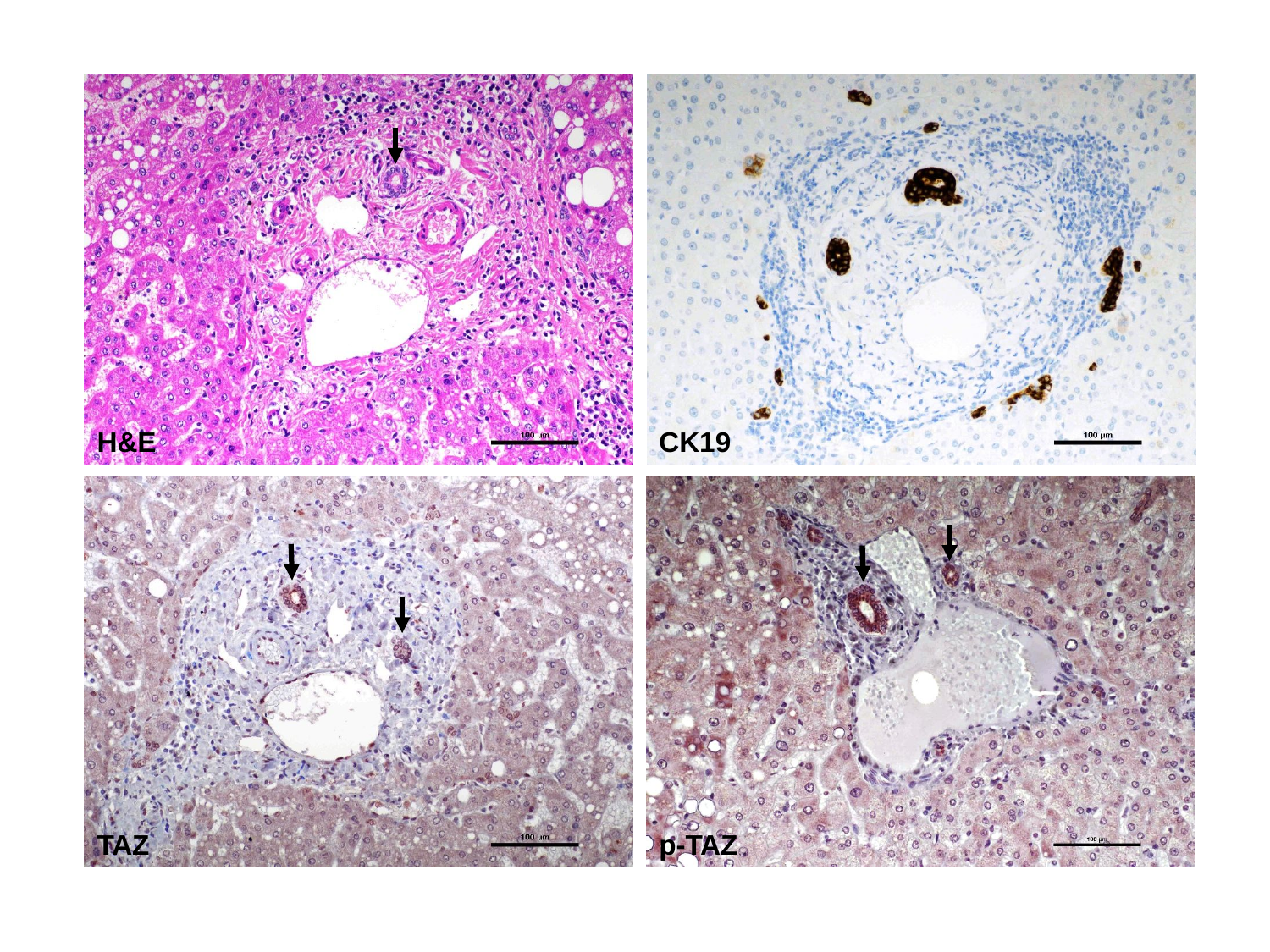

H&E
CK19
TAZ
p-TAZ

## Slide 2
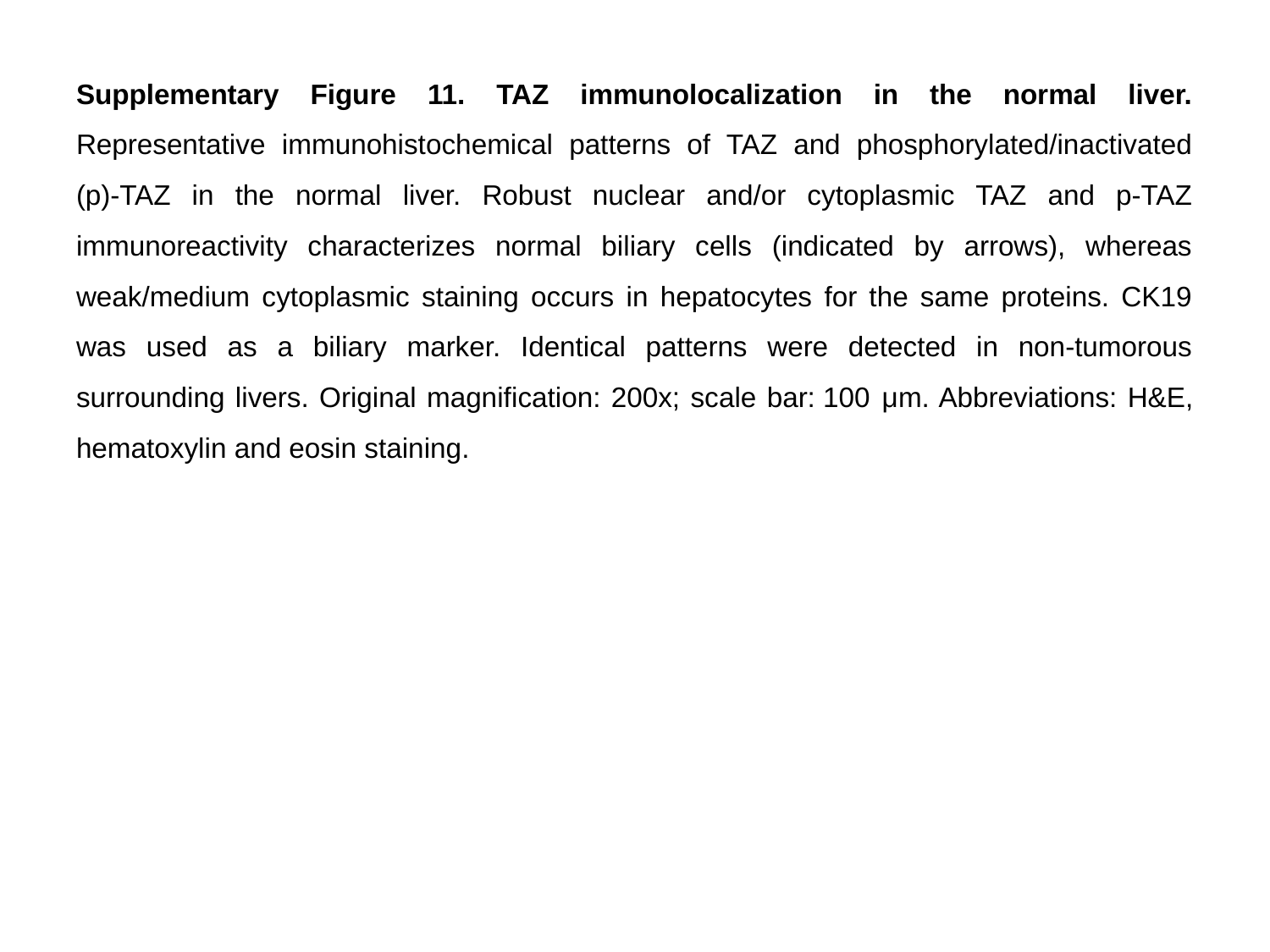

Supplementary Figure 11. TAZ immunolocalization in the normal liver. Representative immunohistochemical patterns of TAZ and phosphorylated/inactivated (p)-TAZ in the normal liver. Robust nuclear and/or cytoplasmic TAZ and p-TAZ immunoreactivity characterizes normal biliary cells (indicated by arrows), whereas weak/medium cytoplasmic staining occurs in hepatocytes for the same proteins. CK19 was used as a biliary marker. Identical patterns were detected in non-tumorous surrounding livers. Original magnification: 200x; scale bar: 100 μm. Abbreviations: H&E, hematoxylin and eosin staining.
