# Supplementary material for: The Hippo pathway effector TAZ induces intrahepatic cholangiocarcinoma in mice and is ubiquitously activated in the human disease
Source: J Exp Clin Cancer Res. 2022 Jun 3;41:192. doi: 10.1186/s13046-022-02394-2 (PMC9164528; doi:10.1186/s13046-022-02394-2)
Supplement: Supplementary file 12 — Additional file 12. [file 13046_2022_2394_MOESM12_ESM.pptx]

## Slide 1
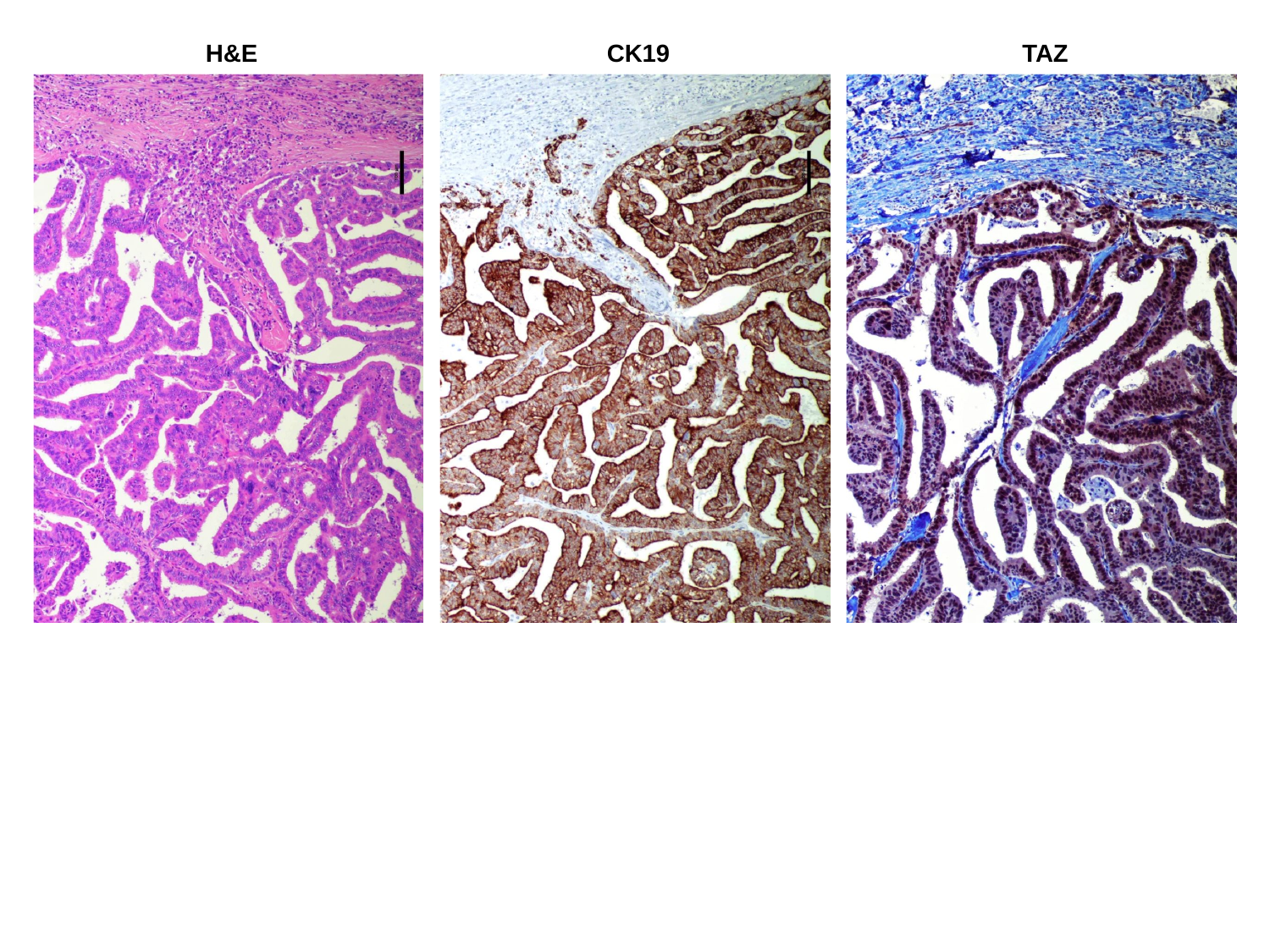

H&E
CK19
TAZ

## Slide 2
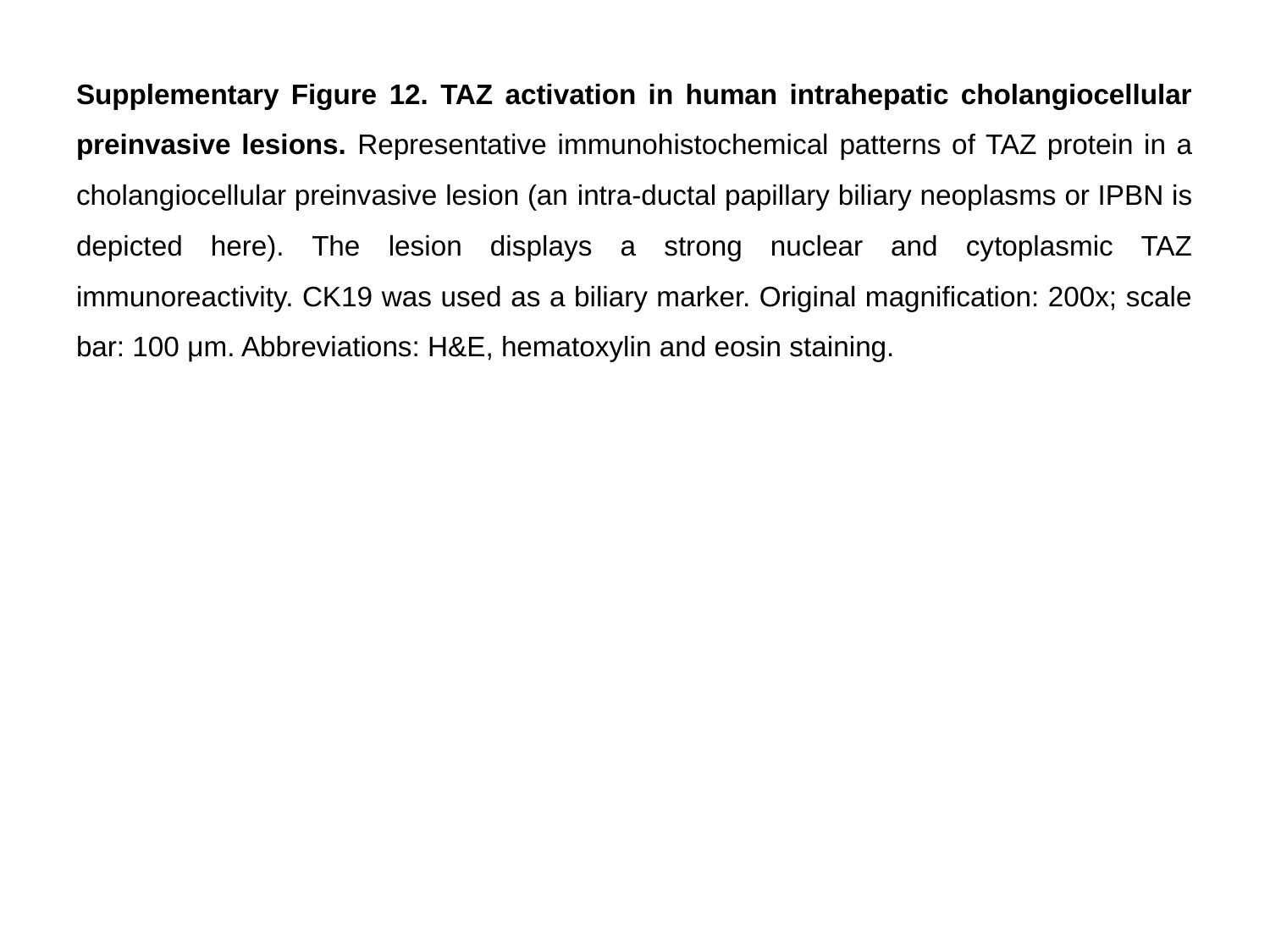

Supplementary Figure 12. TAZ activation in human intrahepatic cholangiocellular preinvasive lesions. Representative immunohistochemical patterns of TAZ protein in a cholangiocellular preinvasive lesion (an intra-ductal papillary biliary neoplasms or IPBN is depicted here). The lesion displays a strong nuclear and cytoplasmic TAZ immunoreactivity. CK19 was used as a biliary marker. Original magnification: 200x; scale bar: 100 μm. Abbreviations: H&E, hematoxylin and eosin staining.
