# Supplementary material for: The Hippo pathway effector TAZ induces intrahepatic cholangiocarcinoma in mice and is ubiquitously activated in the human disease
Source: J Exp Clin Cancer Res. 2022 Jun 3;41:192. doi: 10.1186/s13046-022-02394-2 (PMC9164528; doi:10.1186/s13046-022-02394-2)
Supplement: Supplementary file 13 — Additional file 13. [file 13046_2022_2394_MOESM13_ESM.pptx]

## Slide 1
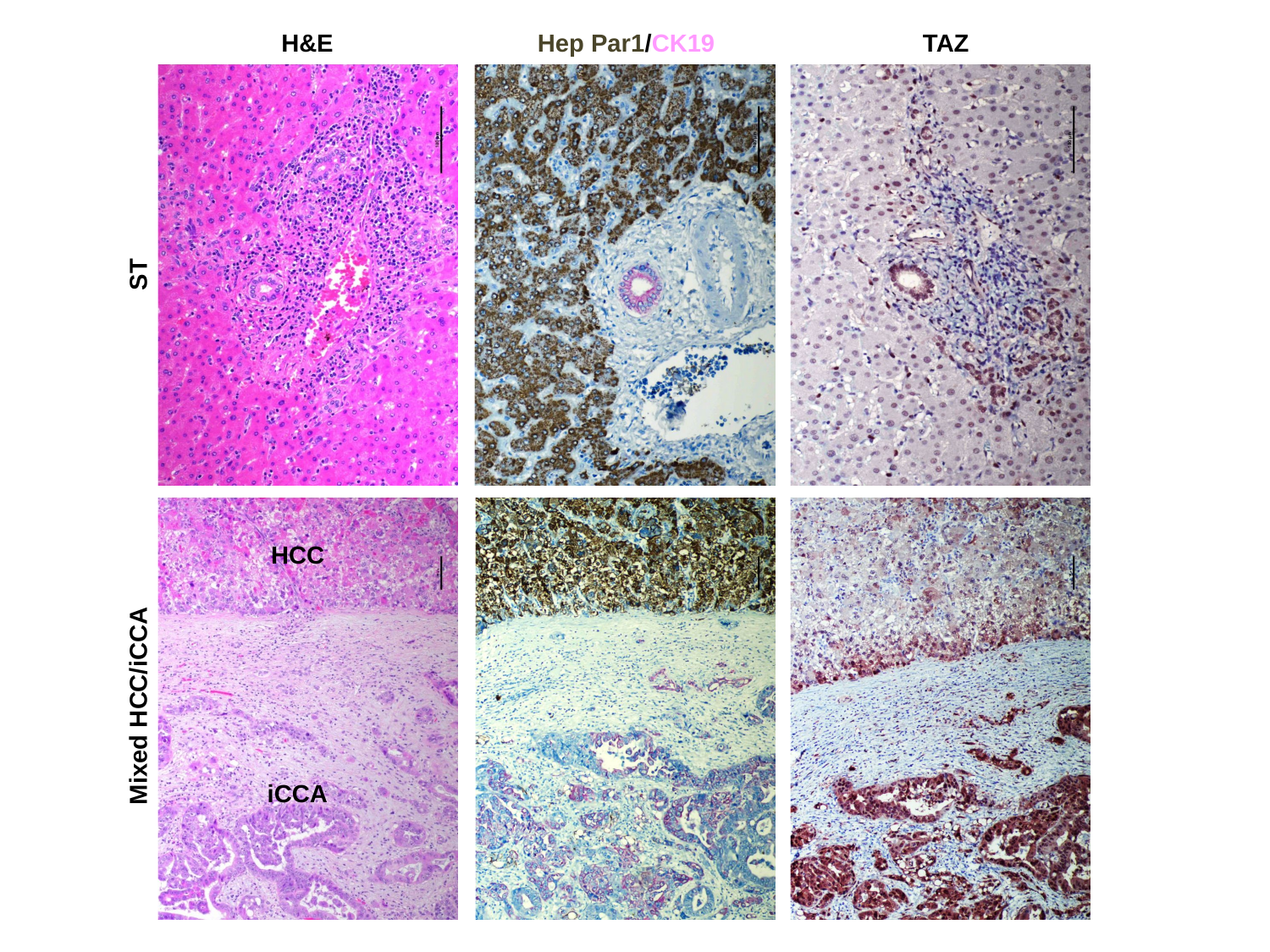

H&E
Hep Par1/CK19
TAZ
ST
HCC
Mixed HCC/iCCA
iCCA

## Slide 2
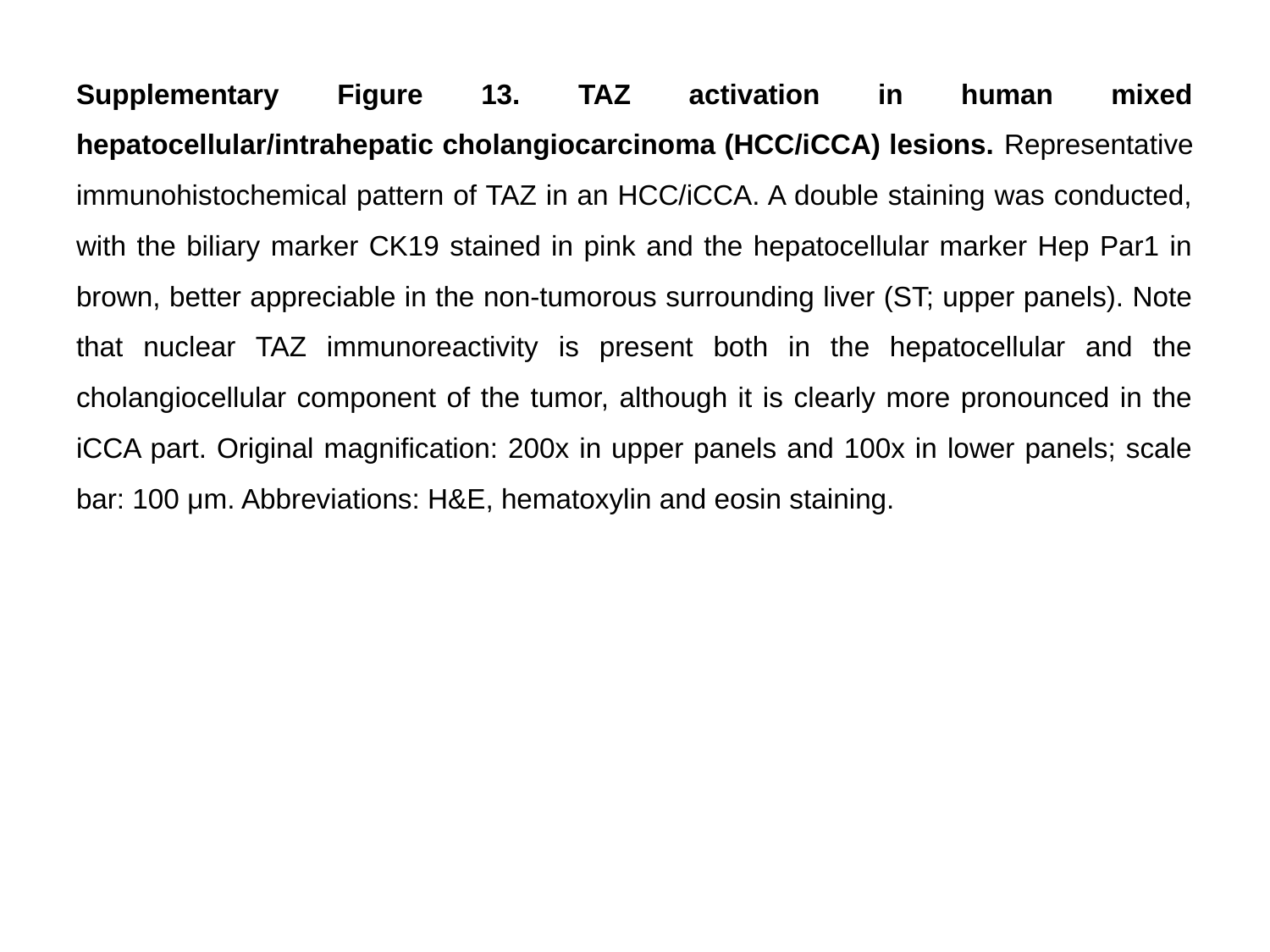

Supplementary Figure 13. TAZ activation in human mixed hepatocellular/intrahepatic cholangiocarcinoma (HCC/iCCA) lesions. Representative immunohistochemical pattern of TAZ in an HCC/iCCA. A double staining was conducted, with the biliary marker CK19 stained in pink and the hepatocellular marker Hep Par1 in brown, better appreciable in the non-tumorous surrounding liver (ST; upper panels). Note that nuclear TAZ immunoreactivity is present both in the hepatocellular and the cholangiocellular component of the tumor, although it is clearly more pronounced in the iCCA part. Original magnification: 200x in upper panels and 100x in lower panels; scale bar: 100 μm. Abbreviations: H&E, hematoxylin and eosin staining.
