# Supplementary material for: The Hippo pathway effector TAZ induces intrahepatic cholangiocarcinoma in mice and is ubiquitously activated in the human disease
Source: J Exp Clin Cancer Res. 2022 Jun 3;41:192. doi: 10.1186/s13046-022-02394-2 (PMC9164528; doi:10.1186/s13046-022-02394-2)
Supplement: Supplementary file 14 — Additional file 14. [file 13046_2022_2394_MOESM14_ESM.pptx]

## Slide 1
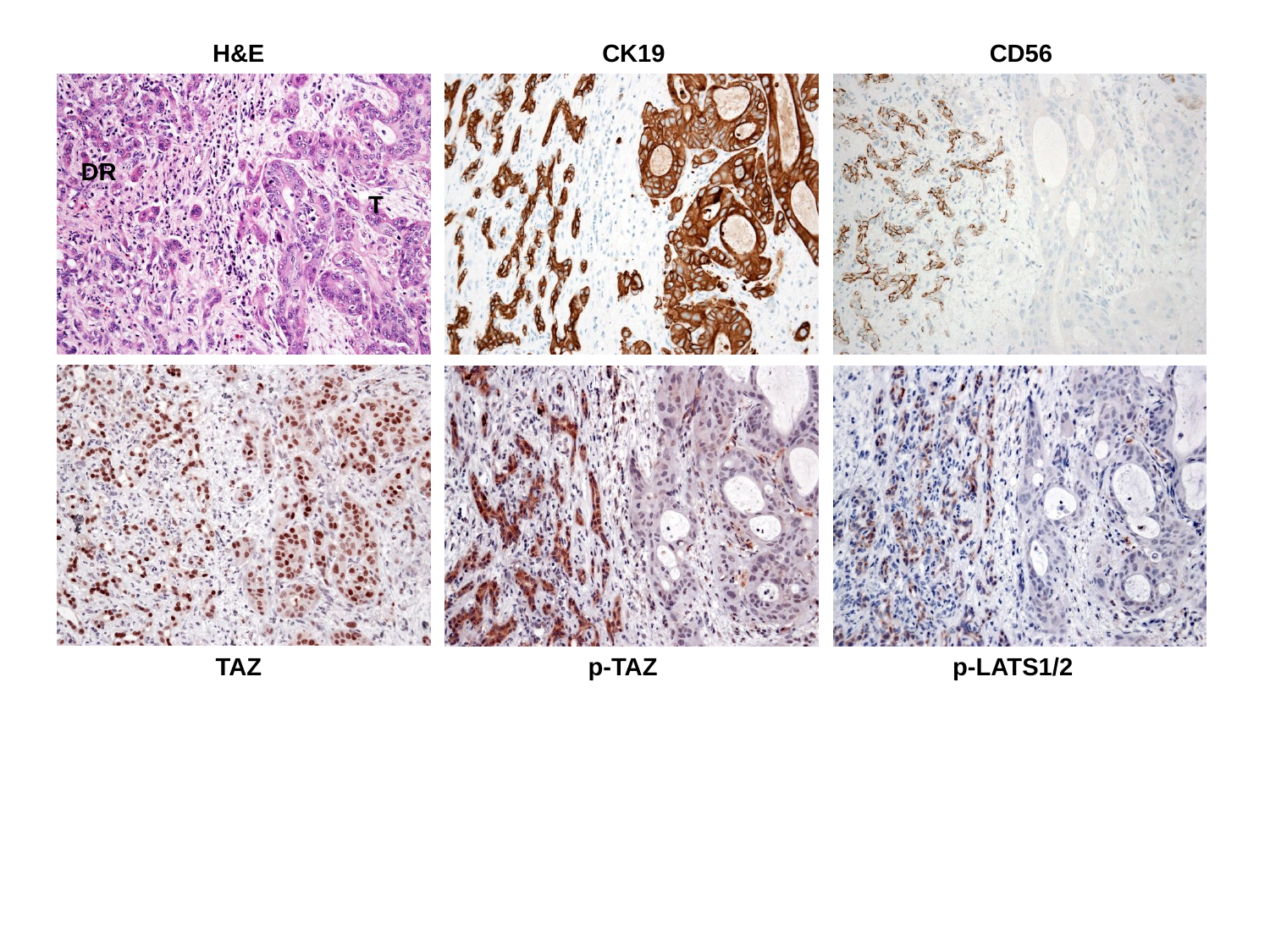

H&E
CK19
CD56
DR
T
TAZ
p-TAZ
p-LATS1/2

## Slide 2
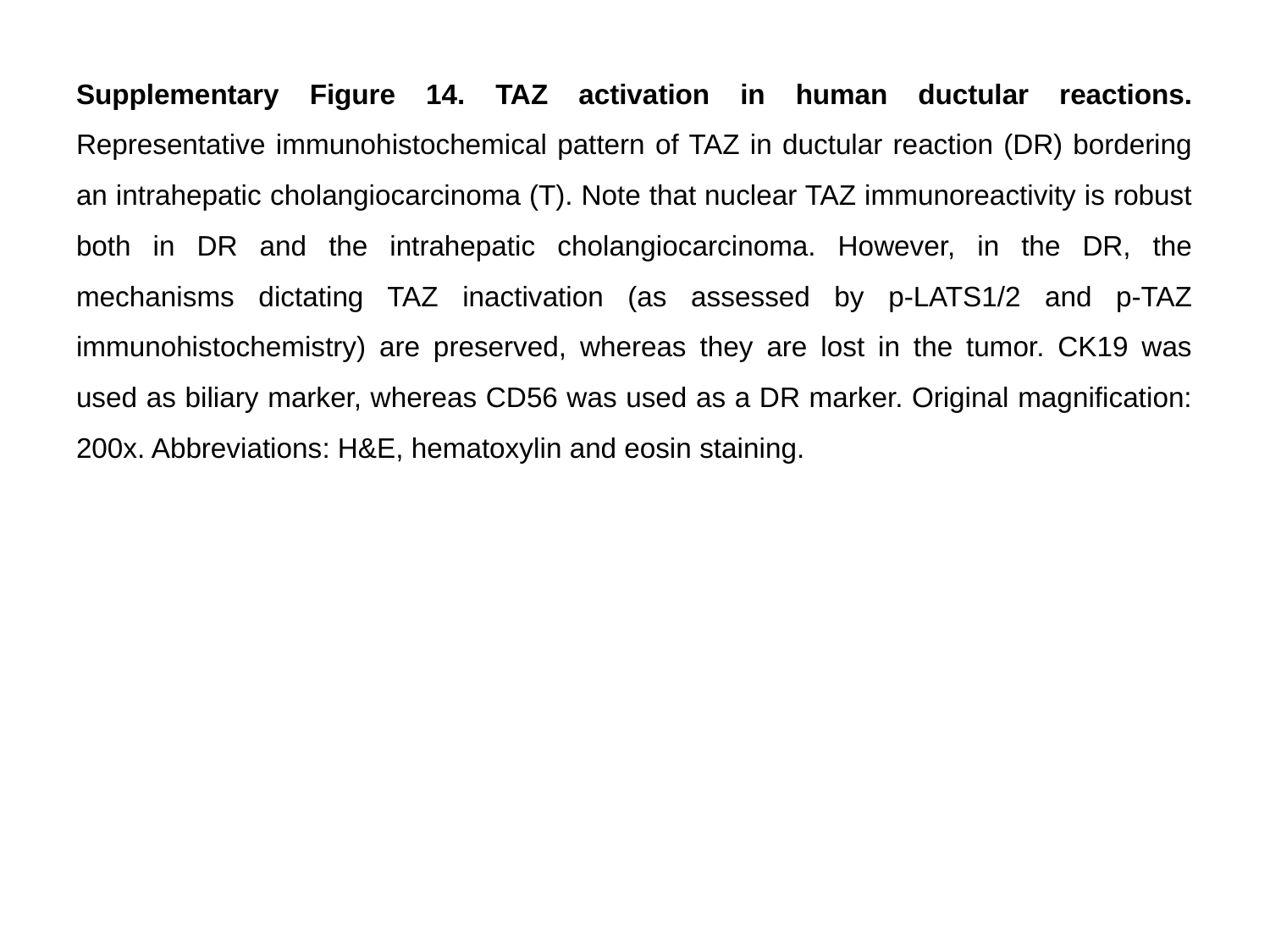

Supplementary Figure 14. TAZ activation in human ductular reactions. Representative immunohistochemical pattern of TAZ in ductular reaction (DR) bordering an intrahepatic cholangiocarcinoma (T). Note that nuclear TAZ immunoreactivity is robust both in DR and the intrahepatic cholangiocarcinoma. However, in the DR, the mechanisms dictating TAZ inactivation (as assessed by p-LATS1/2 and p-TAZ immunohistochemistry) are preserved, whereas they are lost in the tumor. CK19 was used as biliary marker, whereas CD56 was used as a DR marker. Original magnification: 200x. Abbreviations: H&E, hematoxylin and eosin staining.
