# Supplementary material for: The Hippo pathway effector TAZ induces intrahepatic cholangiocarcinoma in mice and is ubiquitously activated in the human disease
Source: J Exp Clin Cancer Res. 2022 Jun 3;41:192. doi: 10.1186/s13046-022-02394-2 (PMC9164528; doi:10.1186/s13046-022-02394-2)
Supplement: Supplementary file 15 — Additional file 15. [file 13046_2022_2394_MOESM15_ESM.pptx]

## Slide 1
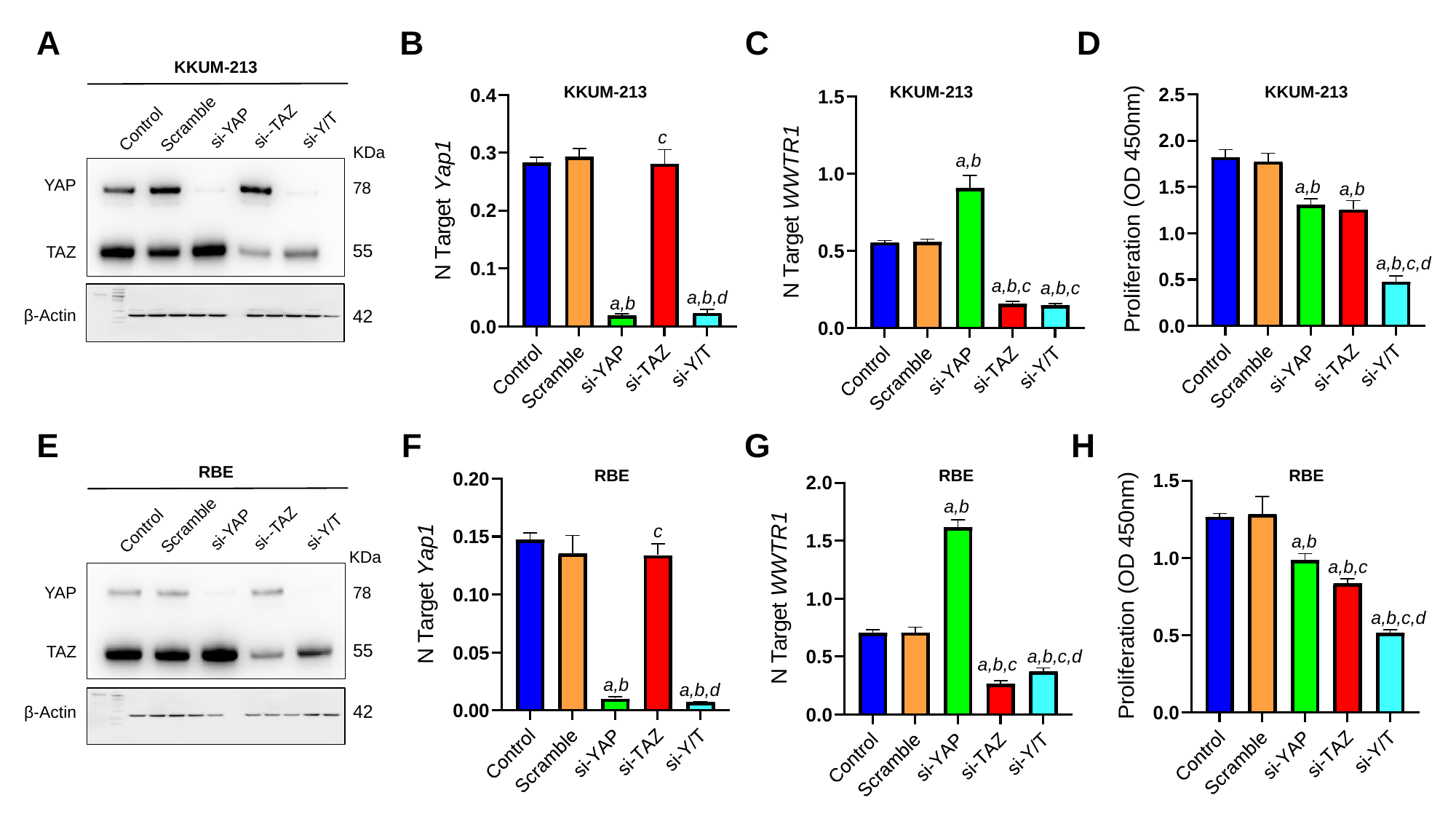

A
B
C
D
KKUM-213
KKUM-213
KKUM-213
KKUM-213
Scramble
Control
si-Y/T
si--TAZ
si-YAP
c
KDa
a,b
YAP
a,b
78
a,b
55
TAZ
a,b,c,d
a,b,c
a,b,c
a,b,d
a,b
42
β-Actin
E
F
G
H
RBE
RBE
RBE
RBE
a,b
Scramble
si-Y/T
si--TAZ
Control
si-YAP
c
a,b
KDa
a,b,c
YAP
78
a,b,c,d
55
TAZ
a,b,c,d
a,b,c
a,b
a,b,d
42
β-Actin

## Slide 2
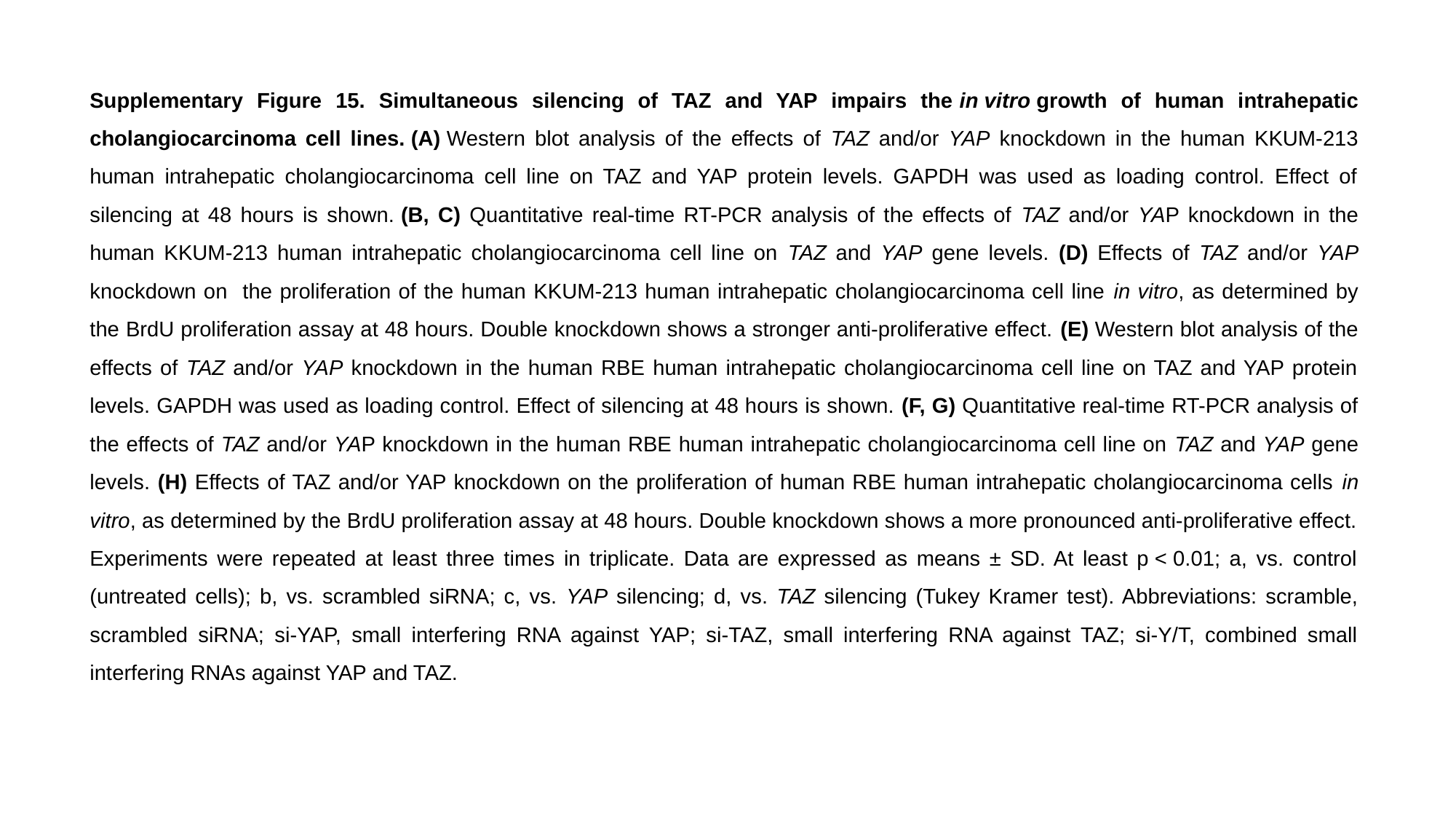

Supplementary Figure 15. Simultaneous silencing of TAZ and YAP impairs the in vitro growth of human intrahepatic cholangiocarcinoma cell lines. (A) Western blot analysis of the effects of TAZ and/or YAP knockdown in the human KKUM-213 human intrahepatic cholangiocarcinoma cell line on TAZ and YAP protein levels. GAPDH was used as loading control. Effect of silencing at 48 hours is shown. (B, C) Quantitative real-time RT-PCR analysis of the effects of TAZ and/or YAP knockdown in the human KKUM-213 human intrahepatic cholangiocarcinoma cell line on TAZ and YAP gene levels. (D) Effects of TAZ and/or YAP knockdown on the proliferation of the human KKUM-213 human intrahepatic cholangiocarcinoma cell line in vitro, as determined by the BrdU proliferation assay at 48 hours. Double knockdown shows a stronger anti-proliferative effect. (E) Western blot analysis of the effects of TAZ and/or YAP knockdown in the human RBE human intrahepatic cholangiocarcinoma cell line on TAZ and YAP protein levels. GAPDH was used as loading control. Effect of silencing at 48 hours is shown. (F, G) Quantitative real-time RT-PCR analysis of the effects of TAZ and/or YAP knockdown in the human RBE human intrahepatic cholangiocarcinoma cell line on TAZ and YAP gene levels. (H) Effects of TAZ and/or YAP knockdown on the proliferation of human RBE human intrahepatic cholangiocarcinoma cells in vitro, as determined by the BrdU proliferation assay at 48 hours. Double knockdown shows a more pronounced anti-proliferative effect. Experiments were repeated at least three times in triplicate. Data are expressed as means ± SD. At least p < 0.01; a, vs. control (untreated cells); b, vs. scrambled siRNA; c, vs. YAP silencing; d, vs. TAZ silencing (Tukey Kramer test). Abbreviations: scramble, scrambled siRNA; si-YAP, small interfering RNA against YAP; si-TAZ, small interfering RNA against TAZ; si-Y/T, combined small interfering RNAs against YAP and TAZ.
