# Supplementary material for: The Hippo pathway effector TAZ induces intrahepatic cholangiocarcinoma in mice and is ubiquitously activated in the human disease
Source: J Exp Clin Cancer Res. 2022 Jun 3;41:192. doi: 10.1186/s13046-022-02394-2 (PMC9164528; doi:10.1186/s13046-022-02394-2)
Supplement: Supplementary file 16 — Additional file 16. [file 13046_2022_2394_MOESM16_ESM.pptx]

## Slide 1
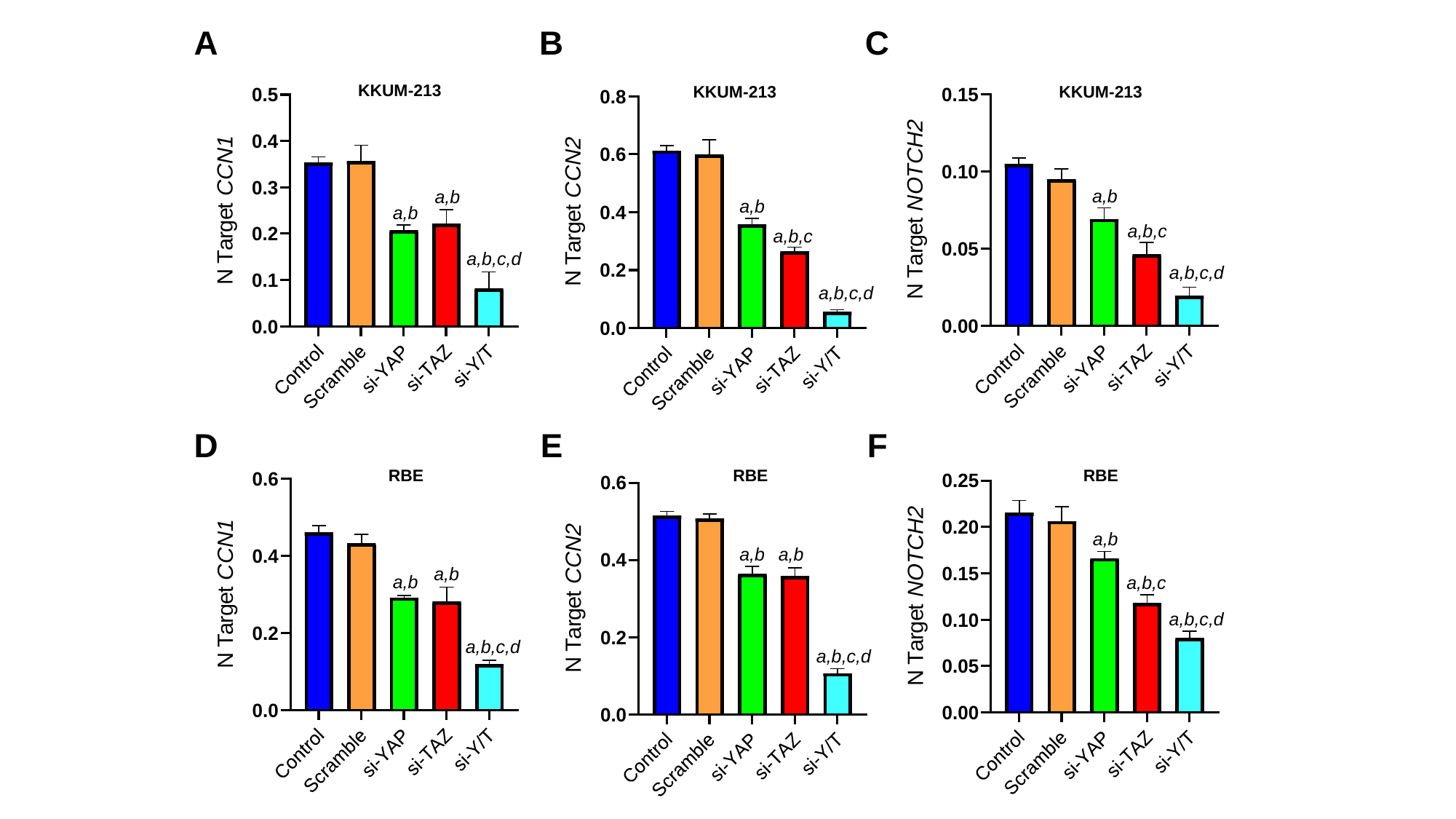

A
B
C
KKUM-213
KKUM-213
KKUM-213
a,b
a,b
a,b
a,b
a,b,c
a,b,c
a,b,c,d
a,b,c,d
a,b,c,d
D
E
F
RBE
RBE
RBE
a,b
a,b
a,b
a,b
a,b
a,b,c
a,b,c,d
a,b,c,d
a,b,c,d

## Slide 2
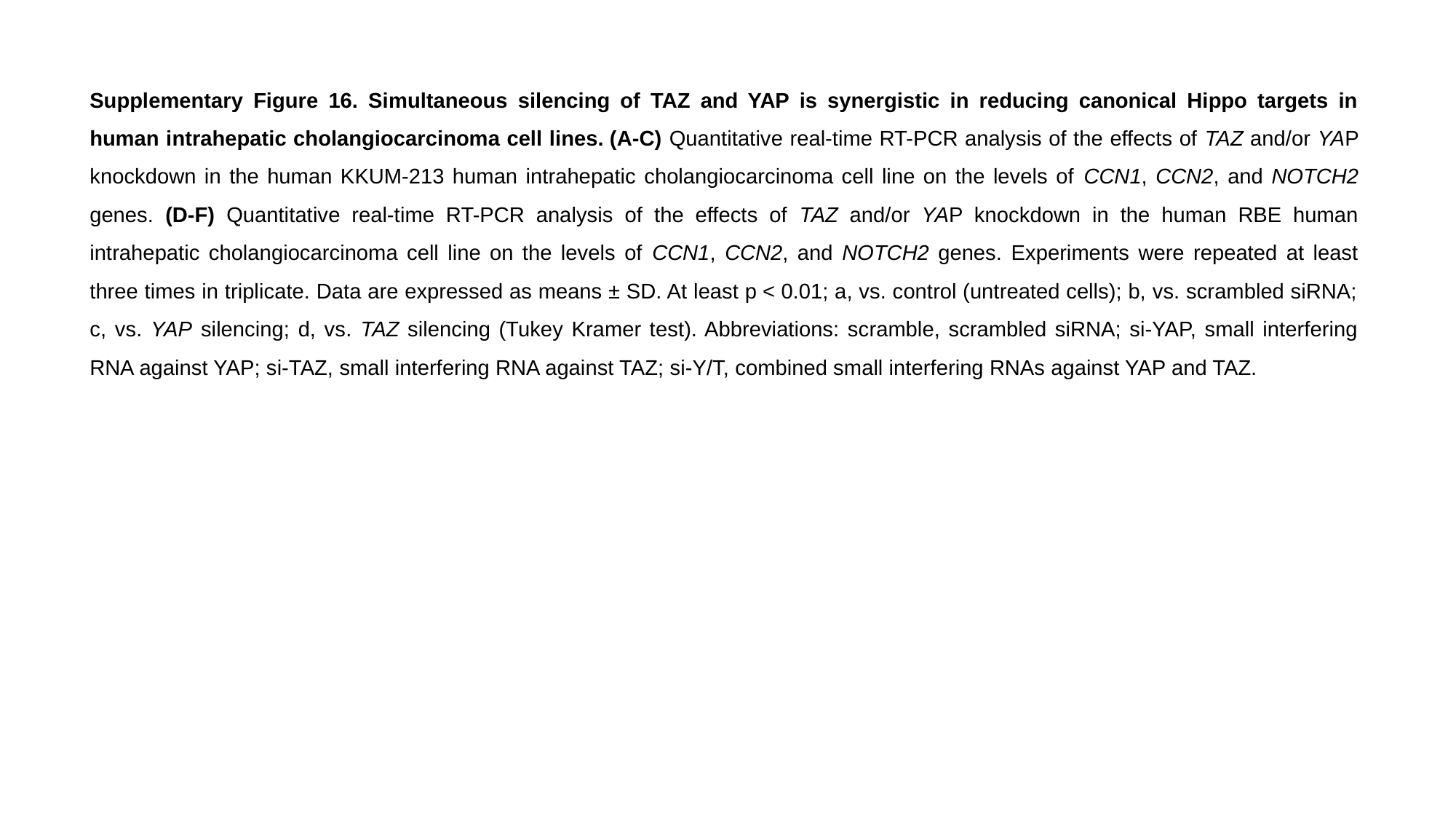

Supplementary Figure 16. Simultaneous silencing of TAZ and YAP is synergistic in reducing canonical Hippo targets in human intrahepatic cholangiocarcinoma cell lines. (A-C) Quantitative real-time RT-PCR analysis of the effects of TAZ and/or YAP knockdown in the human KKUM-213 human intrahepatic cholangiocarcinoma cell line on the levels of CCN1, CCN2, and NOTCH2 genes. (D-F) Quantitative real-time RT-PCR analysis of the effects of TAZ and/or YAP knockdown in the human RBE human intrahepatic cholangiocarcinoma cell line on the levels of CCN1, CCN2, and NOTCH2 genes. Experiments were repeated at least three times in triplicate. Data are expressed as means ± SD. At least p < 0.01; a, vs. control (untreated cells); b, vs. scrambled siRNA; c, vs. YAP silencing; d, vs. TAZ silencing (Tukey Kramer test). Abbreviations: scramble, scrambled siRNA; si-YAP, small interfering RNA against YAP; si-TAZ, small interfering RNA against TAZ; si-Y/T, combined small interfering RNAs against YAP and TAZ.
