# Supplementary material for: The Hippo pathway effector TAZ induces intrahepatic cholangiocarcinoma in mice and is ubiquitously activated in the human disease
Source: J Exp Clin Cancer Res. 2022 Jun 3;41:192. doi: 10.1186/s13046-022-02394-2 (PMC9164528; doi:10.1186/s13046-022-02394-2)
Supplement: Supplementary file 17 — Additional file 17. [file 13046_2022_2394_MOESM17_ESM.docx]

The analysis included data from 50 patients with human cholangiocarcinoma (all with survival data). The variables have been analysed using the Statistical Package for Social Science (SPSS, version 28.0, Chicago, IL, USA).

Descriptive statistics

| **Sex** | **Number (%)** | **Mean survival (SD)** | **Mean WWTR1 mRNA (SD)** |
| --- | --- | --- | --- |
| Male | 30 (60.0) | 26.93 (14.80) | 0.503138 (0.364840) |
| Female | 20 (40.0) | 25.90 (15.23) | 0.480758 (0.279903) |
| Total | 50 | 26.52 (14.83) | 0.494186 (0.330575) |

Mean survival is 26.52 months (SD 14.83)

(differences between males and females are not statistically significant)

**WWTR1 mRNA in human cholangiocarcinoma**

Low and high *WWTR1 mRNA* values were recoded into binary variables (0/1) using the median values (WWTR1 mRNA = 0.4479) as the cut-off. The whole dataset was then divided into 25 subjects with values of WWTR1 mRNA below the median, and 25 subjects above the median. Statistical comparison between the two groups was performed using the **log-rank test**:

| **Marker** | **Number of subjects (%)** | **Mean survival in months (SD)** | **Log-rank test** |
| --- | --- | --- | --- |
| WWTR1 mRNA < 0.4479 | 25 (50.0) | 30.12 (15.30) | ‒ |
| WWTR1 mRNA ≥ 0.4479 | 25 (50.0) | 22.92 (13.71) | 0.138 |
| Total | 50 | 26.52 (14.83) |  |

Conclusion 1: *patients with WWTR1 mRNA values above the median 0.4479* ***did not*** ***survive, on average, significantly shorter than*** *patients with WWTR1 mRNA* *below 0.4479 (see Kaplan-Meier curve)*

**Survival analysis (univariate)**

| **Variable** | **Survival (months) (SD)** | **p-value** |
| --- | --- | --- |
| *Age (years)*  < 65  ≥ 65 | 25.76 (14.71)  27.28 (15.21) | 0.721 |
| *Sex*  Female  Male | 26.93 (14.80)  25.90 (15.23) | 0.812 |
| *Cirrhosis*  No  Yes | 27.63 (13.75)  24.85 (16.54) | 0.521 |
| *Etiology*  HBV  HCV  Hepatolithiasis | 26.85 (19.56)  30.11 (14.46)  25.50 (10.51) | 0.826^#^ |
| *Diameter*  < 5 cm  ≥ 5 cm | 25.85 (14.18)  29.20 (17.76) | 0.528 |
| *Lymph Node Metastasis*  No  Yes | 31.33 (13.64)  19.57 (14.59) | **0.010** |
| *Lung Metastasis*  No  Yes | 28.77 (14.55)  12.71 (7.43) | **0.007** |
| *Differentiation*  Well  Moderately  Poorly | 26.25 (17.35)  23.68 (11.06)  31.91 (15.44) | 0.348^#^ |
| *Tumor number*  Single  Multiple | 29.49 (13.81)  18.07 (14.87) | **0.015** |
| *WWTR1 mRNA*  < 0.4478  ≥ 0.4478 | 30.12 (15.30)  22.92 (13.71) | 0.086 |

^#^One-way ANOVA

In the univariate analysis, a significant difference in survival was found only for lymph node metastasis, lung metastasis, and tumor number.

**Multivariable Cox regression analysis**

A multivariable Cox proportional hazard models was created with survival as the outcome variable. WWTR1 mRNA was included as a predictor together with the covariates (full model). Hazard ratios (HRs) and their 95% confidence intervals were calculated, and the Wald test was used for model testing.

| **Covariates** | **Full model**  **(HR^#^ and 95% CI)** |
| --- | --- |
| Male sex | 1.638 (0.685‒3.917) |
| Age | 0.981 (0.945‒1.018) |
| Cirrhosis (y/n) | 1.933 (0.815‒4.589) |
| *Etiology*^§^  HBV  HCV  Hepatolithiasis | Reference  1.873 (0.508‒6.902)  2.734 (0.758‒9.864) |
| Size ≥ 5 cm | 0.644 (0.250‒1.655) |
| *Lymph Node Metastasis*  No  Yes | Reference  2.068 (0.773‒5.534) |
| *Lung Metastasis*  No  Yes | Reference  4.545 (1.304‒15.84) * |
| *Differentiation*  Well  Moderately  Poorly | Reference  1.564 (0.643‒3.803)  1.352 (0.464‒3.941) |
| *Tumor number*  Single  Multiple | Reference  1.268 (0.383‒4.202) |
| WWTR1 mRNA ≥ 0.4479 (median value) | 1.209 (0.495‒2.953) |

*p<0.05; **p<0.001; ^§^HR for PSC was not calculated since it was present in only one patient

Conclusion: ***Only lung metastasis was an independent predictor of reduced survival in the full model. The other variables (including WWTR1) did not show a significant association.* Correlation of *WWTR1* mRNA levels with clinical parameters**

| **Variable** | **WWTR1 mRNA (SD)** | **p-value** |
| --- | --- | --- |
| *Age (years)*  < 65  ≥ 65 | 0.4182 (0.2995)  0.5701 (0.3483) | 0.105 |
| *Sex*  Female  Male | 0.4807 (0.2799)  0.5031 (0.3648) | 0.817 |
| *Cirrhosis*  No  Yes | 0.5019 (0.3051)  0.4826 (0.3735) | 0.842 |
| *Etiology*  HBV  HCV  Hepatolithiasis | 0.5463 (0.3722)  0.3996 (0.2213)  0.4152 (0.2439) | 0.564^#^ |
| *Diameter*  < 5 cm  ≥ 5 cm | 0.4735 (0.3070)  0.5766 (0.4209) | 0.383 |
| *Lymph Node Metastasis*  No  Yes | 0.4003 (0.2958)  0.6570 (0.3463) | **0.014** |
| *Lung Metastasis*  No  Yes | 0.4607 (0.3369)  0.6996 (0.2019) | 0.076 |
| *Differentiation*  Well  Moderately  Poorly | 0.4808 (0.2830)  0.5622 (0.3265)  0.4010 (0.4157) | 0.434^#^ |
| *Tumor number*  Single  Multiple | 0.4636 (0.3467)  0.5811 (0.2729) | 0.275 |

^#^One-way ANOVA

A significant difference in *WWTR1* mRNA levels was found only for lymph node metastasis.
